# Revealing the Dynamic Allosteric Changes Required for Formation of the Cysteine Synthase Complex by Hydrogen-Deuterium Exchange MS

**DOI:** 10.1016/j.mcpro.2021.100098

**Published:** 2021-05-19

**Authors:** Brenda Rosa, Eleanor R. Dickinson, Marialaura Marchetti, Barbara Campanini, Barbara Pioselli, Stefano Bettati, Kasper Dyrberg Rand

**Affiliations:** 1 Biopharmanet-TEC Interdepartmental Center, University di Parma, Parma, Italy; 2 Protein Analysis Group, Department of Pharmacy, University of Copenhagen, Copenhagen O, Denmark; 3 Department of Food and Drug, University of Parma, Parma, Italy; 4 R & D Department, Chiesi Farmaceutici, Parma, Italy; 5 Department of Medicine and Surgery, University of Parma, Parma, Italy; 6 Institute of Biophysics, CNR, Pisa, Italy

**Keywords:** cysteine biosynthesis, bienzyme complex, protein–protein interactions, allosteric regulation, cysteine synthase, BE, back exchange, CI, confidence interval, CS, cysteine synthase, DDA, data-dependent acquisition, HDX-MS, hydrogen/deuterium exchange MS, OAS, *O*-acetylserine, PDB, Protein Data Bank, SAXS, small-angle X-ray scattering

## Abstract

CysE and CysK, the last two enzymes of the cysteine biosynthetic pathway, engage in a bienzyme complex, cysteine synthase, with yet incompletely characterized three-dimensional structure and regulatory function. Being absent in mammals, the two enzymes and their complex are attractive targets for antibacterial drugs. We have used hydrogen/deuterium exchange MS to unveil how complex formation affects the conformational dynamics of CysK and CysE. Our results support a model where CysE is present in solution as a dimer of trimers, and each trimer can bind one CysK homodimer. When CysK binds to one CysE monomer, intratrimer allosteric communication ensures conformational and dynamic symmetry within the trimer. Furthermore, a long-range allosteric signal propagates through CysE to induce stabilization of the interface between the two CysE trimers, preparing the second trimer for binding the second CysK with a nonrandom orientation. These results provide new molecular insights into the allosteric formation of the cysteine synthase complex and could help guide antibacterial drug design.

## Highlights

• HDX-MS reveals complex formation impact on conformational dynamics of CysK and CysE.
• CysK binding ensures conformational symmetry within the CysE trimer.
• Long-range allostery propagates through CysE stabilizing the inter-trimer interface.
• Insights into the allostery of CS complex could help guide antibacterial drug design.

## Graphical Abstract

**Figure:**
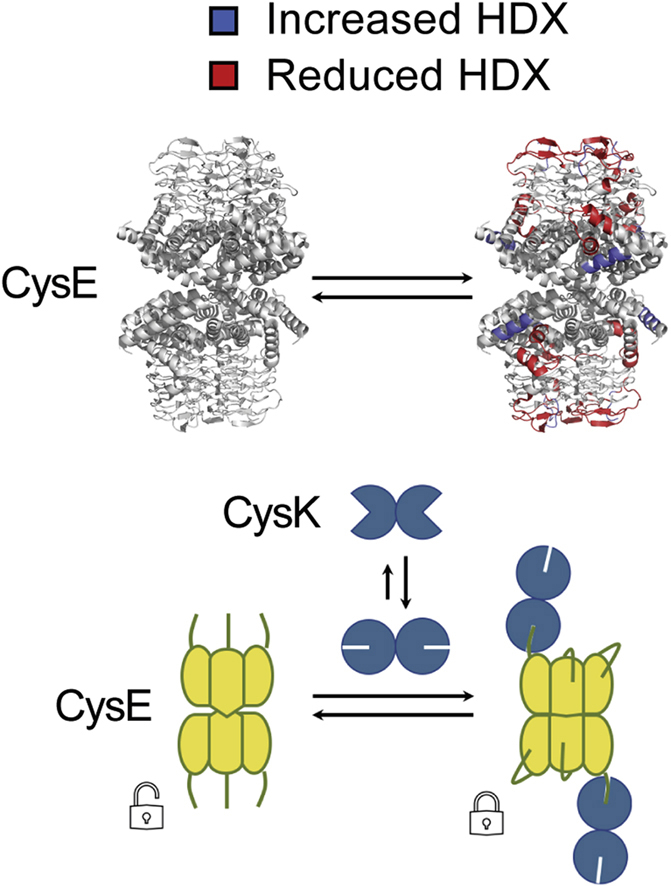

## In Brief

We have used hydrogen/deuterium exchange MS to unveil the allosteric changes occurring during complex formation of CysE and CysK, the last two enzymes of cysteine biosynthetic pathway in bacteria. Significant changes in conformation and dynamics occur in each protein upon complex formation, including long distance intraprotein and interprotein communication. Being absent in mammals, both CysE and CysK represent potential targets for new antibacterial drugs, and our results on the formation and allostery of the complex could help guide drug development.

**Fig. 1:**
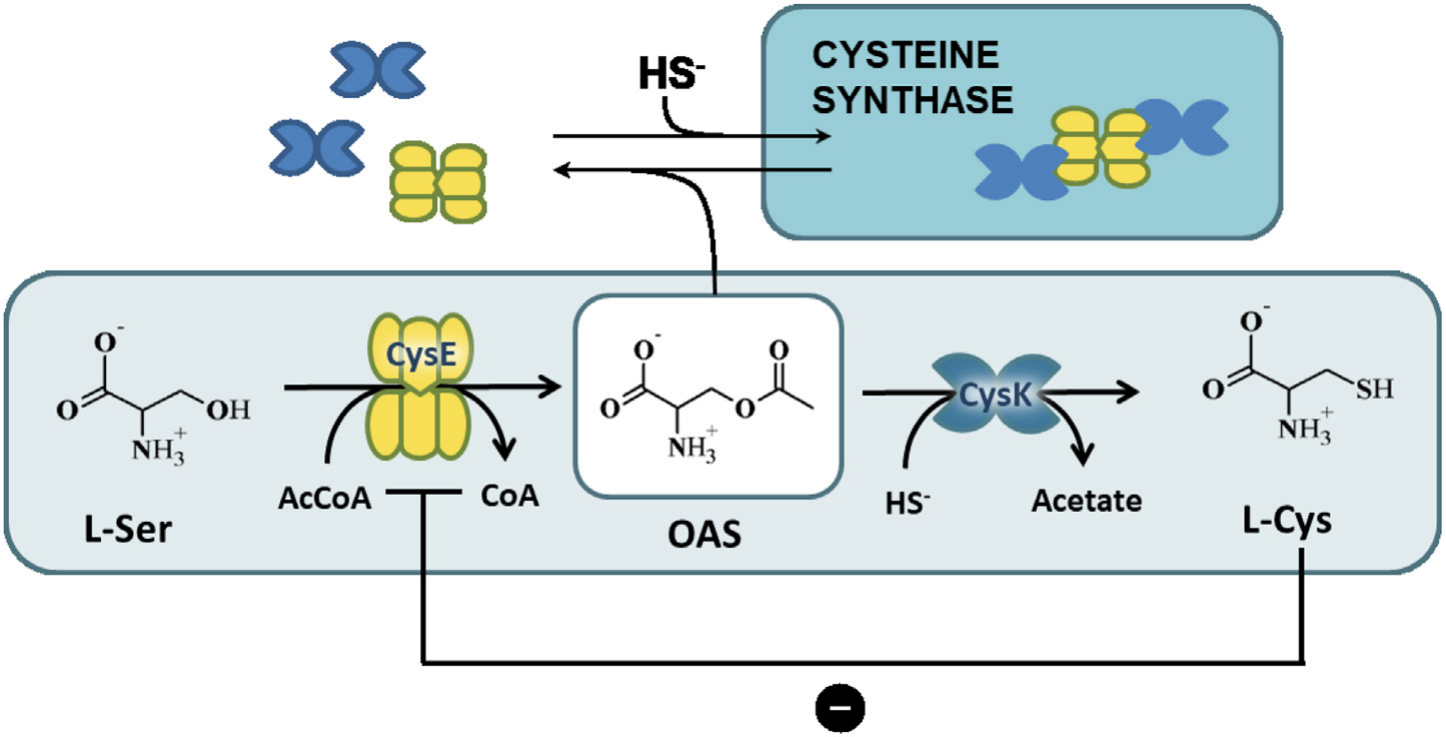
**Cysteine synthase complex assembly and reactions catalyzed by the constituent enzymes (adapted from Ref.** (11)**)**.

Cysteine is a semiessential amino acid for humans and is the form in which inorganic sulfur is assimilated by bacteria and made available for a number of processes, ranging from cofactor and nucleotide biosynthesis to oxidative stress defense. Therefore, molecular details of the pathways for cysteine biosynthesis and its regulation in bacteria are of paramount importance and have many relevant applications. These range from the optimization of the industrial production of this amino acid to the exploitation of its inhibition in the development of enhancers of antibacterial therapy. Serine, synthesized from the glycolytic intermediate 3-phosphoglycerate, is the direct precursor of cysteine. The amino acid is activated by *O*-acetylation by the enzyme serine acetyltransferase, CysE (Fig. 1). The product *O*-acetylserine (OAS) is the substrate for the next enzyme in the pathway, namely OAS sulfhydrylase, isoform A (CysK) (1). CysK catalyzes a β-substitution of OAS with bisulfide to give l-Cys and acetate (2). CysK is a highly dynamic protein, and indeed three structures of the protein have been solved to date that represent the open (Protein Data Bank [PDB] code: 1OAS), inhibited (PDB code: 1FCJ), and closed (PDB code: 1D6S) forms of the enzyme (3, 4, 5). The main structural change in the transition from the open to close state is the closure of the active-site entrance by a rotation of the N-terminal domain on the C-terminal domain, which is triggered by the binding of the carboxy group of the OAS amino acid to its specific subsite in the active site of the enzyme (4). The B-isoform of OAS sulfhydrylase (CysM) can catalyze the β-replacement reaction too, but its contribution to the total cysteine biosynthesis under aerobic conditions is poorly understood (6, 7). CysK fulfils at least two diverse functions in *Escherichia coli*: cysteine biosynthesis and toxin activation (8) and has thus been classified as a moonlighting protein, that is, a protein with multiple biochemical functions (9). CysK activity in cysteine biosynthesis depends on the preceding reaction in the pathway, serine acetylation, catalyzed by CysE. CysE and CysK form a bienzymatic complex (called cysteine synthase [CS]) (Fig. 1) through a very stable interaction, which is stabilized by a quite rare mechanism: the insertion of the C-terminal sequence of CysE into the active site of CysK (10). The name of the complex might be deceiving since CysK is almost totally inhibited when engaged in complex formation, with a residual activity of about 10% (11, 12). The function of the complex is still unknown, but much evidence points to a regulatory function on the relative activity of CysK and CysE (11, 13). However, the details of the regulatory mechanism are still obscure and stand on indirect evidence. It has been clearly demonstrated that only two CysK dimers bind to one CysE hexamer (14, 15, 16, 17), the complex is very stable with a *K*_*d*_ of about 6 nM (11), and the affinity is modulated by cysteine, OAS, and bisulfide (11, 16, 18, 19, 20, 21). OAS in millimolar range dissociates the complex, but this effect is prevented by bisulfide. The effect of cysteine on CysE activity and complex regulation is interesting. In fact, cysteine controls its own biosynthesis by a classical feedback mechanism by inhibition of CysE activity (22). Cysteine binds to the serine-binding site, thus exerting a classical competitive inhibition (23, 24, 25). The binding of cysteine, on the other hand, induces a conformational change in the C-terminal part of CysE that folds back into the active site and occupies the acetyl-CoA binding site with residues 254 to 257 (25). Even more interestingly, this conformational change does not involve equally all the subunits of the hexamer but rather induces an asymmetry through the CysE trimeric interface, with the subunits in one trimer having the C-termini folded back into the active sites and the subunits of the other trimer having the C-termini exposed to the solvent. When CysE is engaged in complex formation, the feedback inhibition by cysteine is relieved (11) likely because the C-terminal sequence is engaged in CysK binding and is no more available for the intrasteric inhibition.

Our previous work has suggested that CysE binds to the closed conformation of CysK. There is also strong experimental indication from structural (15) and functional studies (11, 12) that only one active site of CysK is occupied by CysE, the other one being closed as a consequence of allosteric communication between subunits. However, detailed insights into the conformational dynamics of CysK and CysE alone and when in the mature complex are lacking and if combined with biochemical/functional data, they could greatly deepen our understanding of the role, molecular details, and function of CS complex assembly.

Hydrogen/deuterium exchange MS (HDX-MS) provides a valuable approach to investigate protein dynamics in solution (26, 27). In this technique, the hydrogen to deuterium exchange of backbone amides is detected with MS. The HDX of a backbone amide is very sensitive to the solvation and stability of local hydrogen bonding networks and hence is closely related to protein structure and conformational dynamics (28, 29, 30). Moreover, labeling of a protein with deuterium does not involve alterations of covalent structure, and HDX-MS is thus a largely nonperturbing technique that can report on the native conformational dynamics of a protein in solution (27).

In this work, HDX-MS was applied to map regions of CysK and CysE that undergo dynamic movements and changes in conformation upon CS complex formation. Two kinds of approaches are normally used to perform HDX studies, namely continuous and pulsed labeling (31, 32). Here, we apply a continuous labeling HDX-MS workflow to study the CS complex. Briefly, the protein complex or individual components are diluted into a deuterated buffer and incubated for different periods. The deuterium incorporation is stopped (quenched) by reducing pH and temperature. Quenched samples are proteolytically digested with an acidic protease, such as pepsin, and the resulting peptides are analyzed by LC coupled to MS (LC–MS). The resulting HDX-MS data thus provide information on the exchange of individual segments of the protein (33). Our HDX-MS results identify key changes in conformational dynamics affecting the constituent enzymes of the CS complex upon their association. Importantly, the work sheds new light on the dynamics of CysE upon complex formation, which have been much less characterized than those affecting CysK. A regulatory function of the hexameric assembly of the CS complex has been originally invoked by Hindson *et al.* (34), which might have arisen by the stacking of an ancestral trimer. Here, we demonstrate that complex formation induces allosteric cross talk between monomers of CysE and further causes long-range allosteric changes at the dimer-of-trimers interface that could play a regulatory role. These findings improve our understanding of the regulatory effect and the biological role of CysE assembly with CysK and could aid the development of antimicrobial agents that are able to inhibit or modulate the interaction of CysK with CysE.

## Experimental Procedures

### Materials

All chemicals and reagents were purchased from Sigma–Aldrich in analytical grade except the following: immobilized pepsin beads (Thermo Scientific) and acetonitrile (Biosolve). Materials were used as received.

### Protein Expression and Purification

Expression vectors for CysK and CysE from *E. coli* were a kind gift of Professor Christopher Hayes, University of California, Santa Barbara (35). Cells were grown in Luria–Bertani medium at 37 °C and induced with 1 mM IPTG. CysK and CysE were then purified, and the tag was removed from CysE following a procedure described in detail in a previous work (11) with minor modifications. Protein purity was assessed by SDS-PAGE and estimated to be higher than 95%. CysK concentration was determined based on the absorbance of the coenzyme pyridoxal 5’-phosphate and calculated by the alkali denaturation method (36). The extinction coefficient was estimated to be 9370 M^−1^·cm^−1^ at 412 nm. CysE concentration was determined using an extinction coefficient of 26,900 M^−1^·cm^−1^ at 280 nm.

### Enzyme Activity Assays

The specific activity of CysK was determined by the discontinuous assay of Gaitonde (37), as described (35), adapted to a 96-well plate format. It corresponded to 280 U/mg. The specific activity of CysE was measured monitoring the disappearance of acetyl-CoA at 232 nm, in the presence of 1 mM l-Ser, 0.25 mM acetyl-CoA, and 7 nM enzyme (monomer), at 20 °C. It corresponded to 83 U/mg (11).

### Spectroscopy

Absorption spectra were collected at 20 ± 0.5 °C using a Varian CARY400 spectrophotometer. All spectra were corrected for buffer contributions. Fluorescence measurements were carried out using a FluoroMax-3 fluorometer (HORIBA Jobin Yvon) at 20 ± 0.5 °C. All samples were equilibrated 5 min to the experimental temperature prior to spectra acquisition. The stoichiometric binding of CysE to CysK was monitored by measuring pyridoxal 5′-phosphate fluorescence emission at 500 nm, following excitation at 412 nm (11, 14, 35, 38). All spectra were corrected for buffer contributions, and the slit width was set to optimize the signal-to-noise ratio.

### HDX

Prior to HDX labeling, CysK and CysE proteins were diluted to 5 μM in 5 μl of equilibration solution (PBS, pH 7.45). For CS complex formation, proteins were diluted to 5 μM CysK and 7.5 μM CysE in 5 μl total volume of equilibration buffer and incubated for 30 min at room temperature. The relative concentration of the proteins in the complex was chosen according to the known binding stoichiometry of 2:3 (14). The HDX reactions were initiated by diluting the samples 1:9 with 99% deuterated PBS buffer at 25 °C. According to these experimental conditions, more than 90% of CysE will be assembled with CysK. Proteins were labeled for the following periods: 0.03, 1.67, 16.67, 60, and 720 min. For the shorter deuterium labeling time (0.03 min), two people were required for sample preparation. In accordance to community-based guidelines (39), two time points (0.03, 1.67 min) were prepared in triplicate to allow a reasonable estimate of experimental error, whereas the other periods were performed in single replicate. Nondeuterated samples were prepared in triplicate for CysK and one replicate for CysE and CS complex. Maximum deuterated controls were prepared for the CS complex in triplicate. About 5 μM CysK and 7.5 μM CysE in 5 μl total volume of equilibration buffer were diluted 1:9 with the deuterated buffer 20 mM Tris (pH 7.4), 6 M guanidinium chloride, and incubated at 37 °C for 24 h. After incubation with deuterium, each sample was quenched by 1:1 dilution into ice-cold quenching buffer (300 mM phosphate buffer, pH 2.3). Quenched samples were immediately frozen at −80 °C and stored until measurements by LC–MS analysis.

### LC–MS

Frozen quenched samples were quickly thawed in a tabletop centrifuge and injected into a refrigerated (0 °C) UPLC system (nanoACQUITY UPLC and HDX Manager; Waters). The samples were subjected to online pepsin digestion at 20 °C through a home-packed column (2 cm in length, 2 mm i.d.) containing pepsin immobilized on agarose beads (Thermo Scientific; 125 μM). The resulting peptides were trapped on a trap column (VanGuard BEH C18 precolumn, 1.7 μm, 2.1 × 5 mm; Waters) and desalted for 3 min at a flow rate of 200 μl/min with solvent A (0.23% formic acid in Milli-Q water, pH 2.5). Subsequently, the peptides were separated over an analytical column (Acquity UPLC BEH C18, 1.7 μm, 1.0 × 100 mm; Waters) at a flow rate of 40 μl/min, with a 9 min linear gradient rising from 8% to 40% solvent B (0.23% formic acid in acetonitrile). Following the chromatographic separation, the peptides were analyzed using a hybrid electrospray ionization-quadrupole-TOF mass spectrometer (Synapt G2-Si; Waters). The MS was operated in positive ionization mode, and the peptides were further separated by ion mobility to enhance peak capacity. Glu-fibrinopeptide B was used for lock mass correction of all spectra. For peptide identification, nondeuterated samples were injected and subjected to two different chromatographic separation conditions: a short gradient, identical to deuterated samples, and a long gradient (35 min linear gradient rising from 5% to 30% solvent B, at a flow rate of 40 μl/min). The chromatographic separation was performed using the same setup as the deuterated samples. MS–MS analyses were performed using a combination of data-independent acquisition/MS^E^ and data-dependent acquisition (DDA) mode.

### HDX-MS Data Analysis

MS–MS data were processed with ProteinLynx Global SERVER, version 3.0 (Waters) for peptide identification. Peptides identified by DDA MS–MS data had to have ProteinLynx Global SERVER Ladder score above 1.0 and a mass error below 15 ppm for the precursor ion. Importantly, manual inspection and quality control of the fragment spectrum for each selected peptide was also done. To increase sequence coverage, peptides similarly identified in DDA MS–MS data acquired using an extended chromatographic gradient (35 min) were added to the final peptide list if their retention time and precursor mass could be in addition identified and verified in data acquired using the same gradient used for the actual HDX-MS experiment. DynamX, version 3.0 (Waters) was used to filter peptides identified by data-independent acquisition, based on the following parameters: minimum two product ions, minimum 0.2 product ions per amino acid, and maximum 10 ppm mass error on precursor ion. Furthermore, the peptides were filtered by identification in a minimum of three of four consecutive MS^E^ runs. Analysis of deuterium uptake of all identified peptides was manually verified. Back exchange (BE) for individual peptides was calculated using the average of the maximum deuterated controls, according to the following equation:

(1) $$BE\left(\%\right)=\left(1-\frac{m_{\max}-m_{0\%}}{N\times D_{frac}}\right)\times 100\%$$

where *m*_*max*_ is the theoretical maximum deuterium uptake of the peptide (N-terminal residues and proline residues are excluded from the calculation), *m*_0%_ corresponds to the mass of the nondeuterated peptide, *N* is the number of amide hydrogens of the peptide, and *D*_*frac*_ is the fraction of deuterium in the HDX-labeling buffer. BE was calculated to validate HDX-MS system performance and support the assignment and analysis of CysK and CysE peptides. The plotted deuterium uptake profiles were not corrected for BE.

**Fig. 2:**
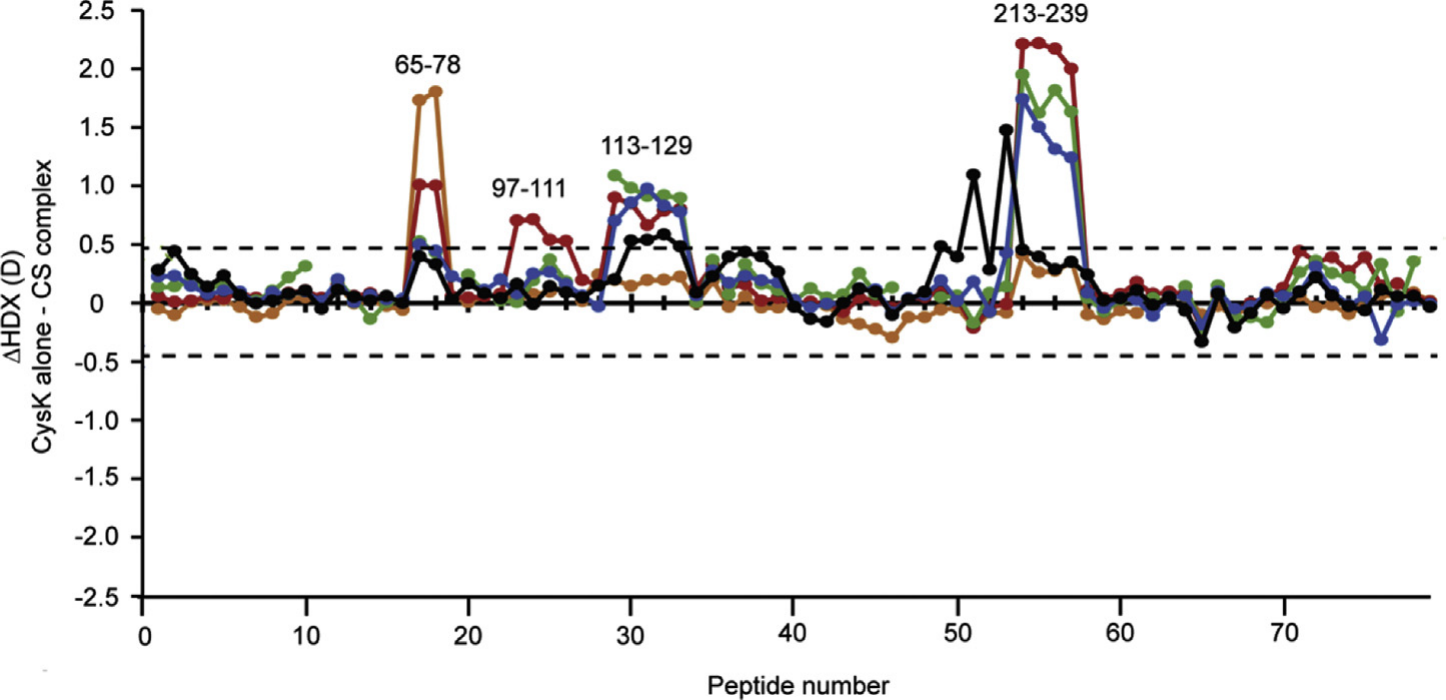
**HDX of unbound and complex-bound CysK.** Difference plot of the deuterium uptake (ΔHDX) between two states: CysK alone and CysK assembled with CysE (CS complex) for the 79 identified peptides at the five measured time points (*orange*—0.03 min; *red*—1.67 min; *green*—16.67 min; *cyan*—60 min; and *black*—720 min). The peptides are arranged along the *x*-axis according to their position in CysK sequence, from N-terminal region to C-terminal region. Positive and negative values along *y*-axis indicate reduced or increased HDX, respectively, following CysK binding to CysE. Values at 0.03 and 1.67 min correspond to the mean of three replicates, whereas values at 16.67, 60, and 720 min are single measurements. The *dotted line*, plotted at ±0.46 D, indicates the threshold value for significant differences in HDX (99% confidence interval, calculated from the pooled SDs for all time points performed in replicates, at both states). Examples of peptides belonging to significant regions of CysK sequence (in terms of different behavior between the protein alone or in CS complex), discussed in the main text, are indicated above the chart. CS, cysteine synthase; HDX, hydrogen/deuterium exchange.

**Fig. 3:**
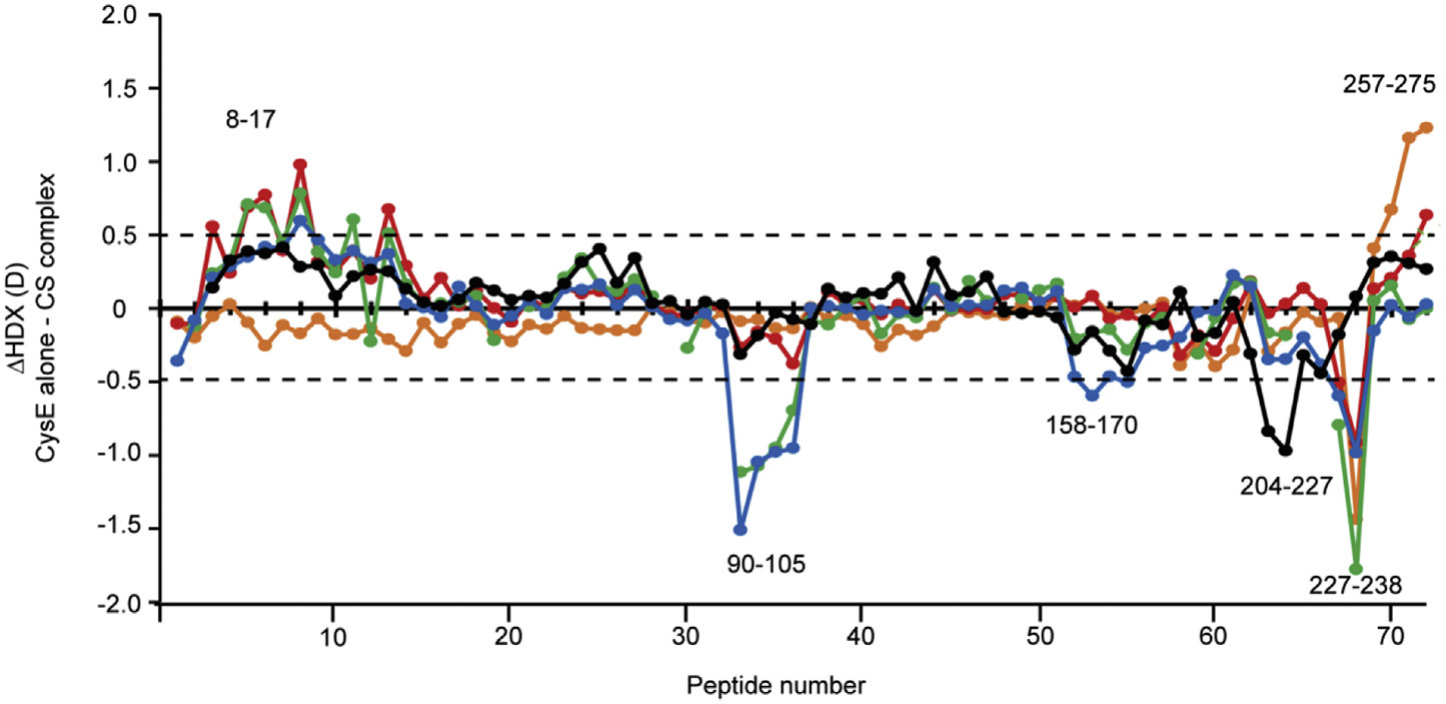
**HDX of free and complex-bound CysE.** Difference plot of the deuterium uptake (ΔHDX) between two states: CysE alone and CysE assembled with CysK (CS complex) for the 72 identified peptides at the five measured time points (*orange*—0.03 min; *red*—1.67 min; *green*—16.67 min; *cyan*—60 min; and *black*—720 min). The peptides are arranged along the *x*-axis according to their position in CysE sequence, from N-terminal to C-terminal region. Positive and negative values along *y*-axis indicate reduced or increased HDX, respectively, following CysE binding to CysK. Values at 0.03 and 1.67 min correspond to the mean of three replicates, whereas values at 16.67, 60, and 720 min are single measurements. The *dotted line*, plotted at ±0.49 D, indicates the threshold value for significant differences in HDX (99% confidence interval, calculated from the pooled SDs for all time points performed in replicates, at both states). Examples of peptides belonging to significant regions of CysE sequence, discussed in the main text, are indicated in the chart. CS, cysteine synthase; HDX, hydrogen/deuterium exchange.

To determine significant differences in HDX comparing two states, the SD of time points performed at least in triplicate was used to calculate a confidence interval (CI). The SD of individual peptides (n = 3) was averaged, and a pooled (root-mean-square) SD (*SD*_*pool*_) was calculated, using the equation:

(2) $$SD_{pool}=\sqrt{SD_{A}^{2}+SD_{B}^{2}}$$

where *SD*_*A*_ and *SD*_*B*_ are the states being compared (CysK alone and CysK within the CS complex or CysE alone and CysE within the CS complex). The *SD*_*pool*_ was then used to calculate the 99% CI, using the equation:

(3) $$CI=\pm \frac{\left(t_{n-1}\cdot SD_{pool}\right)}{\sqrt{n}}$$

where *t*_*n*−1_ is the table value for the two-tailed 99% CI with two degrees of freedom (*t*_*99*%_, _*n* = 3_ = 9.925) and n is the number of replicates (n = 3). The 99% CI, which was calculated as a threshold for significance of changes in dynamics, corresponded to 0.46 D for CysK and 0.49 D for CysE (*dotted line* in Figs. 2 and 3). The 99% CI was calculated from the pooled SDs for all time points performed in replicates, at both states, for CysK and CysE. In the comparative HDX analysis of the different states, a peptide was only considered to have a significant difference in HDX if it showed a significant difference in deuterium incorporation at a given time point, above the calculated CI.

To allow access to the HDX data of this study, the HDX data summary tables (supplemental Tables S1 and S2) and the HDX data tables (supplemental Tables S3 and S4) are included in the supplemental Data according to the community-based recommendations (39).

## Results and Discussion

In order to explore the dynamic properties of CysK and CysE upon forming the CS complex, we performed HDX-MS experiments where each protein component alone and in complex was labeled across a range of well-defined time points: 0.03, 1.67, 16.67, 60, and 720 min. The shortest time point (0.03 min or 2 s) was included to ensure maximal sampling of the HDX profile of each protein component alone and in complex (39).

Proteolysis with pepsin allowed the identification of 79 peptides covering 98.5% of CysK sequence (supplemental Fig. S1) and 72 peptides covering 97.8% of CysE sequence (supplemental Fig. S2), which were used to measure local HDX in each protein component as a function of time.

### CysK Dynamics Upon CS Complex Formation

**Fig. 4:**
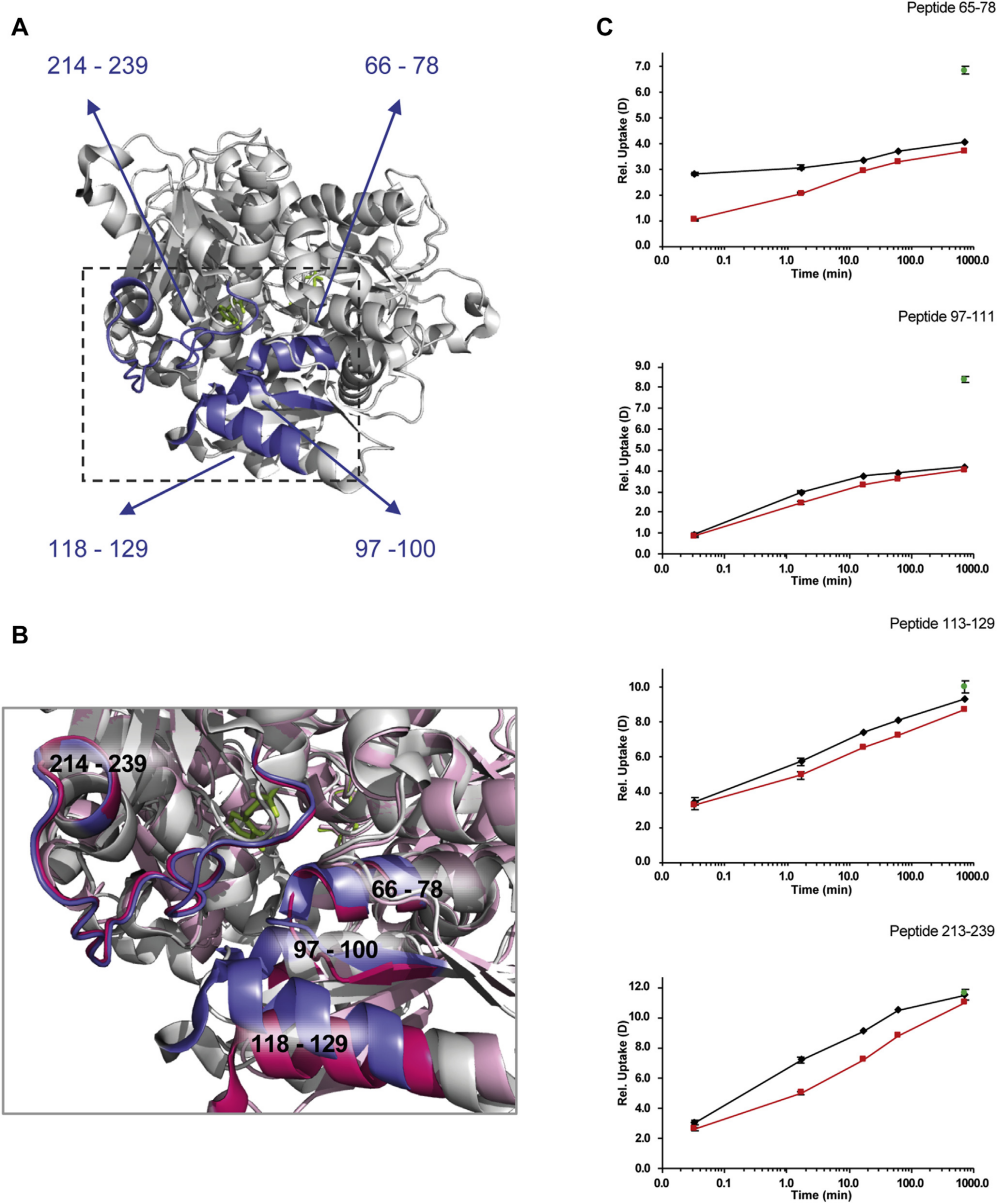
**Conformational dynamics of CysK alone and following CS complex formation.***A*, regions that display a significant decrease of HDX following CysK association with CysE (described in the main text) are colored in *blue* on the CysK structure (residues 66–78, residues 97–100, residues 118–129, and residues 214–239). CysK crystal structure corresponds to a “closed” conformation of the enzyme (Protein Data Bank code: 1D6S, colored in *light gray*). The pyridoxal 5'-phosphate (PLP) in the active site is shown in *light green stick* mode. *B*, close-up of CysK active site showing the details of the active-site entrance where conformational changes take place following complex formation. The closed structure is color coded as in panel *A*. The open structure (Protein Data Bank code: 1OAS) is shown in *light pink*, and the regions that display a decrease in HDX are colored *dark pink*. *C*, the HDX plots of representative peptides of regions showing significant changes in CysK dynamics upon complex formation are shown, with free CysK (*black lines*), CysK in CS complex (*red lines*), and the maximum deuterated control measurement (*green dots*) indicated at the last time point for clarity. Error bars indicate SDs for the 0.03, 1.67 min (n = 3) time points, and the maximum deuterated control (n = 3). CS, cysteine synthase; HDX, hydrogen/deuterium exchange.

Multiple regions of CysK showed significant changes in HDX upon binding to CysE. An overall comparison of the HDX between the unbound and bound states of CysK is illustrated in Figures 2 and 4, whereas the measured HDX values for both states are summarized for each peptide as deuterium uptake plots in supplemental Fig. S5. In Figure 2, the deuterium uptake of complex-bound CysK is subtracted from the deuterium uptake of CysK alone, for each peptide and time point. Examples of peptides belonging to regions of the protein that undergo significant changes in dynamics upon binding CysE are labeled in Figure 2, and the regions are mapped onto the CysK crystal structure in Figure 4, *A* and *B* (PDB codes: 1D6S and 1OAS). Overall, it is noteworthy that all ΔHDX values that exceed the ±0.46 D significance threshold range show a positive value (*i.e.*, a decrease in HDX upon complex formation), an indication that local regions of CysK are significantly more dynamic when unbound than within the complex. Decreased HDX upon complex formation was observed for the region encompassing residues 66 to 78, located in proximity to the active site. Representative mass spectra for CysK peptide 65 to 77, covering this region, are shown in supplemental Fig. S1. The HDX of unbound and complex-bound CysK for a representative peptide of this region (65–78), plotted as a function of the labeling time (0.03–720 min), is also shown in Figure 4*C*. A difference in HDX was observed at the earliest measured time point (2 s) and extended to 1.67 min. Moreover, changes in dynamics located at that region of the protein are in very good agreement with previous site-directed mutagenesis experiments and functional studies (10, 40), supporting that the CysE C-terminal tail inserts into the CysK active site and makes several contacts with the surrounding residues, including Ser70, Asn72, and Thr73 in *Haemophilus influenzae* CysK (10) (which correspond to the same residues in *E. coli* CysK, analyzed in this work). Our HDX results thus support that this region is an important part of the binding interface with CysE within CS complex.

Regions 97 to 100 and 118 to 129 also showed reduced HDX following CS complex formation. These regions are placed spatially close to each other (Fig. 4*B*) and belong to a flexible subdomain of the N-terminal domain that undergoes major conformational changes upon substrate binding (4). HDX plots for representative peptides belonging to these regions are shown in Figure 4*C*. A significant difference in the first part of the sequence (97–100) is apparent at the time point of 1.67 min, whereas for the latter residues (118–129), the difference in HDX started at 1.67 min and extended to the longer time points. This finding is indeed relevant, since this region undergoes the largest conformational changes that accompany the open to close transition induced by ligand binding to the protein, as demonstrated on *Salmonella* Typhimurium CysK (4), and we have previously proposed that this same rearrangement also takes place in CysK following interaction with CysE (35). Support for segmental movements in CysK upon transition between the “open” and “closed” state can be observed in Figure 4*C*, where regions showing significant differences in HDX are highlighted. Our HDX data show that when CysE binds to CysK active site, this flexible subdomain is stabilized. Intriguingly, our recent findings from a combined small-angle X-ray scattering (SAXS)-protein painting study showed that two lysine residues (K87 and K102) belonging to the flexible subdomain exhibit different exposure to the solvent upon CS complex assembly (15). These observations further corroborate the hypothesis that a stabilization of the closed conformation takes place following CS complex formation.

In the crystal structure of HiCysK in complex with CysE C-terminal tetrapeptide (PDB code: 1Y7L), the residue Gln143 was found to be involved in hydrogen bond formation with CysE C-terminal Ile267. Indeed, a decreased HDX indicative of a stabilization of this region was observed in peptide 142 to 154 and neighboring residues (see the region between peptides 35 and 40 in Fig. 2). However, we decided to use a conservative 99% CI as a threshold, to be more selective with respect to significance of variations in CysK dynamics; nonetheless if a less stringent 95% CI had been chosen, the changes in HDX in peptide 142 to 154 and overlapping would have exceeded that threshold.

Decreased HDX also occurred starting from the end of helix 10 (residues 214–216), encompassing the β-loop up to residue 239. This HDX decrease was observed at all the time points and most pronounced for time points 1.67, 16.67, and 60 min. The HDX of a representative peptide of this region is plotted as a function of labeling time (0.03–720 min) in Figure 4*C*. Residues 214 to 239 belong to the loop β8A–β9A, a conserved part of CysK sequence, located nearby the CysK active site. This result correlates well with our previous observations where a lysine residue (K226) was spotted as part of the interaction surface between CysK and CysE upon complex formation (15). Furthermore, previous findings (41) indicated that three residues located in this part of CysK sequence are crucial for its interaction with CysE: mutation of K217, H221, and K222 in *Arabidopsis thaliana* (corresponding to K221, H225, and K226 in *E. coli*), indeed, disrupts CS complex.

Reduced HDX indicative of structural stabilization upon CysK assembly with CysE could also be noticed in the C-terminal region of CysK, starting from residue 300 (around peptide number 70 in Fig. 2). This effect could be attributed to the movement toward the closed state of the protein. Indeed, it has been demonstrated that residues belonging to the C-terminal domain of CysK become more ordered in the closed structure (19). Peptides in this region showed differences in HDX that were borderline with respect to the general conservative CI used for the analysis. Notably, individual Student's *t* tests of the time points for which replicate data were obtained showed that the changes in HDX at the 1.67 min time point were significant for several overlapping peptides in this region.

### CysE Dynamics Following Binding to CysK

**Fig. 5:**
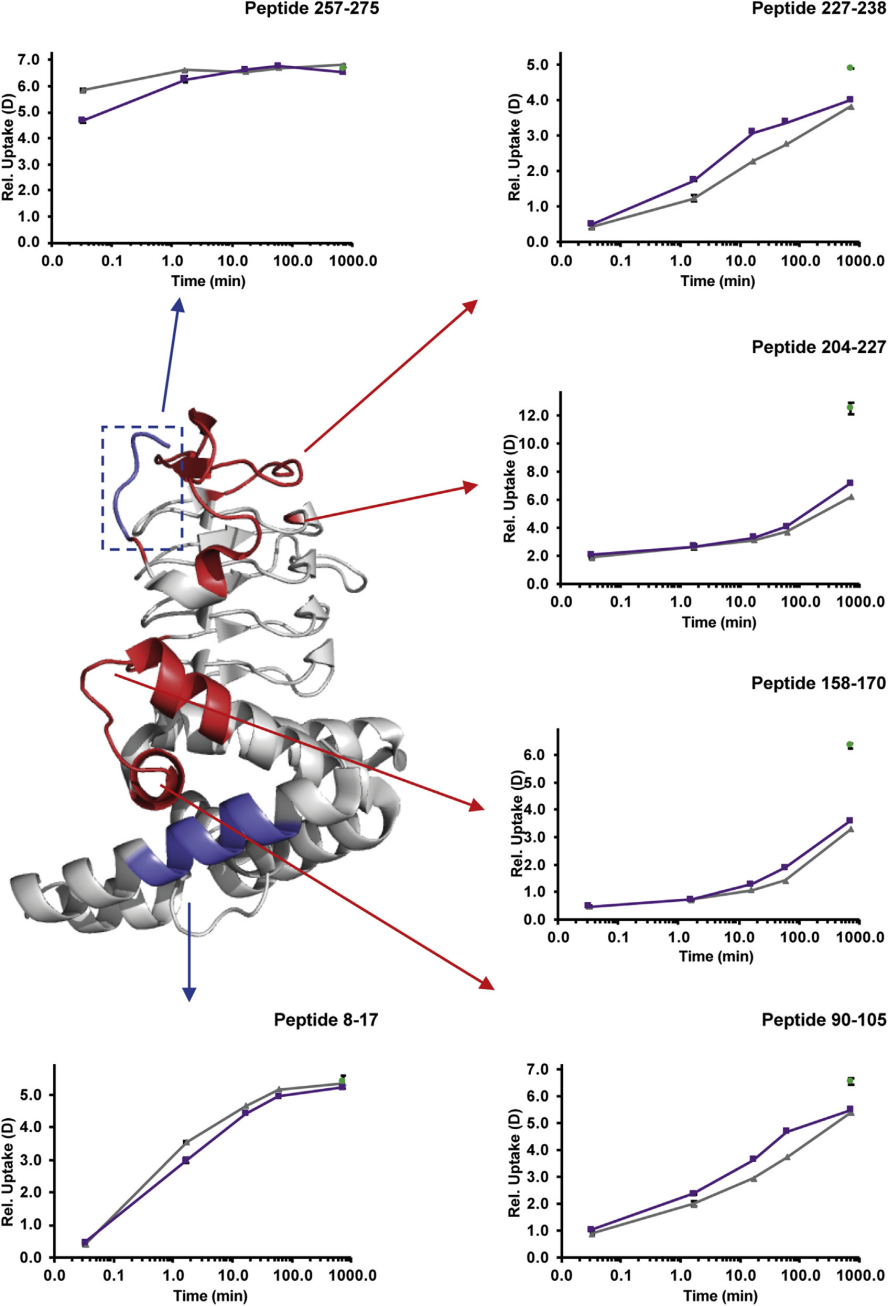
**Conformational dynamics of CysE alone and following CS complex formation.** Significant differences in HDX upon CS assembly are mapped onto a crystal structure of the CysE monomer (Protein Data Bank code: 1T3D). About 11 amino acids of CysE C-terminal sequence are missing in the deposited structure but were present in the construct used for HDX-MS measurements. The monomer has the same orientation as the boxed one in the structure of the whole CysE hexamer reported for Figure 6. Regions colored in *blue* showed a decrease in HDX (structural stabilization), whereas regions depicted in *red* underwent an increase in HDX (structural destabilization). Regions colored in *light gray* indicate insignificant difference in HDX between the two states compared or regions that were not covered by peptides. The HDX plots of representative peptides of regions showing significant changes in CysE dynamics upon complex formation are shown, with free CysE (*gray lines*), CysE in CS complex (*purple lines*), and the maximum deuterated control measurement (*green dots*) indicated at the last time point for clarity. Error bars indicate SDs for the 0.03, 1.67 min (n = 3) time points, and the maximum deuterated control (n = 3). CS, cysteine synthase; HDX, hydrogen/deuterium exchange.

**Fig. 6:**
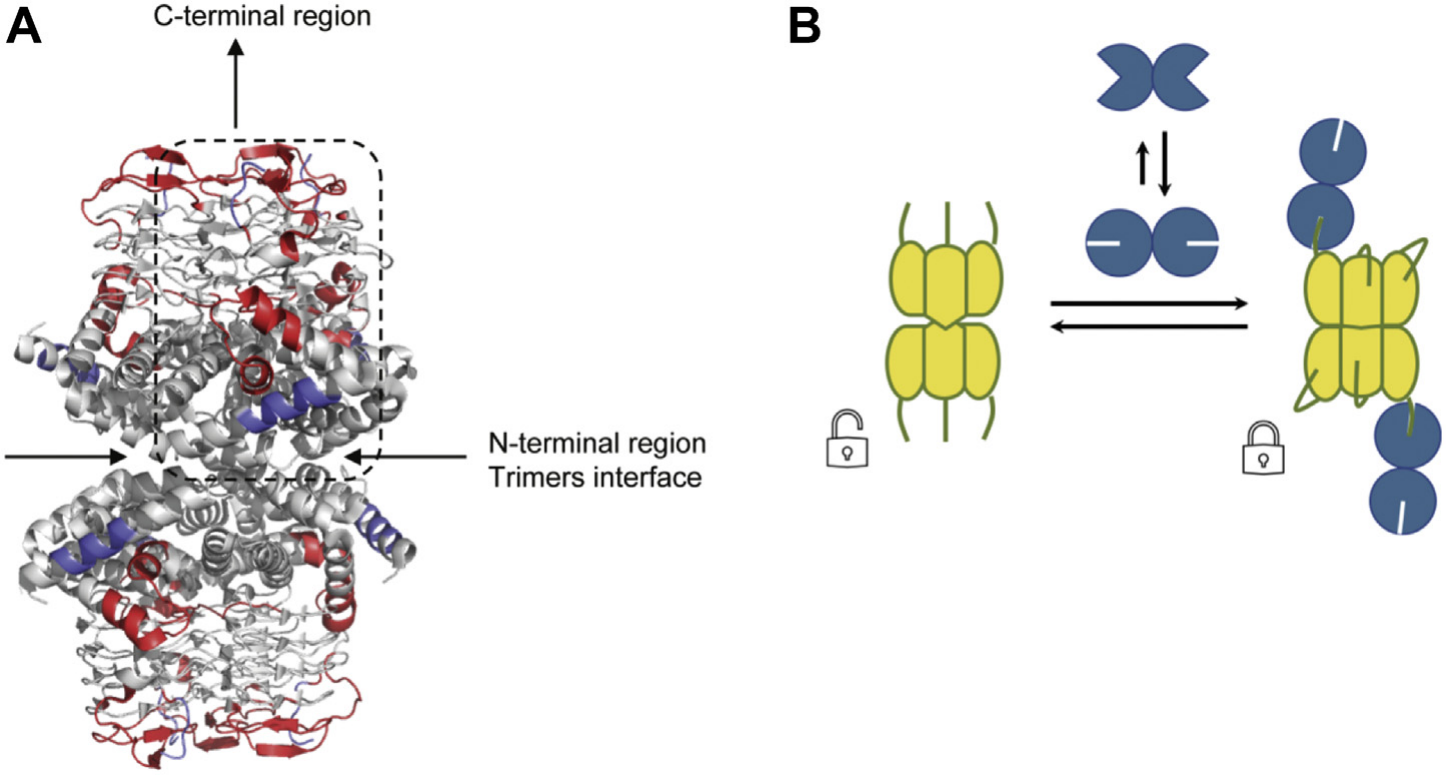
**Conformational dynamics of the CysE hexamer upon CS complex formation and proposed model for interaction with CysK.***A*, significant differences in HDX upon CS assembly are mapped onto a crystal structure of the CysE hexamer with the top-to-bottom mirror axis of the two trimers forming the hexameric structure (Protein Data Bank code: 1T3D). CysE C-terminal flexible peptides were not modeled in the deposited structure. Regions colored in *blue* correspond to a decrease in HDX (structural stabilization), whereas regions colored in *red* correspond to an increase in HDX (structural destabilization). The boxed subunit is the one reported, with the same orientation, for Figure 5. *B*, cartoon model showing the proposed mode of interaction between CysE hexamer (*yellow*) and two CysK dimers (*blue*). Insertion of one CysE C-terminus (*lines*) into one CysK active site stabilizes a closed conformation in both subunits of the latter enzyme. Upon CS complex formation, intertrimer and intratrimer allosteric signaling occurs in CysE. Intratrimer signaling stabilizes a closed structure also in CysE, where the unbound C-termini fold back into the active site. Intertrimer communication determines a nonrandom geometry in binding of the two CysK dimers at the opposite ends of the complex, as observed in SAXS experiments (15). CS, cysteine synthase; HDX, hydrogen/deuterium exchange; SAXS, small-angle X-ray scattering.

The effects of CS complex formation on the conformation and dynamics of CysE were also investigated. The interaction with CysK significantly impacted the HDX of different regions of CysE as shown in the difference plot comparing the two states of the protein (alone or in complex) in Figure 3. Regions showing significant differences in HDX are depicted on available crystal structures of one monomer (Fig. 5) and on the hexamer (Fig. 6*A*). The measured HDX values for CysE alone and complex bound are summarized for each peptide as deuterium uptake plots in supplemental Fig. S6.

A decrease in HDX was induced in the C-terminal tail of CysE (residues 260–275) as well as in the N-terminal part of the protein (residues 9–17). Oppositely, a significant increase in HDX was noticed for four distinct segments of the protein, including residues 87 to 105, 159 to 163, 216, and 228 to 256.

The HDX of a representative peptide of the C-terminal tail of CysE (residues 260–275) plotted as a function of labeling time (*i.e.*, 0.03–720 min) is illustrated in Figure 5. The effect of decreased HDX was considered significant for the earliest time point, that is, 2 s, but not for the other exposure times to deuterium, highlighting the benefit in this study of sampling the very short time regime. Indeed, this region of the protein is thought to be unstructured and flexible and accordingly exhibits very fast HDX, reaching the experimental maximum deuterium uptake already after 2 s. These observations expand previous findings from structural and functional studies (10, 12, 14), demonstrating that the C-terminus of CysE specifically plays a critical role in stabilizing CS complex formation by binding to the CysK active site.

The N-terminal region of the protein (residues 9–17), located at the interface between the two trimers of the hexamer, also showed reduced HDX upon CysE interaction with CysK. The effect started to appear at 1.67 min, extending also to the time point 16.67 min, as it can be observed in the HDX as a function of labeling time (0.03–720 min) for the representative peptide 8 to 17, illustrated in Figure 5. The observed reduction in HDX in the CysE C-terminus because of binding of CysK thus appears to be a critical step in transmitting an allosteric signal to the other side of the CysE hexamer, to induce structural stabilization at the trimer interface. Our HDX results are supported by previous findings from SAXS measurements (15), where the S shape of CS complex obtained from the *ab initio* modeling suggested a functional connection between the two opposite sides of the CysE hexamer interacting with CysK.

Oppositely, an HDX increase upon CysE binding to CysK, that is, an increased structural flexibility, was noticed in different segments of the protein sequence, involving residues 87 to 105, 159 to 163, 216, and in the region 228 to 256 that extend toward the C-terminus. This effect generally started to appear at the longer time points studied, as it can be observed from the HDX plots reported in Figure 5 for representative peptides, covering these regions.

Based on the detection of these localized changes in the HDX of CysE, we propose a binding model in which a concerted reorganization of CysE three-dimensional structure occurs upon CysK binding (Fig. 6). Hallmarks of this reorganization are evidenced by the stabilization (*i.e.*, reduced HDX) in the trimer interface and the observed widespread destabilization (*i.e.*, increased HDX) of regions of the CysE hexamer placed in between the C-terminus and the trimer interface (regions 87–105, 159–163, 216, and 228–256).

The structural basis underlying the apparently nonrandom binding of the second CysK dimer to only one subunit of each CysE trimer in the observed 2:3 complex stoichiometry is not known. As already mentioned, all the C-termini of the unbound CysE hexamer appear to be highly disordered in available crystallographic structures. Docking of just one (or two) of three CysE termini per trimer inside the CysK active site would result in dynamic and or conformational heterogeneity, and we would thus expect peak broadening in mass spectra to be observed during the HDX time course (42). Since no appreciable peak broadening was evident for peptides of the CysE C and N-termini in the CS complex with respect to the CysE alone (representative peptides are shown in supplemental Figs. S3 and S4), an intriguing symmetry in protein dynamics of all CysE subunits appears to be preserved upon CS complex formation despite the uneven 2:3 stoichiometry. Our binding model (Fig. 6*B*) proposes that binding of CysK to one CysE subunit triggers an intratrimer allosteric communication leading the C-termini of CysE subunits not involved in binding to CysK (*via* insertion into the active site of CysK) to fold back into the CysE active site. In this manner, the C-terminal sequence of bound and unbound CysE subunits is largely similar in terms of conformational flexibility and HDX.

In good agreement, evidence that CysE is able to bind part of its C-terminal sequence in its internal active site comes from structural studies that showed how the C-terminal sequence 241 to 257 is able to fold back into the active site and occupy the acetyl-CoA binding pocket with residues 254 to 257 (25).

We propose that this relocation/stabilization of CysE C-termini within a trimer, through a communication pathway across CysE sequence, is communicated to the N-termini, which form the interface between the two trimers of the CysE hexamer. As a result, the intertrimer interface is stabilized upon CS complex formation. This allosteric reorganization is further transmitted to the C-termini of the second CysE trimer, explaining the observed symmetry in terms of HDX and the nonrandom orientation of the two CysK molecules bound at the opposite sites of the CysE hexamer, yielding the S-shaped CS complex indicated by SAXS measurements (15).

## Conclusions

Our HDX-MS findings for CysK are in good agreement with existing data (site-directed mutagenesis, activity, and fluorescence spectroscopy) on how CysK is impacted upon forming the CS complex. Several regions of CysK showed significant changes in HDX upon binding to CysE. Residues located next to CysK active site were confirmed to interact with the CysE C-terminal sequence. Peptides belonging to a flexible subdomain of the CysK N-terminus, that undergoes a conformational transition to a closed state upon binding of the substrate, appeared to be stabilized in the CS complex. The same effect was observed for a conserved loop of CysK, containing residues that are known to be crucial for complex formation.

As for CysE, our HDX-MS results are particularly relevant in light of the limited amount of data about CysE structure and dynamics within the CS complex. We observed HDX decrease upon complex formation in agreement with the reported interaction mode with CysK, encompassing the insertion of the CysE C-terminal region into CysK ligand-binding site. Complex formation also led to increased dynamics of many segments of the protein sequence, demonstrating significant structural reorganization. This spans all the way from the region interacting with CysK to the N-terminal part of CysE on the opposite side of the protein, where an allosteric stabilization at the interface between the two CysE trimers is observed.

By HDX-MS, we thus obtained a comprehensive view on how CS complex formation modulates the dynamics of CysK and CysE in solution. Our data allow us to propose a refined model for how the mature 2:3 CysK–CysE complex is formed through allosteric signaling and reorganization of CysE. Overall, our findings provide important new molecular insights into how cysteine biosynthesis is regulated in bacteria and may prove useful for the design of novel antibacterial drugs.

## Data Availability

The MS proteomics data files including processed DynamX files and an overview of the HDX-MS data (supplemental Tables S1–S4) have been deposited to the ProteomeXchange Consortium *via* the PRIDE (43) partner repository with the dataset identifier PXD025300.

## Supplemental data

This article contains supplemental data.

## Conflict of interest

The authors declare no competing interests.

## Supplemental Data

Supplemental Tables S1–S4

Supplmental Figures S1–S6
