# Supplementary material for: Revealing the Dynamic Allosteric Changes Required for Formation of the Cysteine Synthase Complex by Hydrogen-Deuterium Exchange MS
Source: Mol Cell Proteomics. 2021 May 19;20:100098. doi: 10.1016/j.mcpro.2021.100098 (PMC8253905; doi:10.1016/j.mcpro.2021.100098)
Supplement: Supplmental Figures S1–S6 [file mmc2.pdf]

SUPPLEMENTAL MATERIAL

Revealing the dynamic allosteric changes required for formation of the cysteine synthase complex by hydrogen-deuterium exchange mass spectrometry

Brenda Rosa[1], Eleanor R. Dickinson[2], Marialaura Marchetti[1], Barbara Campanini[3], Barbara Pioselli[4], Stefano Bettati[1,5,6,*], Kasper Dyrberg Rand[2,7,*]

1 Biopharmanet-TEC Interdepartmental Center, University di Parma, 43124 Parma, Italy

2 Protein Analysis Group, Department of Pharmacy, University of Copenhagen, 2100 Copenhagen O, Denmark

3 Department of Food and Drug, University of Parma, 43124 Parma, Italy

4 Chiesi Farmaceutici, R & D Department, 43122 Parma, Italy

5 Department of Medicine and Surgery, University of Parma, 43126 Parma, Italy

6 Institute of Biophysics, CNR, 56124 Pisa, Italy

7 Lead Contact

* Correspondence: stefano.bettati@unipr.it; kasper.rand@sund.ku.dk

HDX-MS analysis optimization for CysK and CysE. In order to study the HDX of CysK and CysE exhaustively, we optimized the sequence coverage of the monitored proteins (i.e. the percentage of coverage of the amino acid sequence by the proteolytic peptides generated during the HDX-MS workflow), together with the number of peptides identified and the redundancy of covered amino acids (i.e. the number of peptides covering the same part of the protein sequence). The effect of the quenching buffer with different additives or chaotropic agents was evaluated, in particular four conditions were tested: a) 300 mM phosphate buffer (pH 2.3); b) 300 mM phosphate buffer (pH 2.3), 6 M guanidinium chloride; c) 300 mM phosphate buffer (pH 2.3), 6 M urea; d) 2 M glycine buffer (pH 2.3).

The addition to the quench buffer of the chaotropic agent guanidinium chloride or glycine led to a reduction in the sequence coverage and number of peptides identified for both the proteins analysed, CysK and CysE, respectively.

The best results for both CysK and CysE were obtained with the quench buffer without any additives (300 mM phosphate, pH 2.3, or buffer A) and with the quench buffer added with the chaotropic agent urea (300 mM phosphate buffer, pH 2.3, 6 M urea, or buffer C). Buffer A yielded a number of 79 peptides covering the 98.5% of CysK sequence with 3.24 redundancy and a total of 72 peptides, covering 97.8 % of CysE sequence with 3.65 redundancy, whereas buffer C yielded a number of 63 peptides covering 98.5% of CysK sequence with 2.51 redundancy and 58 peptides, covering 99.6% of CysE sequence, with 2.91 redundancy. Buffer A was chosen as quench buffer for HDX-MS analysis, according to the highest sequence coverage, number of peptides identified and redundancy obtained for CysK and CysE.

Supplementary figures

A

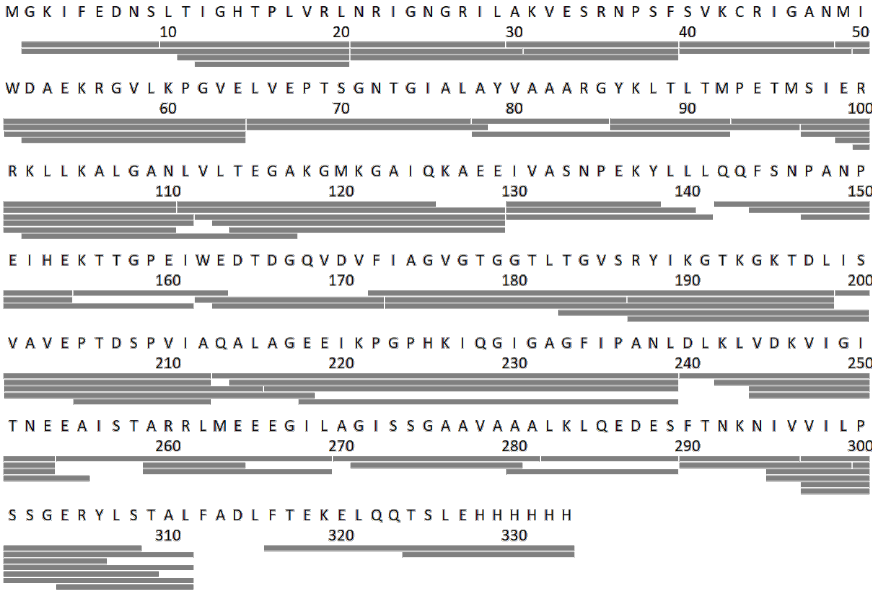

B

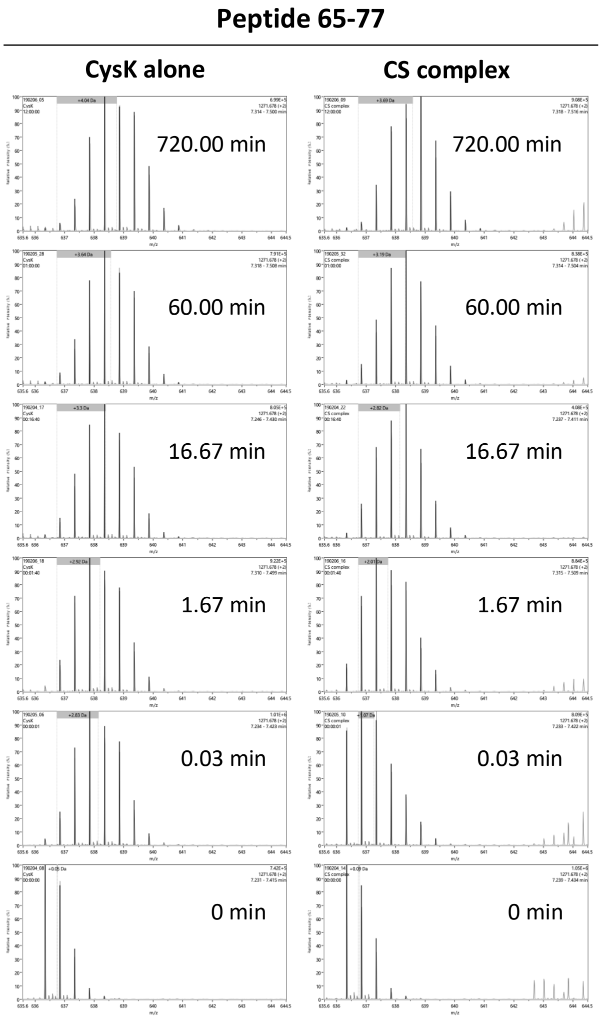

Figure S1. Sequence coverage and peptide representative mass spectra for HDX-MS analysis of CysK. Panel A. Digestion of the protein with immobilized pepsin yielded a total of 79 peptides. The peptides are depicted as grey bars and are aligned with the protein sequence. The coverage corresponds to 98.5%. **Panel B.** Representative mass spectra for CysK peptide 65-77 (charge state +2) in both states of the protein: alone (left) and assembled with CysE (CS complex, right). The isotopic distributions at 0 min time points correspond to the non-deuterated samples. Afterwards, the isotopic envelopes migrate to higher m/z values as a function of time (0.03 to 720 min), upon deuterium incorporation.

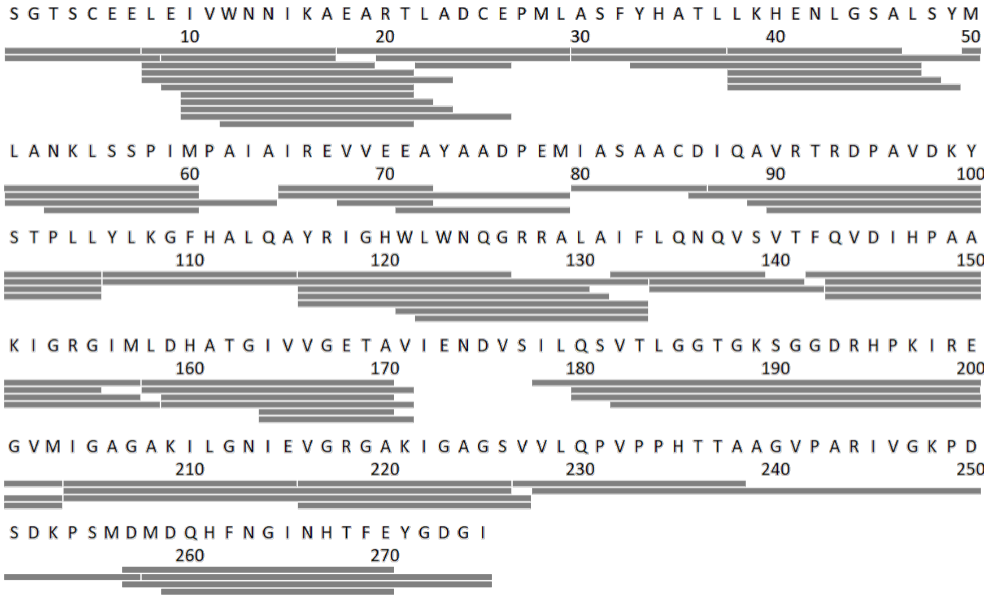

Figure S2. Sequence coverage for HDX-MS analysis of CysE. Online pepsin proteolysis identified a total of 72 peptides, covering 97.8 % of CysE sequence. The peptides, depicted as grey bars, are aligned with the protein sequence.

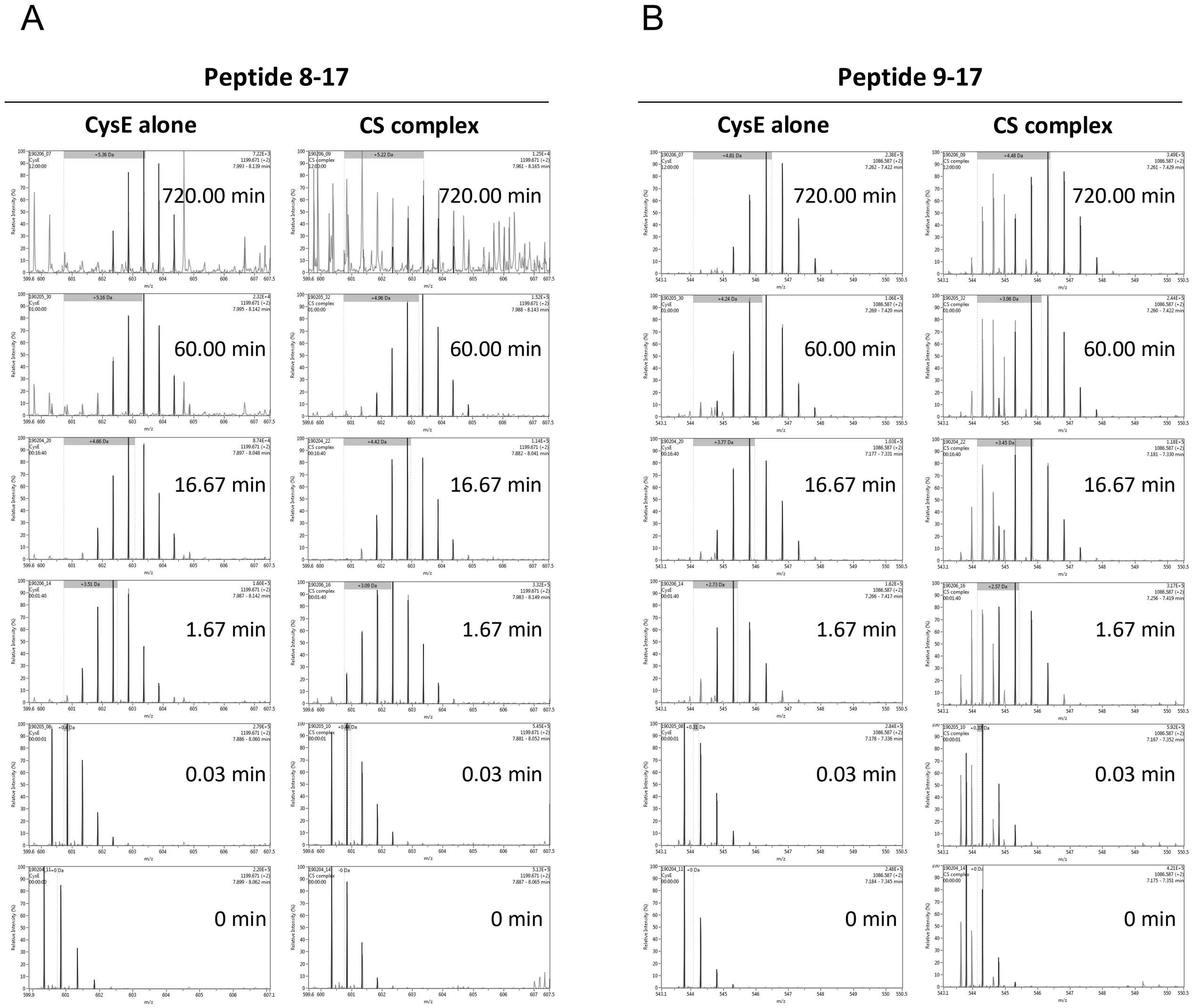

Figure S3. Representative mass spectra for CysE peptides, covering the N-terminal part of the sequence (peptides 8-17, 9-17). Representative mass spectra for the two states (CysK alone, left column, or in the CS complex, right column) at all sampled time points for peptides 8-17 (Panel A), 9-17 (Panel B). The charge state illustrated is +2 for all peptides. The isotopic distribution doesn't show appreciable broadening in CS complex spectra, compared to CysE alone.

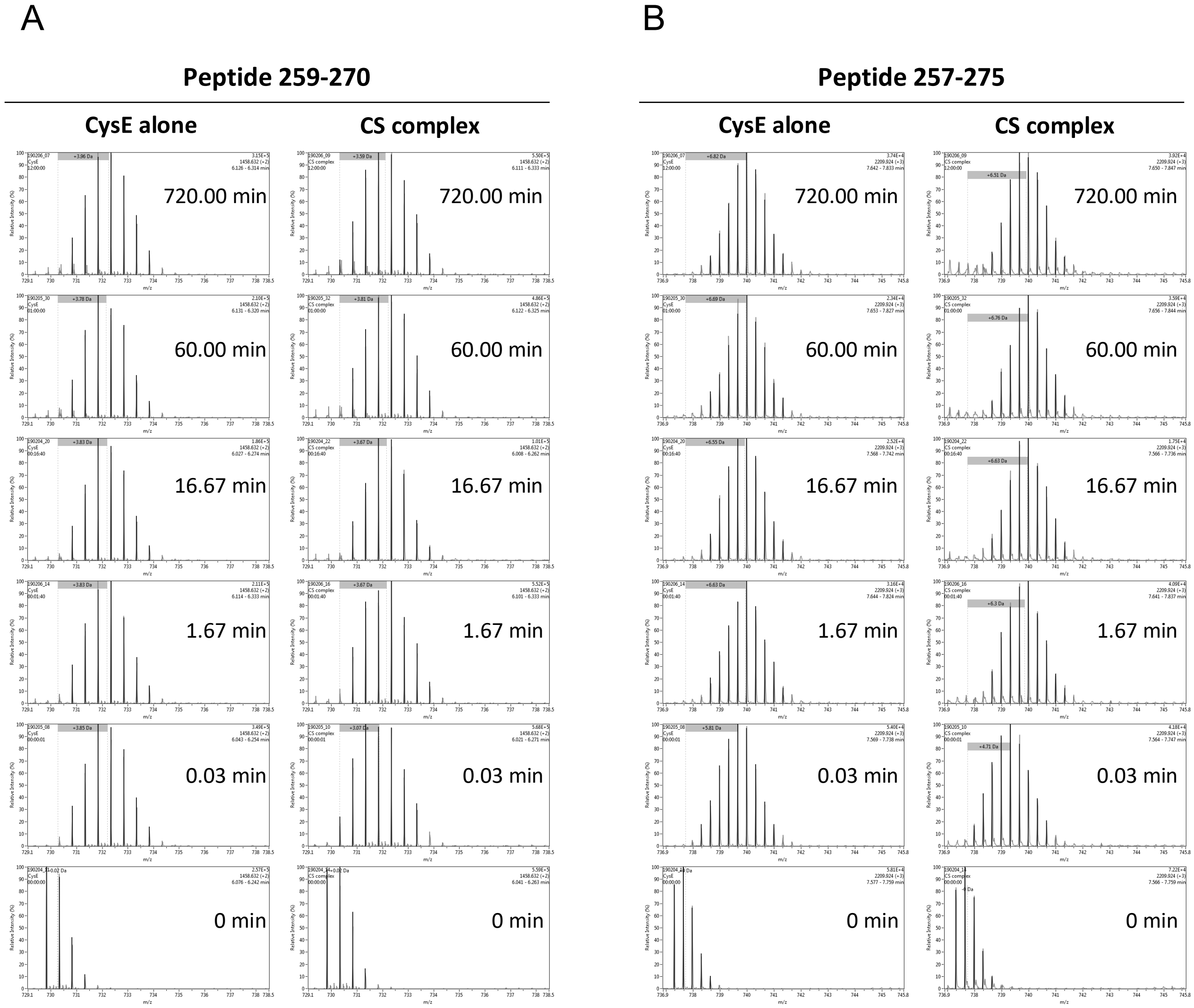

Figure S4. Representative mass spectra for CysE peptides, covering the C-terminal part of the sequence (peptides 259-270, 257-275). Representative mass spectra for the two states (CysK alone, left column, or in the CS complex, right column) at all sampled time points for peptides 259-270 (Panel A), and 257-275 (Panel B). The charge state is +2 (peptide 259-270) or +3 (peptide 257-275). No noticeable peak broadening is observed for CS complex spectra, compared to CysE alone.

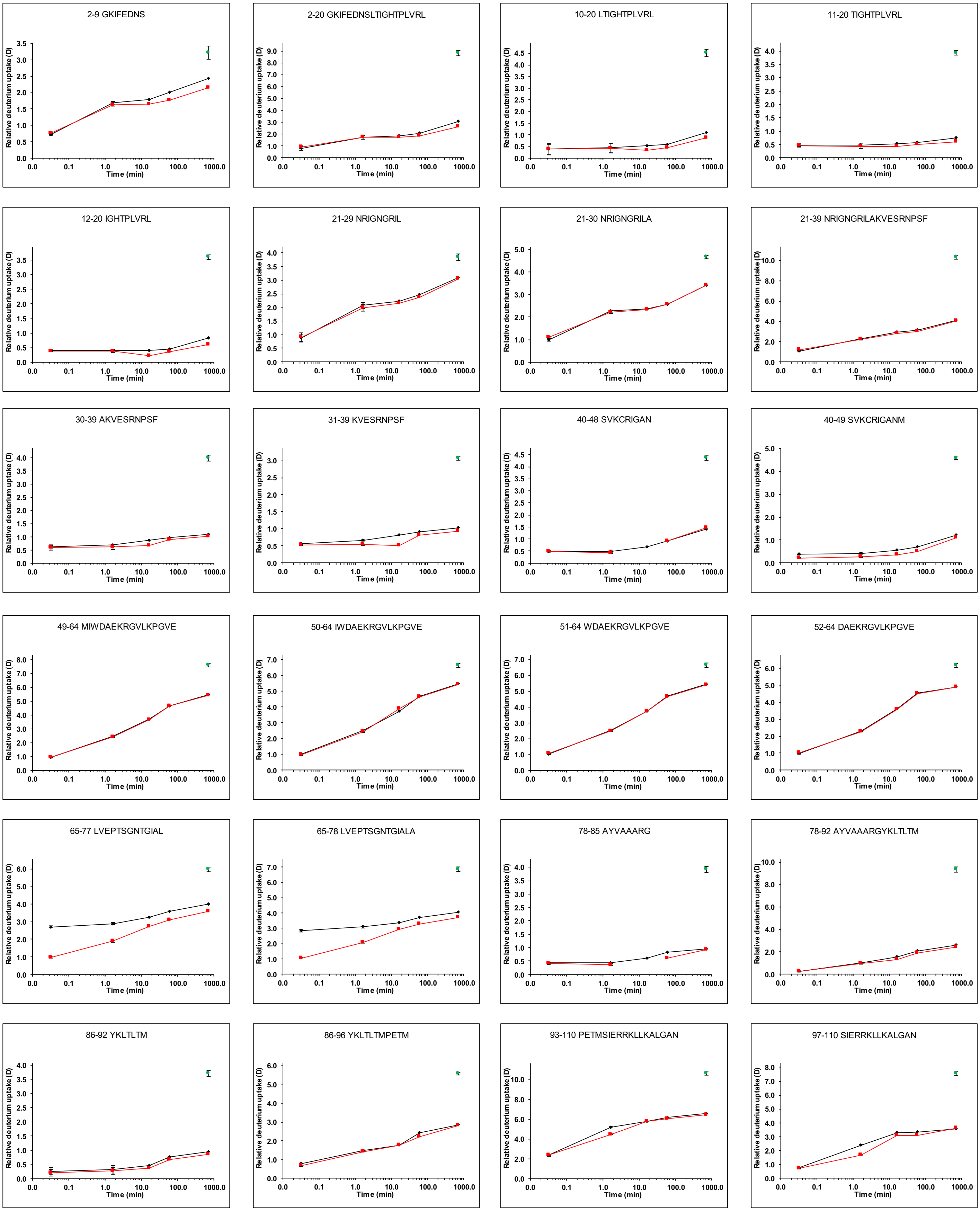
2-9 GKIFEDNS
2-20 GKIFEDNSLTIGHTPLVRL
10-20 LTIGHTPLVRL
11-20 TIGHTPLVRL
12-20 IGHTPLVRL
21-29 NRIGNGRIL
21-30 NRIGNGRILA
21-39 NRIGNGRILAKVESRNPSF
30-39 AKVESRNPSF
31-39 KVESRNPSF
40-48 SVKCRIGAN
40-49 SVKCRIGANM
49-64 MIWDAEKRGVLKPGVE
50-64 IWDAEKRGVLKPGVE
51-64 WDAEKRGVLKPGVE
52-64 DAEKRGVLKPGVE
65-77 LVEPTSGNTGIAL
65-78 LVEPTSGNTGIALA
78-85 AYVAAARG
78-92 AYVAAARGYKLTLTM
86-92 YKLTLTM
86-96 YKLTLTMPETM
93-110 PETMSIERRKLLKALGAN
97-110 SIERRKLLKALGAN
Relative deuterium uptake (D)
Time (min)

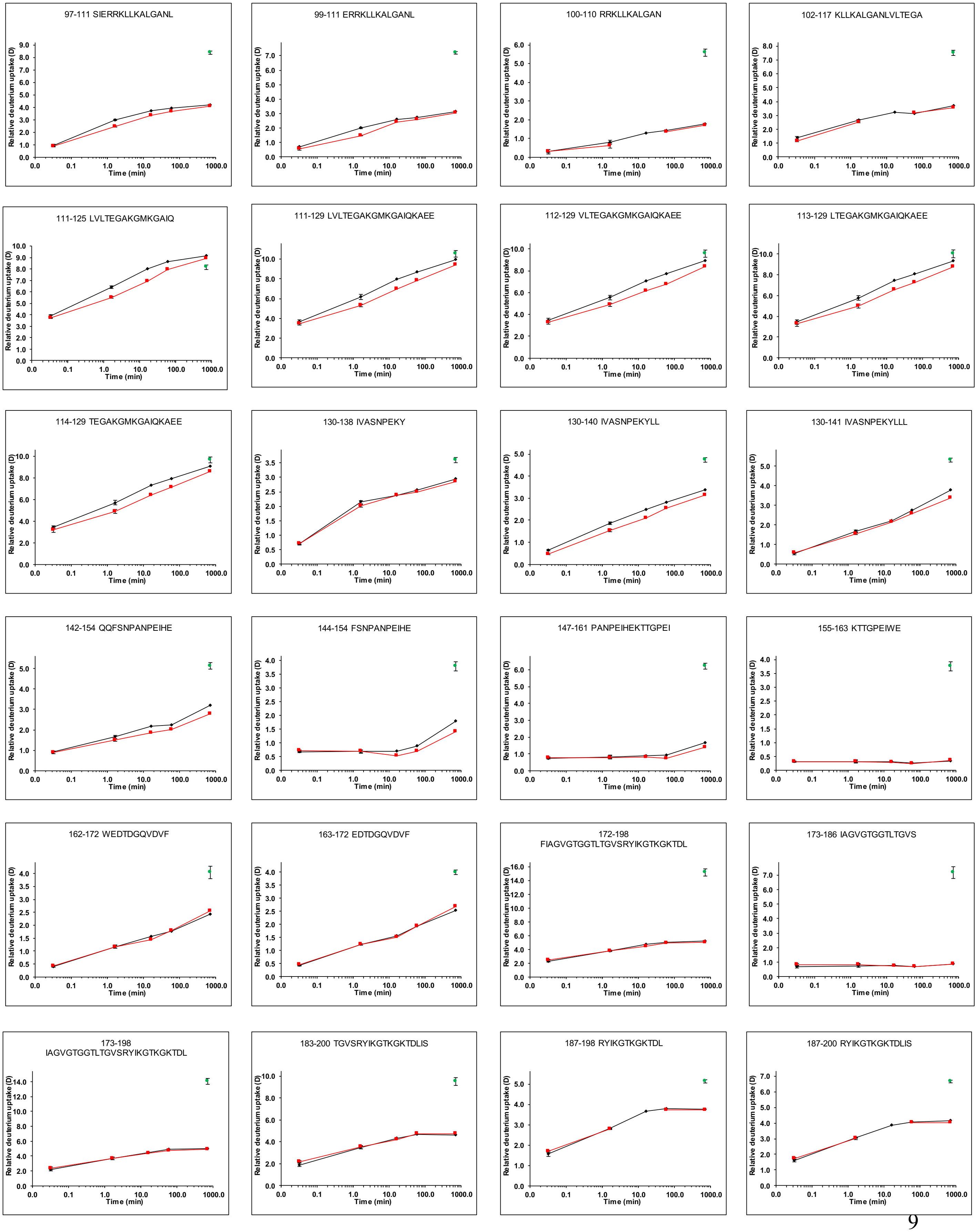

97-111 SIERRKLLKALGANL
99-111 ERRKLLKALGANL
100-110 RRKLLKALGAN
102-117 KLLKALGANLVLTEGA
111-125 LVLTEGAKGMKGAIQ
111-129 LVLTEGAKGMKGAIQKAEE
112-129 VLTEGAKGMKGAIQKAEE
113-129 LTEGAKGMKGAIQKAEE
114-129 TEGAKGMKGAIQKAEE
130-138 IVASNPEKY
130-140 IVASNPEKYLL
130-141 IVASNPEKYLLL
142-154 QQFSNPANPEIHE
144-154 FSNPANPEIHE
147-161 PANPEIHEKTTGPEI
155-163 KTTGPEIWE
162-172 WEDTDGQVDVF
163-172 EDTDGQVDVF
172-198 FIAGVGTGGTLTGVSRYIKGTKGKTDL
173-186 IAGVGTGGTLTGVS
173-198 IAGVGTGGTLTGVSRYIKGTKGKTDL
183-200 TGVSRYIKGTKGKTDLIS
187-198 RYIKGTKGKTDL
187-200 RYIKGTKGKTDLIS
Relative deuterium uptake (D)
Time (min)

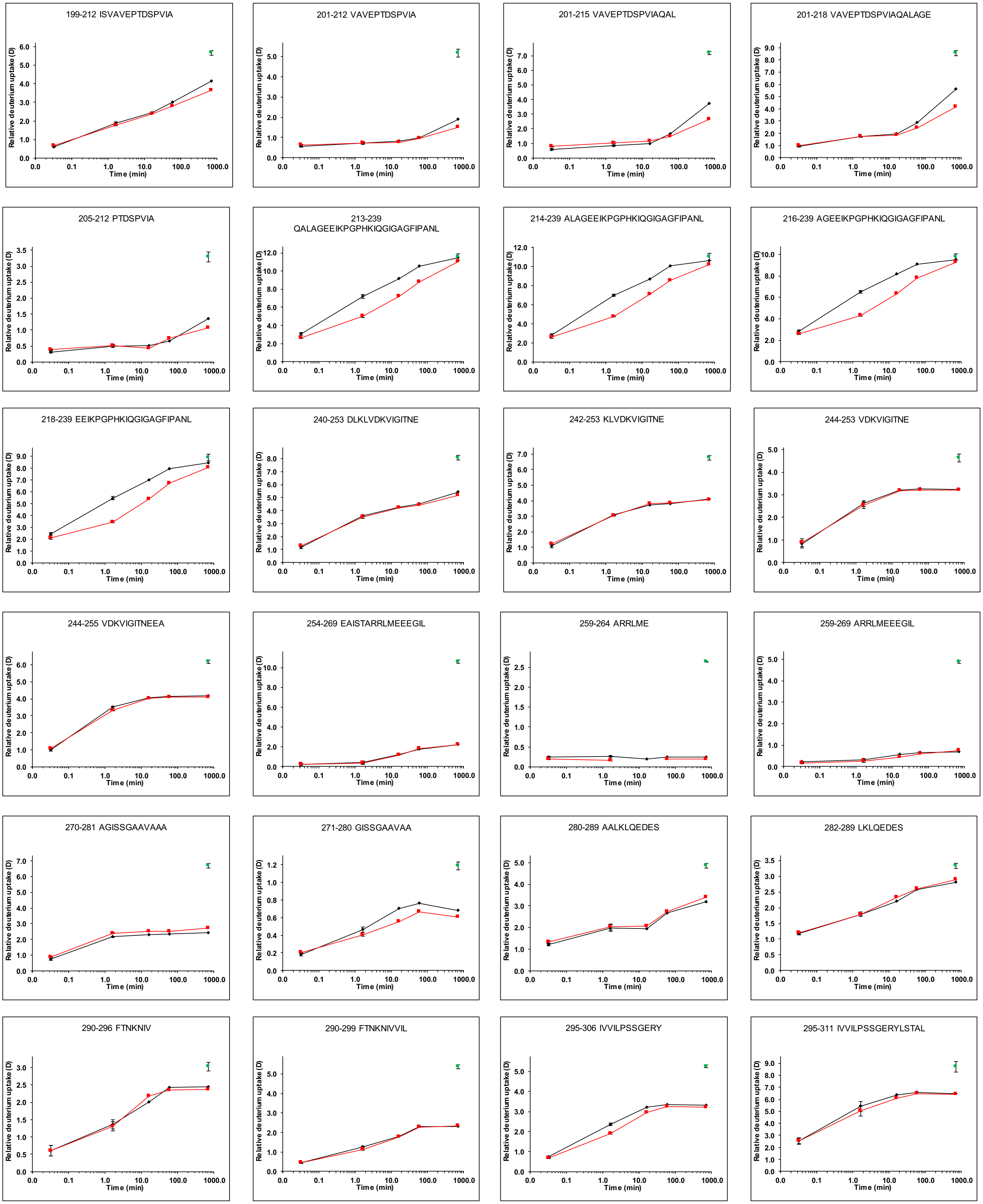
199-212 ISVAVEPTDSPVIA
201-212 VAVEPTDSPVIA
201-215 VAVEPTDSPVIAQAL
201-218 VAVEPTDSPVIAQALAGE
205-212 PTDSPVIA
213-239 QALAGEEIKPGPHKIQGIGAGFIPANL
214-239 ALAGEEIKPGPHKIQGIGAGFIPANL
216-239 AGEEIKPGPHKIQGIGAGFIPANL
218-239 EEIKPGPHKIQGIGAGFIPANL
240-253 DLKLVDKVIGITNE
242-253 KLVDKVIGITNE
244-253 VDKVIGITNE
244-255 VDKVIGITNEEA
254-269 EAISTARRLMEEEGIL
259-264 ARRLME
259-269 ARRLMEEEGIL
270-281 AGISSGAAVAAA
271-280 GISSGAAVAA
280-289 AALKLQEDES
282-289 LKLQEDES
290-296 FTNKNIV
290-299 FTNKNIVVIL
295-306 IVVILPSSGERY
295-311 IVVILPSSGERYLSTAL
Relative deuterium uptake (D)
Time (min)

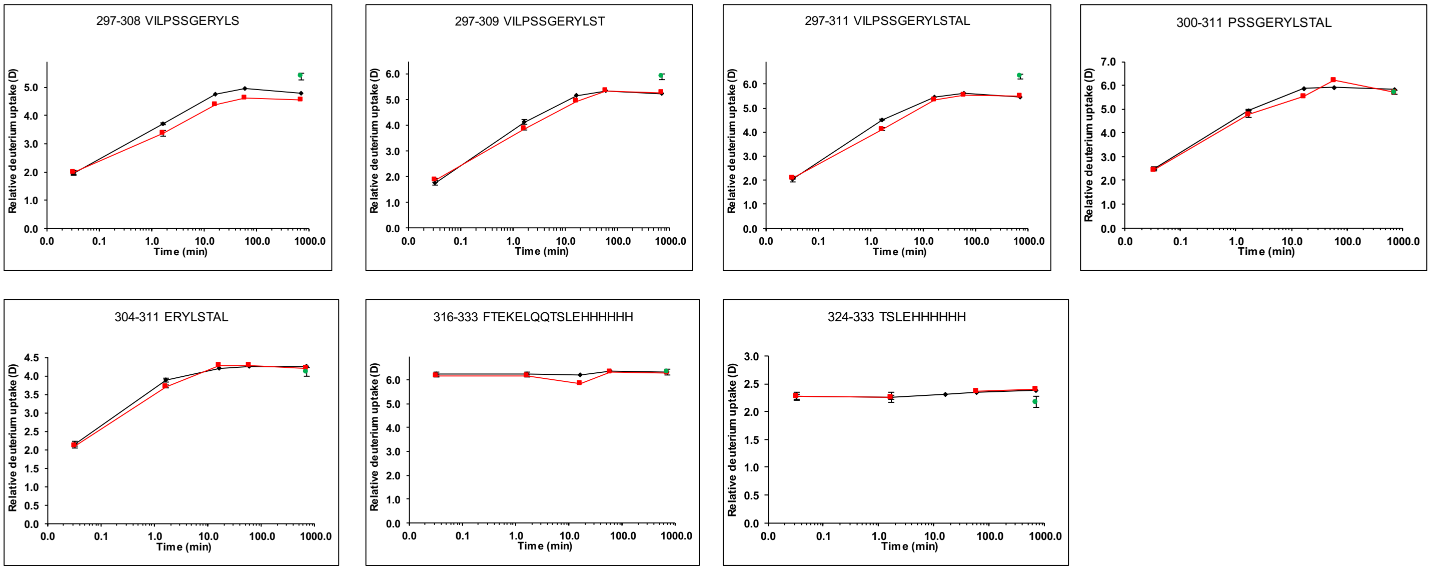

Figure S5. Deuterium uptake plots for HDX experiments of CysK in two states: free and complex-bound. The relative deuterium uptake is plotted as a function of labeling time (0.03 min, 1.67 min, 16.67 min, 60 min, 720 min) for all 79 identified peptides used in this experiment. Black and red lines represent the HDX of CysK free and in CS complex, respectively. Green dot at 720 min shows the HDX of maximum labeled sample. Standard deviations are plotted as error bars for the 0.03, 1.67 (n=3) time points and for the maximum deuterated control (n=3).

The time point 16.67 min is absent for peptides 40-48, 78-85, 187-198, 187-200, 259-264 and 324-333 for CysK in the CS complex, due to a low signal-to-noise ratio (S/N).

The time point 16.67 min is absent for peptides 100-110 and 102-117 for CysK in the CS complex due to overlapping peaks.

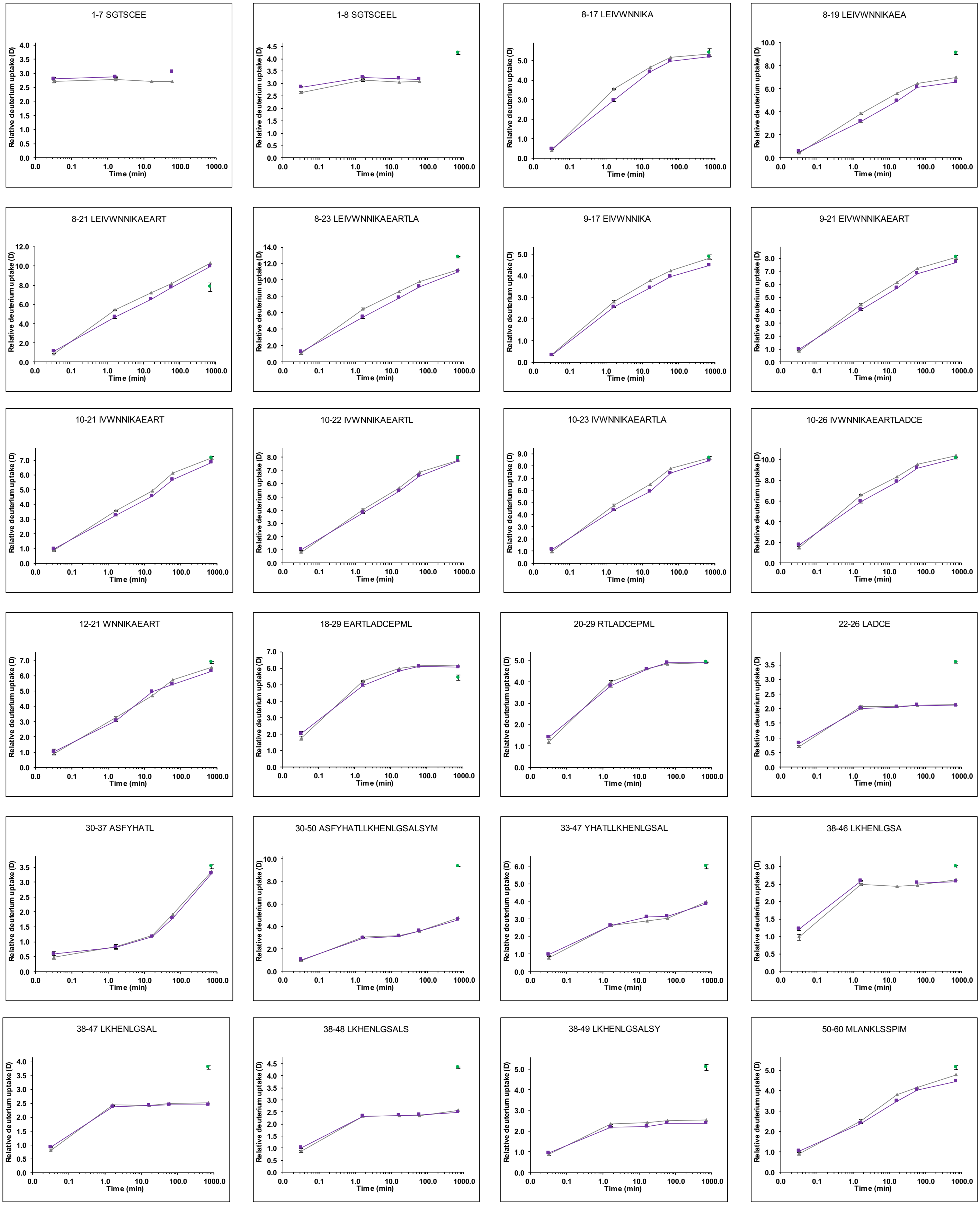
1-7 SGTSCEE
1-8 SGTSCEEL
8-17 LEIVWNNIKA
8-19 LEIVWNNIKAEA
8-21 LEIVWNNIKAEART
8-23 LEIVWNNIKAEARTLA
9-17 EIVWNNIKA
9-21 EIVWNNIKAEART
10-21 IVWNNIKAEART
10-22 IVWNNIKAEARTL
10-23 IVWNNIKAEARTLA
10-26 IVWNNIKAEARTLADCE
12-21 WNNIKAEART
18-29 EARTLADCEPML
20-29 RTLADCEPML
22-26 LADCE
30-37 ASFYHATL
30-50 ASFYHATLLKHENLGSALSYM
33-47 YHATLLKHENLGSAL
38-46 LKHENLGSA
38-47 LKHENLGSAL
38-48 LKHENLGSALS
38-49 LKHENLGSALSY
50-60 MLANKLSSPIM
Relative deuterium uptake (D)
Time (min)

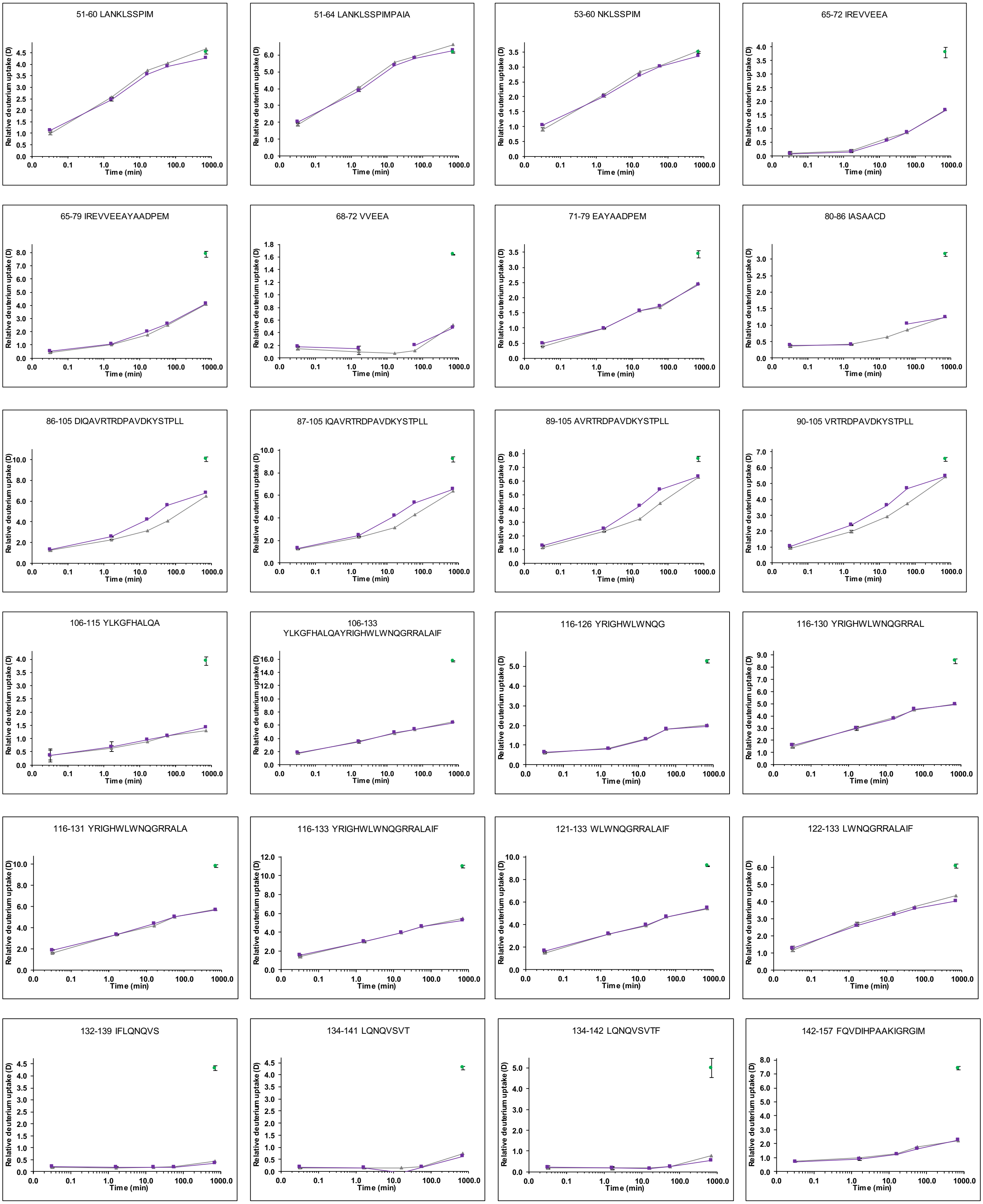
51-60 LANKLSSPIM
51-64 LANKLSSPIMPAIA
53-60 NKLSSPIM
65-72 IREVVEEA
65-79 IREVVEEAYAADPEM
68-72 VVEEA
71-79 EAYAADPEM
80-86 IASAACD
86-105 DIQAVRTRDPAVDKYSTPLL
87-105 IQAVRTRDPAVDKYSTPLL
89-105 AVRTRDPAVDKYSTPLL
90-105 VRTRDPAVDKYSTPLL
106-115 YLKGFHALQA
106-133 YLKGFHALQAYRIGHWLWNQGRRALAIF
116-126 YRIGHWLWNQG
116-130 YRIGHWLWNQGRRAL
116-131 YRIGHWLWNQGRRALA
116-133 YRIGHWLWNQGRRALAIF
121-133 WLWNQGRRALAIF
122-133 LWNQGRRALAIF
132-139 IFLQNQVS
134-141 LQNQVSVT
134-142 LQNQVSVTF
142-157 FQVDIHPAAKIGRGIM
Relative deuterium uptake (D)
Time (min)

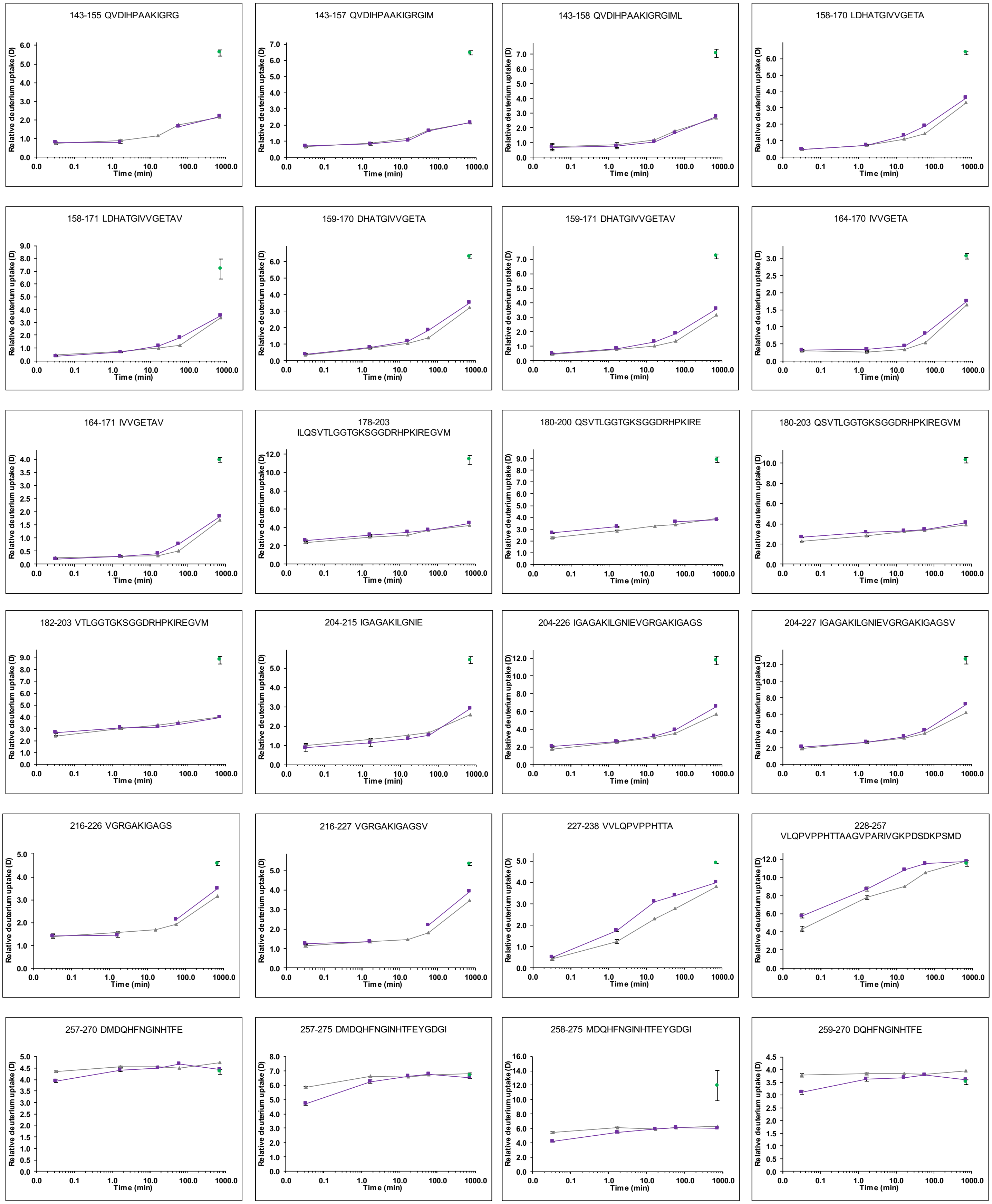
143-155 QVDIHPAAKIGRG
143-157 QVDIHPAAKIGRGIM
143-158 QVDIHPAAKIGRGIML
158-170 LDHATGIVVGETA
158-171 LDHATGIVVGETAV
159-170 DHATGIVVGETA
159-171 DHATGIVVGETAV
164-170 IVVGETA
164-171 IVVGETAV
178-203 ILQSVTLGGTGKSGGDRHPKIREGVM
180-200 QSVTLGGTGKSGGDRHPKIRE
180-203 QSVTLGGTGKSGGDRHPKIREGVM
182-203 VTLGGTGKSGGDRHPKIREGVM
204-215 IGAGAKILGNIE
204-226 IGAGAKILGNIEVGRGAKIGAGS
204-227 IGAGAKILGNIEVGRGAKIGAGSV
216-226 VGRGAKIGAGS
216-227 VGRGAKIGAGSV
227-238 VVLQPVPPHTTA
228-257 VLQPVPPHTTAAGVPARIVGKPDSDKPSMD
257-270 DMDQHFNGINHTFE
257-275 DMDQHFNGINHTFEYGDGI
258-275 MDQHFNGINHTFEYGDGI
259-270 DQHFNGINHTFE
Relative deuterium uptake (D)
Time (min)

Figure S6. Deuterium uptake plots for HDX experiments of CysE in two states: free and complex-bound. The relative deuterium uptake is plotted as a function of labeling time (0.03 min, 1.67 min, 16.67 min, 60 min, 720 min) for all 72 identified peptides used in this experiment. Grey and purple lines represent the HDX of CysE free and in CS complex, respectively. Green dot at 720 min shows the HDX of maximum labeled sample. Standard deviations are plotted as error bars for the 0.03, 1.67 (n=3) time points and for the maximum deuterated control (n=3).

The time point 16.67 min is absent for peptides 38-46, 68-72, 80-86, 143-155, 180-200, 216-226 and 216-227 for CysE in the CS complex, due to a low signal-to-noise ratio (S/N).

The absence, for peptide 1-7, of the time point 16.67 min for CysE in CS complex, and of the time point 720 min for CysE free and in the CS complex and the maximum labelled sample, is due to a low signal-to-noise ratio (S/N).

The absence of the time point 720 min for peptide 1-8 for CysE both unbound and complexed with CysK is due to the presence of overlapping peaks.

Supplementary tables

(Full-scale Excel versions of all Supplementary Tables are provided separately).

Supplementary Table 1. HDX DATA Summary table for CysK (see Supplementary Dataset 1 for full-scale excel version).

Data Set	CysK alone	CysK CS complex
HDX reaction details	Buffer: PBS, pH 7.45.<br>Percent deuterium: 89.1%. pH_{read}= 7.45. Temperature: 25 °C.	
HDX time course	0.03 min, 1.67 min, 16.67 min, 60 min, 720 min	
HDX control samples	Maximally-labeled control (CS complex)	
Back-exchange	Average - 39.2; Maximum - 57.47%; Minimun - 20.36%.	
Number of Peptides	79 peptides	
Sequence coverage	98.5% of the sequence	
Average peptide length / Redundancy	Average peptide lenght: 13.47 amino acids<br>Redundancy: 3.24	
Replicates (biological or technical)	0.03 min: $n_{biological}$ = 1, $n_{technical}$ = 3<br>1.67 min: $n_{biological}$ = 1, $n_{technical}$ = 3<br>16.67 min: $n_{biological}$ = 1, $n_{technical}$ = 1<br>60 min: $n_{biological}$ = 1, $n_{technical}$ = 1<br>720 min: $n_{biological}$ = 1, $n_{technical}$ = 1	0.03 min: $n_{biological}$ = 1, $n_{technical}$ = 3<br>1.67 min: $n_{biological}$ = 1, $n_{technical}$ = 3<br>16.67 min: $n_{biological}$ = 1, $n_{technical}$ = 1<br>60 min: $n_{biological}$ = 1, $n_{technical}$ = 1<br>720 min: $n_{biological}$ = 1, $n_{technical}$ = 1<br>Maximally-labeled control: $n_{biological}$ = 1, $n_{technical}$ = 3
Significant differences in HDX (delta HDX > X D)	The difference in HDX had to be higher than the calculated 99% confidence interval (see methods for additional criteria).	
99% confidence interval	0.46 D	

Supplementary Table 2. HDX DATA Summary table for CysE (see Supplementary Dataset 2 for full-scale excel version).

Data Set	CysE alone	CysE CS complex
HDX reaction details	Buffer: PBS, pH 7.45.<br>Percent deuterium: 89.1%. pH_{read}= 7.45. Temperature: 25 °C.	
HDX time course	0.03 min, 1.67 min, 16.67 min, 60 min, 720 min	
HDX control samples	Maximally-labeled control (CS complex)	
Back-exchange (average)	Average - 37.47; Maximum - 63.15%; Minimun - 3.70%.	
Number of Peptides	72 peptides	
Sequence coverage	97.8% of the sequence	
Average peptide length / Redundancy	Average peptide lenght: 13.64 amino acids<br>Redundancy: 3.65	
Replicates (biological or technical)	0.03 min: $n_{biological}$ = 1, $n_{technical}$ = 3<br>1.67 min: $n_{biological}$ = 1, $n_{technical}$ = 3<br>16.67 min: $n_{biological}$ = 1, $n_{technical}$ = 1<br>60 min: $n_{biological}$ = 1, $n_{technical}$ = 1<br>720 min: $n_{biological}$ = 1, $n_{technical}$ = 1	0.03 min: $n_{biological}$ = 1, $n_{technical}$ = 3<br>1.67 min: $n_{biological}$ = 1, $n_{technical}$ = 3<br>16.67 min: $n_{biological}$ = 1, $n_{technical}$ = 1<br>60 min: $n_{biological}$ = 1, $n_{technical}$ = 1<br>720 min: $n_{biological}$ = 1, $n_{technical}$ = 1<br>Maximally-labeled control: $n_{biological}$ = 1, $n_{technical}$ = 3
Significant differences in HDX (delta HDX > X D)	The difference in HDX had to be higher than the calculated 99% confidence interval (see methods for additional criteria).	
99% confidence interval	0.49 D	

Supplementary Table 3. HDX DATA TABLE CysK. DATA OUTPUT FOR KINETICS EXPERIMENTS (see Supplementary Dataset 3 for full-scale excel version).

Protein state	Start	End	Sequence	Peptide mass (Da)	Retention time (min)	HDX time (min)	Uptake (D)	Uptake SD (D)
CS complex	2	9	GKIFEDNS	909.424	5.178563	0	0	0
CS complex	2	9	GKIFEDNS	909.424	5.209345	0.033	0.766192	0.040915
CS complex	2	9	GKIFEDNS	909.424	5.233234	1.667	1.627715	0.050115
CS complex	2	9	GKIFEDNS	909.424	5.165497	16.667002	1.646587	0.040716
CS complex	2	9	GKIFEDNS	909.424	5.242655	60.000004	1.775636	0.051139
CS complex	2	9	GKIFEDNS	909.424	5.240862	720.000061	2.15167	0.049672
CysK	2	9	GKIFEDNS	909.424	5.174262	0	0	0
CysK	2	9	GKIFEDNS	909.424	5.183807	0.033	0.715451	0.044132
CysK	2	9	GKIFEDNS	909.424	5.233136	1.667	1.682827	0.0419
CysK	2	9	GKIFEDNS	909.424	5.193146	16.667002	1.780362	0.017552
CysK	2	9	GKIFEDNS	909.424	5.248806	60.000004	2.000845	0.056823
CysK	2	9	GKIFEDNS	909.424	5.239297	720.000061	2.43148	0.025959
maxD control	2	9	GKIFEDNS	909.424	5.178563	0	0	0
maxD control	2	9	GKIFEDNS	909.424	5.393363	720.000061	3.210448	0.20294
CS complex	2	20	GKIFEDNSLTIGHTPLVRL	2110.1582	7.568525	0	0	0
CS complex	2	20	GKIFEDNSLTIGHTPLVRL	2110.1582	7.605428	0.033	0.897746	0.13529
CS complex	2	20	GKIFEDNSLTIGHTPLVRL	2110.1582	7.646656	1.667	1.739861	0.158248
CS complex	2	20	GKIFEDNSLTIGHTPLVRL	2110.1582	7.557773	16.667002	1.715654	0.126406
CS complex	2	20	GKIFEDNSLTIGHTPLVRL	2110.1582	7.647536	60.000004	1.851034	0.132367
CS complex	2	20	GKIFEDNSLTIGHTPLVRL	2110.1582	7.649575	720.000061	2.622923	0.117472
CysK	2	20	GKIFEDNSLTIGHTPLVRL	2110.1582	7.563797	0	0	0
CysK	2	20	GKIFEDNSLTIGHTPLVRL	2110.1582	7.561042	0.033	0.792677	0.152968
CysK	2	20	GKIFEDNSLTIGHTPLVRL	2110.1582	7.645238	1.667	1.747484	0.160667
CysK	2	20	GKIFEDNSLTIGHTPLVRL	2110.1582	7.573985	16.667002	1.85555	0.141434
CysK	2	20	GKIFEDNSLTIGHTPLVRL	2110.1582	7.657516	60.000004	2.08294	0.140721
CysK	2	20	GKIFEDNSLTIGHTPLVRL	2110.1582	7.651052	720.000061	3.064085	0.131069
maxD control	2	20	GKIFEDNSLTIGHTPLVRL	2110.1582	7.568528	0	0	0
maxD control	2	20	GKIFEDNSLTIGHTPLVRL	2110.1582	7.791917	720.000061	8.812825	0.23345
CS complex	10	20	LTIGHTPLVRL	1219.7448	7.128202	0	0	0
CS complex	10	20	LTIGHTPLVRL	1219.7448	7.163112	0.033	0.376616	0.242116
CS complex	10	20	LTIGHTPLVRL	1219.7448	7.208971	1.667	0.420246	0.199844
CS complex	10	20	LTIGHTPLVRL	1219.7448	7.112821	16.667002	0.330771	0.132402
CS complex	10	20	LTIGHTPLVRL	1219.7448	7.210917	60.000004	0.435572	0.20657
CS complex	10	20	LTIGHTPLVRL	1219.7448	7.21175	720.000061	0.863617	0.200506
CysK	10	20	LTIGHTPLVRL	1219.7448	7.122874	0	0	0
CysK	10	20	LTIGHTPLVRL	1219.7448	7.114397	0.033	0.382822	0.207212

**CysK**	10	20	LTIGHTPLVRL	1219.7448	7.207945	1.667	0.437959	0.177836
**CysK**	10	20	LTIGHTPLVRL	1219.7448	7.129327	16.667002	0.524553	0.120362
**CysK**	10	20	LTIGHTPLVRL	1219.7448	7.216185	60.000004	0.580812	0.157166
**CysK**	10	20	LTIGHTPLVRL	1219.7448	7.212876	720.000061	1.1104	0.112944
**maxD control**	10	20	LTIGHTPLVRL	1219.7448	7.128202	0	0	0
**maxD control**	10	20	LTIGHTPLVRL	1219.7448	7.376148	720.000061	4.513692	0.151156
**CS complex**	11	20	TIGHTPLVRL	1106.6606	6.480489	0	0	0
**CS complex**	11	20	TIGHTPLVRL	1106.6606	6.522655	0.033	0.456582	0.061436
**CS complex**	11	20	TIGHTPLVRL	1106.6606	6.569976	1.667	0.417072	0.065751
**CS complex**	11	20	TIGHTPLVRL	1106.6606	6.477149	16.667002	0.4185	0.03349
**CS complex**	11	20	TIGHTPLVRL	1106.6606	6.570342	60.000004	0.496438	0.058198
**CS complex**	11	20	TIGHTPLVRL	1106.6606	6.573777	720.000061	0.602029	0.033153
**CysK**	11	20	TIGHTPLVRL	1106.6606	6.482314	0	0	0
**CysK**	11	20	TIGHTPLVRL	1106.6606	6.482062	0.033	0.47824	0.050436
**CysK**	11	20	TIGHTPLVRL	1106.6606	6.572208	1.667	0.472784	0.04549
**CysK**	11	20	TIGHTPLVRL	1106.6606	6.498293	16.667002	0.525684	0.046011
**CysK**	11	20	TIGHTPLVRL	1106.6606	6.580289	60.000004	0.572107	0.074429
**CysK**	11	20	TIGHTPLVRL	1106.6606	6.574941	720.000061	0.740136	0.036393
**maxD control**	11	20	TIGHTPLVRL	1106.6606	6.480489	0	0	0
**maxD control**	11	20	TIGHTPLVRL	1106.6606	6.765258	720.000061	3.922221	0.08754
**CS complex**	12	20	IGHTPLVRL	1005.613	7.125494	0	0	0
**CS complex**	12	20	IGHTPLVRL	1005.613	7.161563	0.033	0.376254	0.01194
**CS complex**	12	20	IGHTPLVRL	1005.613	7.208146	1.667	0.376373	0.051646
**CS complex**	12	20	IGHTPLVRL	1005.613	7.113173	16.667002	0.214415	0
**CS complex**	12	20	IGHTPLVRL	1005.613	7.209087	60.000004	0.352353	0
**CS complex**	12	20	IGHTPLVRL	1005.613	7.209243	720.000061	0.609707	0
**CysK**	12	20	IGHTPLVRL	1005.613	7.122191	0	0	0
**CysK**	12	20	IGHTPLVRL	1005.613	7.114616	0.033	0.40209	0.014125
**CysK**	12	20	IGHTPLVRL	1005.613	7.206348	1.667	0.410747	0.033732
**CysK**	12	20	IGHTPLVRL	1005.613	7.129412	16.667002	0.395183	1.14E-13
**CysK**	12	20	IGHTPLVRL	1005.613	7.216233	60.000004	0.455851	1.14E-13
**CysK**	12	20	IGHTPLVRL	1005.613	7.210332	720.000061	0.842736	1.14E-13
**maxD control**	12	20	IGHTPLVRL	1005.613	7.125494	0	0	0
**maxD control**	12	20	IGHTPLVRL	1005.613	7.375871	720.000061	3.610794	0.084724
**CS complex**	21	29	NRIGNGRIL	1012.5941	5.460505	0	0	0
**CS complex**	21	29	NRIGNGRIL	1012.5941	5.498527	0.033	0.909705	0.152086
**CS complex**	21	29	NRIGNGRIL	1012.5941	5.533659	1.667	1.98322	0.120174
**CS complex**	21	29	NRIGNGRIL	1012.5941	5.462545	16.667002	2.156947	0.12747
**CS complex**	21	29	NRIGNGRIL	1012.5941	5.538312	60.000004	2.37255	0.108859
**CS complex**	21	29	NRIGNGRIL	1012.5941	5.5418	720.000061	3.039746	0.110647
**CysK**	21	29	NRIGNGRIL	1012.5941	5.460238	0	0	0

**CysK**	21	29	NRIGNGRIL	1012.5941	5.464817	0.033	0.875042	0.140249
**CysK**	21	29	NRIGNGRIL	1012.5941	5.534619	1.667	2.071938	0.103325
**CysK**	21	29	NRIGNGRIL	1012.5941	5.479745	16.667002	2.227524	0.081456
**CysK**	21	29	NRIGNGRIL	1012.5941	5.552858	60.000004	2.466083	0.087715
**CysK**	21	29	NRIGNGRIL	1012.5941	5.542038	720.000061	3.106171	0.084736
**maxD control**	21	29	NRIGNGRIL	1012.5941	5.460505	0	0	0
**maxD control**	21	29	NRIGNGRIL	1012.5941	5.749998	720.000061	3.844521	0.116155
**CS complex**	21	30	NRIGNGRILA	1083.6312	5.128304	0	0	0
**CS complex**	21	30	NRIGNGRILA	1083.6312	5.161134	0.033	1.0989	0.021412
**CS complex**	21	30	NRIGNGRILA	1083.6312	5.188852	1.667	2.227305	0.081441
**CS complex**	21	30	NRIGNGRILA	1083.6312	5.12359	16.667002	2.33793	0
**CS complex**	21	30	NRIGNGRILA	1083.6312	5.196161	60.000004	2.566524	0
**CS complex**	21	30	NRIGNGRILA	1083.6312	5.199029	720.000061	3.432207	0
**CysK**	21	30	NRIGNGRILA	1083.6312	5.125888	0	0	0
**CysK**	21	30	NRIGNGRILA	1083.6312	5.130141	0.033	0.97921	0.063413
**CysK**	21	30	NRIGNGRILA	1083.6312	5.191394	1.667	2.268943	0.048205
**CysK**	21	30	NRIGNGRILA	1083.6312	5.144011	16.667002	2.359824	0
**CysK**	21	30	NRIGNGRILA	1083.6312	5.205925	60.000004	2.567458	0
**CysK**	21	30	NRIGNGRILA	1083.6312	5.197131	720.000061	3.427234	0
**maxD control**	21	30	NRIGNGRILA	1083.6312	5.128231	0	0	0
**maxD control**	21	30	NRIGNGRILA	1083.6312	5.408849	720.000061	4.665493	0.067568
**CS complex**	21	39	NRIGNGRILAKVESRNPSF	2128.1665	5.889297	0	0	0
**CS complex**	21	39	NRIGNGRILAKVESRNPSF	2128.1665	5.906984	0.033	1.169597	0.025641
**CS complex**	21	39	NRIGNGRILAKVESRNPSF	2128.1665	5.952604	1.667	2.20329	0.054475
**CS complex**	21	39	NRIGNGRILAKVESRNPSF	2128.1665	5.869047	16.667002	2.804719	0
**CS complex**	21	39	NRIGNGRILAKVESRNPSF	2128.1665	5.958324	60.000004	3.005291	0
**CS complex**	21	39	NRIGNGRILAKVESRNPSF	2128.1665	5.940807	720.000061	4.065095	0
**CysK**	21	39	NRIGNGRILAKVESRNPSF	2128.1665	5.895168	0	0	0
**CysK**	21	39	NRIGNGRILAKVESRNPSF	2128.1665	5.876616	0.033	1.078119	0.082922
**CysK**	21	39	NRIGNGRILAKVESRNPSF	2128.1665	5.95122	1.667	2.260607	0.043017
**CysK**	21	39	NRIGNGRILAKVESRNPSF	2128.1665	5.887828	16.667002	2.907659	0
**CysK**	21	39	NRIGNGRILAKVESRNPSF	2128.1665	5.963034	60.000004	3.098051	0
**CysK**	21	39	NRIGNGRILAKVESRNPSF	2128.1665	5.956148	720.000061	4.084836	0
**maxD control**	21	39	NRIGNGRILAKVESRNPSF	2128.1665	5.889297	0	0	0
**maxD control**	21	39	NRIGNGRILAKVESRNPSF	2128.1665	6.101482	720.000061	10.327301	0.189131
**CS complex**	30	39	AKVESRNPSF	1134.5831	4.809375	0	0	0
**CS complex**	30	39	AKVESRNPSF	1134.5831	4.849561	0.033	0.589793	0.099022
**CS complex**	30	39	AKVESRNPSF	1134.5831	4.881645	1.667	0.606321	0.087718
**CS complex**	30	39	AKVESRNPSF	1134.5831	4.828525	16.667002	0.659391	0.052732
**CS complex**	30	39	AKVESRNPSF	1134.5831	4.889725	60.000004	0.887364	0.058277
**CS complex**	30	39	AKVESRNPSF	1134.5831	4.890193	720.000061	1.010178	0.064831

**CysK**	30	39	AKVESRNPSF	1134.5831	4.808234	0	0	0
**CysK**	30	39	AKVESRNPSF	1134.5831	4.820434	0.033	0.627279	0.053727
**CysK**	30	39	AKVESRNPSF	1134.5831	4.880764	1.667	0.689607	0.038754
**CysK**	30	39	AKVESRNPSF	1134.5831	4.836774	16.667002	0.87685	0
**CysK**	30	39	AKVESRNPSF	1134.5831	4.901035	60.000004	0.963328	0
**CysK**	30	39	AKVESRNPSF	1134.5831	4.888745	720.000061	1.087162	0
**maxD control**	30	39	AKVESRNPSF	1134.5831	4.809375	0	0	0
**maxD control**	30	39	AKVESRNPSF	1134.5831	5.117542	720.000061	3.9887	0.110352
**CS complex**	31	39	KVESRNPSF	1063.546	4.769304	0	0	0
**CS complex**	31	39	KVESRNPSF	1063.546	4.812619	0.033	0.52119	0.018084
**CS complex**	31	39	KVESRNPSF	1063.546	4.842321	1.667	0.547395	0.037852
**CS complex**	31	39	KVESRNPSF	1063.546	4.797441	16.667002	0.501066	0
**CS complex**	31	39	KVESRNPSF	1063.546	4.852779	60.000004	0.809592	0
**CS complex**	31	39	KVESRNPSF	1063.546	4.852835	720.000061	0.923335	0
**CysK**	31	39	KVESRNPSF	1063.546	4.769891	0	0	0
**CysK**	31	39	KVESRNPSF	1063.546	4.783147	0.033	0.567344	0.007887
**CysK**	31	39	KVESRNPSF	1063.546	4.842317	1.667	0.656179	0.021191
**CysK**	31	39	KVESRNPSF	1063.546	4.799364	16.667002	0.812755	0
**CysK**	31	39	KVESRNPSF	1063.546	4.863231	60.000004	0.906878	0
**CysK**	31	39	KVESRNPSF	1063.546	4.852037	720.000061	1.026893	0
**maxD control**	31	39	KVESRNPSF	1063.546	4.769304	0	0	0
**maxD control**	31	39	KVESRNPSF	1063.546	5.087548	720.000061	3.070906	0.053173
**CS complex**	40	48	SVKCRIGAN	947.5025	3.873638	0	0	0
**CS complex**	40	48	SVKCRIGAN	947.5025	3.901763	0.033	0.475368	0.029648
**CS complex**	40	48	SVKCRIGAN	947.5025	3.925427	1.667	0.434518	0.038173
**CS complex**	40	48	SVKCRIGAN	947.5025	3.944007	60.000004	0.915684	0
**CS complex**	40	48	SVKCRIGAN	947.5025	3.944057	720.000061	1.461111	0
**CysK**	40	48	SVKCRIGAN	947.5025	3.862642	0	0	0
**CysK**	40	48	SVKCRIGAN	947.5025	3.890979	0.033	0.487243	0.034865
**CysK**	40	48	SVKCRIGAN	947.5025	3.92571	1.667	0.475183	0.055971
**CysK**	40	48	SVKCRIGAN	947.5025	3.921307	16.667002	0.663519	0
**CysK**	40	48	SVKCRIGAN	947.5025	3.950853	60.000004	0.930357	0
**CysK**	40	48	SVKCRIGAN	947.5025	3.943475	720.000061	1.410348	1.14E-13
**maxD control**	40	48	SVKCRIGAN	947.5025	3.873638	0	0	0
**maxD control**	40	48	SVKCRIGAN	947.5025	4.118507	720.000061	4.358877	0.101117
**CS complex**	40	49	SVKCRIGANM	1078.549596	12.935669	0	0	0
**CS complex**	40	49	SVKCRIGANM	1078.549596	12.93012	0.033	0.209059	0.025149
**CS complex**	40	49	SVKCRIGANM	1078.549596	12.931496	1.667	0.264917	0.04168
**CS complex**	40	49	SVKCRIGANM	1078.549596	12.940878	16.667002	0.351099	0
**CS complex**	40	49	SVKCRIGANM	1078.549596	12.932096	60.000004	0.496166	0
**CS complex**	40	49	SVKCRIGANM	1078.549596	12.927572	720.000061	1.099141	0

**CysK**	40	49	SVKCRIGANM	1078.549596	12.944599	0	0	0
**CysK**	40	49	SVKCRIGANM	1078.549596	12.930744	0.033	0.365281	0.021579
**CysK**	40	49	SVKCRIGANM	1078.549596	12.927894	1.667	0.414605	0.063248
**CysK**	40	49	SVKCRIGANM	1078.549596	12.939696	16.667002	0.545971	0
**CysK**	40	49	SVKCRIGANM	1078.549596	12.923409	60.000004	0.69622	0
**CysK**	40	49	SVKCRIGANM	1078.549596	12.934047	720.000061	1.211792	0
**maxD control**	40	49	SVKCRIGANM	1078.549596	12.935669	0	0	0
**maxD control**	40	49	SVKCRIGANM	1078.549596	12.940268	720.000061	4.573435	0.065831
**CS complex**	49	64	MIWDAEKRGVLKPGVE	1827.9719	6.324946	0	0	0
**CS complex**	49	64	MIWDAEKRGVLKPGVE	1827.9719	6.354744	0.033	0.933171	0.028847
**CS complex**	49	64	MIWDAEKRGVLKPGVE	1827.9719	6.390441	1.667	2.412004	0.005476
**CS complex**	49	64	MIWDAEKRGVLKPGVE	1827.9719	6.316514	16.667002	3.689338	0
**CS complex**	49	64	MIWDAEKRGVLKPGVE	1827.9719	6.389394	60.000004	4.68308	0
**CS complex**	49	64	MIWDAEKRGVLKPGVE	1827.9719	6.390778	720.000061	5.452876	0
**CysK**	49	64	MIWDAEKRGVLKPGVE	1827.9719	6.326766	0	0	0
**CysK**	49	64	MIWDAEKRGVLKPGVE	1827.9719	6.323869	0.033	0.92805	0.017248
**CysK**	49	64	MIWDAEKRGVLKPGVE	1827.9719	6.393293	1.667	2.470723	0.014313
**CysK**	49	64	MIWDAEKRGVLKPGVE	1827.9719	6.334644	16.667002	3.727867	0
**CysK**	49	64	MIWDAEKRGVLKPGVE	1827.9719	6.403585	60.000004	4.688212	0
**CysK**	49	64	MIWDAEKRGVLKPGVE	1827.9719	6.393738	720.000061	5.504509	0
**maxD control**	49	64	MIWDAEKRGVLKPGVE	1827.9719	6.324946	0	0	0
**maxD control**	49	64	MIWDAEKRGVLKPGVE	1827.9719	6.558897	720.000061	7.616238	0.131463
**CS complex**	50	64	IWDAEKRGVLKPGVE	1696.9314	5.617646	0	0	0
**CS complex**	50	64	IWDAEKRGVLKPGVE	1696.9314	5.650118	0.033	0.973894	0.010363
**CS complex**	50	64	IWDAEKRGVLKPGVE	1696.9314	5.675278	1.667	2.421231	0.029733
**CS complex**	50	64	IWDAEKRGVLKPGVE	1696.9314	5.606441	16.667002	3.869127	0
**CS complex**	50	64	IWDAEKRGVLKPGVE	1696.9314	5.677184	60.000004	4.633439	0
**CS complex**	50	64	IWDAEKRGVLKPGVE	1696.9314	5.679036	720.000061	5.441379	2.27E-13
**CysK**	50	64	IWDAEKRGVLKPGVE	1696.9314	5.617266	0	0	0
**CysK**	50	64	IWDAEKRGVLKPGVE	1696.9314	5.617086	0.033	0.992251	0.025897
**CysK**	50	64	IWDAEKRGVLKPGVE	1696.9314	5.676249	1.667	2.501203	0.060231
**CysK**	50	64	IWDAEKRGVLKPGVE	1696.9314	5.626385	16.667002	3.731442	0
**CysK**	50	64	IWDAEKRGVLKPGVE	1696.9314	5.689236	60.000004	4.700435	0
**CysK**	50	64	IWDAEKRGVLKPGVE	1696.9314	5.681273	720.000061	5.46079	0
**maxD control**	50	64	IWDAEKRGVLKPGVE	1696.9314	5.617646	0	0	0
**maxD control**	50	64	IWDAEKRGVLKPGVE	1696.9314	5.841743	720.000061	6.647201	0.130266
**CS complex**	51	64	WDAEKRGVLKPGVE	1583.8473	5.617521	0	0	0
**CS complex**	51	64	WDAEKRGVLKPGVE	1583.8473	5.651884	0.033	1.059839	0.033524
**CS complex**	51	64	WDAEKRGVLKPGVE	1583.8473	5.676098	1.667	2.50565	0.060669
**CS complex**	51	64	WDAEKRGVLKPGVE	1583.8473	5.609519	16.667002	3.743088	2.27E-13
**CS complex**	51	64	WDAEKRGVLKPGVE	1583.8473	5.677413	60.000004	4.659682	2.27E-13

**CS complex**	51	64	WDAEKRGVLKPGVE	1583.8473	5.681172	720.000061	5.399525	0
**CysK**	51	64	WDAEKRGVLKPGVE	1583.8473	5.61253	0	0	0
**CysK**	51	64	WDAEKRGVLKPGVE	1583.8473	5.617386	0.033	1.03206	0.013733
**CysK**	51	64	WDAEKRGVLKPGVE	1583.8473	5.675877	1.667	2.543653	0.021954
**CysK**	51	64	WDAEKRGVLKPGVE	1583.8473	5.623618	16.667002	3.748528	0
**CysK**	51	64	WDAEKRGVLKPGVE	1583.8473	5.687141	60.000004	4.687131	0
**CysK**	51	64	WDAEKRGVLKPGVE	1583.8473	5.678044	720.000061	5.458143	0
**maxD control**	51	64	WDAEKRGVLKPGVE	1583.8473	5.617521	0	0	0
**maxD control**	51	64	WDAEKRGVLKPGVE	1583.8473	5.841532	720.000061	6.641031	0.131998
**CS complex**	52	64	DAEKRGVLKPGVE	1397.768	5.619247	0	0	0
**CS complex**	52	64	DAEKRGVLKPGVE	1397.768	5.653696	0.033	1.019162	0.031816
**CS complex**	52	64	DAEKRGVLKPGVE	1397.768	5.679293	1.667	2.277903	0.030473
**CS complex**	52	64	DAEKRGVLKPGVE	1397.768	5.611719	16.667002	3.594903	0
**CS complex**	52	64	DAEKRGVLKPGVE	1397.768	5.681074	60.000004	4.49941	0
**CS complex**	52	64	DAEKRGVLKPGVE	1397.768	5.683495	720.000061	4.910656	0
**CysK**	52	64	DAEKRGVLKPGVE	1397.768	5.615674	0	0	0
**CysK**	52	64	DAEKRGVLKPGVE	1397.768	5.619569	0.033	0.959874	0.003913
**CysK**	52	64	DAEKRGVLKPGVE	1397.768	5.676358	1.667	2.282037	0.037929
**CysK**	52	64	DAEKRGVLKPGVE	1397.768	5.627571	16.667002	3.62063	0
**CysK**	52	64	DAEKRGVLKPGVE	1397.768	5.688124	60.000004	4.534207	0
**CysK**	52	64	DAEKRGVLKPGVE	1397.768	5.679956	720.000061	4.908259	0
**maxD control**	52	64	DAEKRGVLKPGVE	1397.768	5.619247	0	0	0
**maxD control**	52	64	DAEKRGVLKPGVE	1397.768	5.842813	720.000061	6.188017	0.115132
**CS complex**	65	77	LVEPTSGNTGIAL	1271.6775	7.336788	0	0	0
**CS complex**	65	77	LVEPTSGNTGIAL	1271.6775	7.37393	0.033	0.967434	0.042739
**CS complex**	65	77	LVEPTSGNTGIAL	1271.6775	7.412803	1.667	1.888234	0.050972
**CS complex**	65	77	LVEPTSGNTGIAL	1271.6775	7.324106	16.667002	2.727153	0
**CS complex**	65	77	LVEPTSGNTGIAL	1271.6775	7.40901	60.000004	3.0932	2.27E-13
**CS complex**	65	77	LVEPTSGNTGIAL	1271.6775	7.416716	720.000061	3.596697	0
**CysK**	65	77	LVEPTSGNTGIAL	1271.6775	7.323349	0	0	0
**CysK**	65	77	LVEPTSGNTGIAL	1271.6775	7.326609	0.033	2.695772	0.057995
**CysK**	65	77	LVEPTSGNTGIAL	1271.6775	7.405224	1.667	2.89509	0.040985
**CysK**	65	77	LVEPTSGNTGIAL	1271.6775	7.338003	16.667002	3.252101	0
**CysK**	65	77	LVEPTSGNTGIAL	1271.6775	7.412735	60.000004	3.592485	0
**CysK**	65	77	LVEPTSGNTGIAL	1271.6775	7.407242	720.000061	3.991133	0
**maxD control**	65	77	LVEPTSGNTGIAL	1271.6775	7.336788	0	0	0
**maxD control**	65	77	LVEPTSGNTGIAL	1271.6775	7.472213	720.000061	5.974509	0.13395
**CS complex**	65	78	LVEPTSGNTGIALA	1342.7146	6.868883	0	0	0
**CS complex**	65	78	LVEPTSGNTGIALA	1342.7146	6.902479	0.033	1.027031	0.036788
**CS complex**	65	78	LVEPTSGNTGIALA	1342.7146	6.939164	1.667	2.08484	0.040093
**CS complex**	65	78	LVEPTSGNTGIALA	1342.7146	6.856384	16.667002	2.94401	0

**CS complex**	65	78	LVEPTSGNTGIALA	1342.7146	6.935773	60.000004	3.277142	0
**CS complex**	65	78	LVEPTSGNTGIALA	1342.7146	6.94469	720.000061	3.722791	0
**CysK**	65	78	LVEPTSGNTGIALA	1342.7146	6.863961	0	0	0
**CysK**	65	78	LVEPTSGNTGIALA	1342.7146	6.865203	0.033	2.829996	0.082843
**CysK**	65	78	LVEPTSGNTGIALA	1342.7146	6.939236	1.667	3.085846	0.069678
**CysK**	65	78	LVEPTSGNTGIALA	1342.7146	6.879251	16.667002	3.374255	0
**CysK**	65	78	LVEPTSGNTGIALA	1342.7146	6.950416	60.000004	3.721434	0
**CysK**	65	78	LVEPTSGNTGIALA	1342.7146	6.943455	720.000061	4.051939	0
**maxD control**	65	78	LVEPTSGNTGIALA	1342.7146	6.868883	0	0	0
**maxD control**	65	78	LVEPTSGNTGIALA	1342.7146	6.994288	720.000061	6.850081	0.149136
**CS complex**	78	85	AYVAAARG	778.4141	3.783693	0	0	0
**CS complex**	78	85	AYVAAARG	778.4141	3.809299	0.033	0.399779	0.043939
**CS complex**	78	85	AYVAAARG	778.4141	3.833416	1.667	0.371272	0.042875
**CS complex**	78	85	AYVAAARG	778.4141	3.854647	60.000004	0.5994	0.021452
**CS complex**	78	85	AYVAAARG	778.4141	3.854801	720.000061	0.920071	0.036422
**CysK**	78	85	AYVAAARG	778.4141	3.772866	0	0	0
**CysK**	78	85	AYVAAARG	778.4141	3.801817	0.033	0.445221	0.018608
**CysK**	78	85	AYVAAARG	778.4141	3.829962	1.667	0.425184	0.031658
**CysK**	78	85	AYVAAARG	778.4141	3.832417	16.667002	0.602488	0.017495
**CysK**	78	85	AYVAAARG	778.4141	3.859946	60.000004	0.826251	0.021116
**CysK**	78	85	AYVAAARG	778.4141	3.850536	720.000061	0.949677	0.018269
**maxD control**	78	85	AYVAAARG	778.4141	3.783693	0	0	0
**maxD control**	78	85	AYVAAARG	778.4141	3.986922	720.000061	3.921607	0.111841
**CS complex**	78	92	AYVAAARGYKLTLTM	1628.8763	7.320876	0	0	0
**CS complex**	78	92	AYVAAARGYKLTLTM	1628.8763	7.364722	0.033	0.262003	0.009233
**CS complex**	78	92	AYVAAARGYKLTLTM	1628.8763	7.407041	1.667	0.944502	0.018148
**CS complex**	78	92	AYVAAARGYKLTLTM	1628.8763	7.3132	16.667002	1.324704	0
**CS complex**	78	92	AYVAAARGYKLTLTM	1628.8763	7.406817	60.000004	1.916334	0
**CS complex**	78	92	AYVAAARGYKLTLTM	1628.8763	7.40911	720.000061	2.44472	0
**CysK**	78	92	AYVAAARGYKLTLTM	1628.8763	7.317287	0	0	0
**CysK**	78	92	AYVAAARGYKLTLTM	1628.8763	7.316155	0.033	0.268862	0.028791
**CysK**	78	92	AYVAAARGYKLTLTM	1628.8763	7.404893	1.667	0.989552	0.015159
**CysK**	78	92	AYVAAARGYKLTLTM	1628.8763	7.325181	16.667002	1.560134	2.27E-13
**CysK**	78	92	AYVAAARGYKLTLTM	1628.8763	7.414273	60.000004	2.081857	0
**CysK**	78	92	AYVAAARGYKLTLTM	1628.8763	7.407131	720.000061	2.610247	0
**maxD control**	78	92	AYVAAARGYKLTLTM	1628.8763	7.320876	0	0	0
**maxD control**	78	92	AYVAAARGYKLTLTM	1628.8763	7.574759	720.000061	9.350631	0.214215
**CS complex**	86	92	YKLTLTM	869.4736	7.64478	0	0	0
**CS complex**	86	92	YKLTLTM	869.4736	7.68982	0.033	0.206212	0.121065
**CS complex**	86	92	YKLTLTM	869.4736	7.732866	1.667	0.261473	0.138763
**CS complex**	86	92	YKLTLTM	869.4736	7.639562	16.667002	0.358915	0.135481

**CS complex**	86	92	YKLTLTM	869.4736	7.735443	60.000004	0.657559	0.110916
**CS complex**	86	92	YKLTLTM	869.4736	7.735882	720.000061	0.858514	0.110113
**CysK**	86	92	YKLTLTM	869.4736	7.642645	0	0	0
**CysK**	86	92	YKLTLTM	869.4736	7.642012	0.033	0.261051	0.139582
**CysK**	86	92	YKLTLTM	869.4736	7.732874	1.667	0.31547	0.15378
**CysK**	86	92	YKLTLTM	869.4736	7.652475	16.667002	0.450829	0.146299
**CysK**	86	92	YKLTLTM	869.4736	7.743499	60.000004	0.76778	0.154813
**CysK**	86	92	YKLTLTM	869.4736	7.736147	720.000061	0.940677	0.151593
**maxD control**	86	92	YKLTLTM	869.4736	7.644725	0	0	0
**maxD control**	86	92	YKLTLTM	869.4736	7.877706	720.000061	3.71359	0.099078
**CS complex**	86	96	YKLTLTMPETM	1327.6573	7.995877	0	0	0
**CS complex**	86	96	YKLTLTMPETM	1327.6573	8.043142	0.033	0.686139	0.030822
**CS complex**	86	96	YKLTLTMPETM	1327.6573	8.089558	1.667	1.420956	0.014402
**CS complex**	86	96	YKLTLTMPETM	1327.6573	7.989043	16.667002	1.748222	0
**CS complex**	86	96	YKLTLTMPETM	1327.6573	8.090071	60.000004	2.225685	0
**CS complex**	86	96	YKLTLTMPETM	1327.6573	8.089013	720.000061	2.807764	0
**CysK**	86	96	YKLTLTMPETM	1327.6573	7.993813	0	0	0
**CysK**	86	96	YKLTLTMPETM	1327.6573	7.98982	0.033	0.765469	0.046144
**CysK**	86	96	YKLTLTMPETM	1327.6573	8.087451	1.667	1.495822	0.019741
**CysK**	86	96	YKLTLTMPETM	1327.6573	7.999237	16.667002	1.765485	0
**CysK**	86	96	YKLTLTMPETM	1327.6573	8.093522	60.000004	2.426583	0
**CysK**	86	96	YKLTLTMPETM	1327.6573	8.089811	720.000061	2.84598	0
**maxD control**	86	96	YKLTLTMPETM	1327.6573	7.995877	0	0	0
**maxD control**	86	96	YKLTLTMPETM	1327.6573	8.203358	720.000061	5.584001	0.078037
**CS complex**	93	110	PETMSIERRKLLKALGAN	2027.143005	6.64743	0	0	0
**CS complex**	93	110	PETMSIERRKLLKALGAN	2027.143005	6.676979	0.033	2.367212	0.103086
**CS complex**	93	110	PETMSIERRKLLKALGAN	2027.143005	6.706658	1.667	4.468675	0.030372
**CS complex**	93	110	PETMSIERRKLLKALGAN	2027.143005	6.626177	16.667002	5.778984	2.27E-13
**CS complex**	93	110	PETMSIERRKLLKALGAN	2027.143005	6.707393	60.000004	6.079706	2.27E-13
**CS complex**	93	110	PETMSIERRKLLKALGAN	2027.143005	6.703109	720.000061	6.433217	0
**CysK**	93	110	PETMSIERRKLLKALGAN	2027.143005	6.653009	0	0	0
**CysK**	93	110	PETMSIERRKLLKALGAN	2027.143005	6.638365	0.033	2.390235	0.087427
**CysK**	93	110	PETMSIERRKLLKALGAN	2027.143005	6.707331	1.667	5.171378	0.04482
**CysK**	93	110	PETMSIERRKLLKALGAN	2027.143005	6.637888	16.667002	5.785101	0
**CysK**	93	110	PETMSIERRKLLKALGAN	2027.143005	6.719382	60.000004	6.157969	0
**CysK**	93	110	PETMSIERRKLLKALGAN	2027.143005	6.707265	720.000061	6.594017	0
**maxD control**	93	110	PETMSIERRKLLKALGAN	2027.143005	6.647501	0	0	0
**maxD control**	93	110	PETMSIERRKLLKALGAN	2027.143005	6.859088	720.000061	10.619371	0.160218
**CS complex**	97	110	SIERRKLLKALGAN	1568.9531	5.332097	0	0	0
**CS complex**	97	110	SIERRKLLKALGAN	1568.9531	5.357414	0.033	0.697255	0.019344
**CS complex**	97	110	SIERRKLLKALGAN	1568.9531	5.37729	1.667	1.675729	0.021086

**CS complex**	97	110	SIERRKLLKALGAN	1568.9531	5.282727	16.667002	3.076889	0.0341
**CS complex**	97	110	SIERRKLLKALGAN	1568.9531	5.372467	60.000004	3.09436	0
**CS complex**	97	110	SIERRKLLKALGAN	1568.9531	5.36961	720.000061	3.598857	0
**CysK**	97	110	SIERRKLLKALGAN	1568.9531	5.334384	0	0	0
**CysK**	97	110	SIERRKLLKALGAN	1568.9531	5.325775	0.033	0.773559	0.05574
**CysK**	97	110	SIERRKLLKALGAN	1568.9531	5.373889	1.667	2.388558	0.045268
**CysK**	97	110	SIERRKLLKALGAN	1568.9531	5.317193	16.667002	3.267653	2.27E-13
**CysK**	97	110	SIERRKLLKALGAN	1568.9531	5.379927	60.000004	3.345191	2.27E-13
**CysK**	97	110	SIERRKLLKALGAN	1568.9531	5.375087	720.000061	3.586054	2.27E-13
**maxD control**	97	110	SIERRKLLKALGAN	1568.9531	5.332097	0	0	0
**maxD control**	97	110	SIERRKLLKALGAN	1568.9531	5.572282	720.000061	7.549397	0.154236
**CS complex**	97	111	SIERRKLLKALGANL	1682.0371	6.365433	0	0	0
**CS complex**	97	111	SIERRKLLKALGANL	1682.0371	6.389384	0.033	0.854311	0.013998
**CS complex**	97	111	SIERRKLLKALGANL	1682.0371	6.427016	1.667	2.461279	0.049048
**CS complex**	97	111	SIERRKLLKALGANL	1682.0371	6.345266	16.667002	3.366079	0
**CS complex**	97	111	SIERRKLLKALGANL	1682.0371	6.420273	60.000004	3.655975	0
**CS complex**	97	111	SIERRKLLKALGANL	1682.0371	6.415831	720.000061	4.066492	0
**CysK**	97	111	SIERRKLLKALGANL	1682.0371	6.371701	0	0	0
**CysK**	97	111	SIERRKLLKALGANL	1682.0371	6.356363	0.033	0.948106	0.102985
**CysK**	97	111	SIERRKLLKALGANL	1682.0371	6.426132	1.667	2.998343	0.049064
**CysK**	97	111	SIERRKLLKALGANL	1682.0371	6.358994	16.667002	3.734992	2.27E-13
**CysK**	97	111	SIERRKLLKALGANL	1682.0371	6.430713	60.000004	3.918702	2.27E-13
**CysK**	97	111	SIERRKLLKALGANL	1682.0371	6.426284	720.000061	4.20882	2.27E-13
**maxD control**	97	111	SIERRKLLKALGANL	1682.0371	6.365433	0	0	0
**maxD control**	97	111	SIERRKLLKALGANL	1682.0371	6.611701	720.000061	8.379267	0.123575
**CS complex**	99	111	ERRKLLKALGANL	1481.927457	5.866338	0	0	0
**CS complex**	99	111	ERRKLLKALGANL	1481.927457	5.889135	0.033	0.540898	0.066484
**CS complex**	99	111	ERRKLLKALGANL	1481.927457	5.924252	1.667	1.46814	0.016142
**CS complex**	99	111	ERRKLLKALGANL	1481.927457	5.854864	16.667002	2.392577	0
**CS complex**	99	111	ERRKLLKALGANL	1481.927457	5.926644	60.000004	2.576756	0
**CS complex**	99	111	ERRKLLKALGANL	1481.927457	5.920356	720.000061	3.042631	0
**CysK**	99	111	ERRKLLKALGANL	1481.927457	5.881496	0	0	0
**CysK**	99	111	ERRKLLKALGANL	1481.927457	5.855325	0.033	0.672137	0.055545
**CysK**	99	111	ERRKLLKALGANL	1481.927457	5.9259	1.667	1.99752	0.038384
**CysK**	99	111	ERRKLLKALGANL	1481.927457	5.869493	16.667002	2.572333	0
**CysK**	99	111	ERRKLLKALGANL	1481.927457	5.941369	60.000004	2.737264	0
**CysK**	99	111	ERRKLLKALGANL	1481.927457	5.931859	720.000061	3.132206	0
**maxD control**	99	111	ERRKLLKALGANL	1481.927457	5.866338	0	0	0
**maxD control**	99	111	ERRKLLKALGANL	1481.927457	6.117393	720.000061	7.20198	0.095506
**CS complex**	100	110	RRKLLKALGAN	1239.7944	4.211081	0	0	0
**CS complex**	100	110	RRKLLKALGAN	1239.7944	4.224638	0.033	0.296922	0.124

**CS complex**	100	110	RRKLLKALGAN	1239.7944	4.259208	1.667	0.612986	0.140554
**CS complex**	100	110	RRKLLKALGAN	1239.7944	4.267313	60.000004	1.363062	0.103978
**CS complex**	100	110	RRKLLKALGAN	1239.7944	4.267731	720.000061	1.718758	0.104619
**CysK**	100	110	RRKLLKALGAN	1239.7944	4.22832	0	0	0
**CysK**	100	110	RRKLLKALGAN	1239.7944	4.211483	0.033	0.311705	0.093769
**CysK**	100	110	RRKLLKALGAN	1239.7944	4.260682	1.667	0.807372	0.107437
**CysK**	100	110	RRKLLKALGAN	1239.7944	4.242698	16.667002	1.291724	0.071336
**CysK**	100	110	RRKLLKALGAN	1239.7944	4.275091	60.000004	1.425113	0.063329
**CysK**	100	110	RRKLLKALGAN	1239.7944	4.277158	720.000061	1.762706	0.083883
**maxD control**	100	110	RRKLLKALGAN	1239.7944	4.210995	0	0	0
**maxD control**	100	110	RRKLLKALGAN	1239.7944	4.524003	720.000061	5.584433	0.182259
**CS complex**	102	117	KLLKALGANLVLTEGA	1610.983969	5.348237	0	0	0
**CS complex**	102	117	KLLKALGANLVLTEGA	1610.983969	5.374828	0.033	1.153041	0.035479
**CS complex**	102	117	KLLKALGANLVLTEGA	1610.983969	5.387486	1.667	2.511999	0.050597
**CS complex**	102	117	KLLKALGANLVLTEGA	1610.983969	5.391675	60.000004	3.167163	0
**CS complex**	102	117	KLLKALGANLVLTEGA	1610.983969	5.395891	720.000061	3.538283	0
**CysK**	102	117	KLLKALGANLVLTEGA	1610.983969	5.348661	0	0	0
**CysK**	102	117	KLLKALGANLVLTEGA	1610.983969	5.340912	0.033	1.39209	0.112305
**CysK**	102	117	KLLKALGANLVLTEGA	1610.983969	5.391135	1.667	2.669224	0.02115
**CysK**	102	117	KLLKALGANLVLTEGA	1610.983969	5.336201	16.667002	3.246929	0
**CysK**	102	117	KLLKALGANLVLTEGA	1610.983969	5.403131	60.000004	3.135618	0
**CysK**	102	117	KLLKALGANLVLTEGA	1610.983969	5.393451	720.000061	3.687199	0
**maxD control**	102	117	KLLKALGANLVLTEGA	1610.983969	5.348237	0	0	0
**maxD control**	102	117	KLLKALGANLVLTEGA	1610.983969	5.577875	720.000061	7.504585	0.197525
**CS complex**	111	125	LVLTEGAKGMKGAIQ	1515.8505	5.839618	0	0	0
**CS complex**	111	125	LVLTEGAKGMKGAIQ	1515.8505	5.865104	0.033	3.712033	0.047684
**CS complex**	111	125	LVLTEGAKGMKGAIQ	1515.8505	5.895078	1.667	5.520252	0.076675
**CS complex**	111	125	LVLTEGAKGMKGAIQ	1515.8505	5.817886	16.667002	6.935213	0
**CS complex**	111	125	LVLTEGAKGMKGAIQ	1515.8505	5.89328	60.000004	7.934558	0
**CS complex**	111	125	LVLTEGAKGMKGAIQ	1515.8505	5.891148	720.000061	8.950795	0
**CysK**	111	125	LVLTEGAKGMKGAIQ	1515.8505	5.833839	0	0	0
**CysK**	111	125	LVLTEGAKGMKGAIQ	1515.8505	5.828665	0.033	3.906087	0.123791
**CysK**	111	125	LVLTEGAKGMKGAIQ	1515.8505	5.891192	1.667	6.417511	0.132262
**CysK**	111	125	LVLTEGAKGMKGAIQ	1515.8505	5.830558	16.667002	8.023548	2.27E-13
**CysK**	111	125	LVLTEGAKGMKGAIQ	1515.8505	5.900699	60.000004	8.635926	0
**CysK**	111	125	LVLTEGAKGMKGAIQ	1515.8505	5.891234	720.000061	9.150401	0
**maxD control**	111	125	LVLTEGAKGMKGAIQ	1515.8505	5.839618	0	0	0
**maxD control**	111	125	LVLTEGAKGMKGAIQ	1515.8505	6.044289	720.000061	8.180194	0.192254
**CS complex**	111	129	LVLTEGAKGMKGAIQKAEE	1973.0676	6.147523	0	0	0
**CS complex**	111	129	LVLTEGAKGMKGAIQKAEE	1973.0676	6.163605	0.033	3.477219	0.177098
**CS complex**	111	129	LVLTEGAKGMKGAIQKAEE	1973.0676	6.187812	1.667	5.306687	0.163839

**CS complex**	111	129	LVLTEGAKGMKGAIQKAEE	1973.0676	6.086068	16.667002	6.962193	0.145807
**CS complex**	111	129	LVLTEGAKGMKGAIQKAEE	1973.0676	6.178137	60.000004	7.812679	0.146395
**CS complex**	111	129	LVLTEGAKGMKGAIQKAEE	1973.0676	6.168306	720.000061	9.422935	0.145024
**CysK**	111	129	LVLTEGAKGMKGAIQKAEE	1973.0676	6.142937	0	0	0
**CysK**	111	129	LVLTEGAKGMKGAIQKAEE	1973.0676	6.123413	0.033	3.620249	0.199261
**CysK**	111	129	LVLTEGAKGMKGAIQKAEE	1973.0676	6.181689	1.667	6.15531	0.249507
**CysK**	111	129	LVLTEGAKGMKGAIQKAEE	1973.0676	6.108799	16.667002	7.943144	0.150244
**CysK**	111	129	LVLTEGAKGMKGAIQKAEE	1973.0676	6.178485	60.000004	8.668791	0.150053
**CysK**	111	129	LVLTEGAKGMKGAIQKAEE	1973.0676	6.162222	720.000061	9.954066	0.150244
**maxD control**	111	129	LVLTEGAKGMKGAIQKAEE	1973.0676	6.147523	0	0	0
**maxD control**	111	129	LVLTEGAKGMKGAIQKAEE	1973.0676	6.303717	720.000061	10.536867	0.324029
**CS complex**	112	129	VLTEGAKGMKGAIQKAEE	1859.9835	5.338923	0	0	0
**CS complex**	112	129	VLTEGAKGMKGAIQKAEE	1859.9835	5.357184	0.033	3.280132	0.159875
**CS complex**	112	129	VLTEGAKGMKGAIQKAEE	1859.9835	5.37549	1.667	4.884333	0.152952
**CS complex**	112	129	VLTEGAKGMKGAIQKAEE	1859.9835	5.278484	16.667002	6.16061	0.134927
**CS complex**	112	129	VLTEGAKGMKGAIQKAEE	1859.9835	5.370162	60.000004	6.782904	0.129713
**CS complex**	112	129	VLTEGAKGMKGAIQKAEE	1859.9835	5.361105	720.000061	8.389052	0.147767
**CysK**	112	129	VLTEGAKGMKGAIQKAEE	1859.9835	5.333129	0	0	0
**CysK**	112	129	VLTEGAKGMKGAIQKAEE	1859.9835	5.324707	0.033	3.473353	0.195661
**CysK**	112	129	VLTEGAKGMKGAIQKAEE	1859.9835	5.371688	1.667	5.549699	0.213533
**CysK**	112	129	VLTEGAKGMKGAIQKAEE	1859.9835	5.306393	16.667002	7.072496	0.15571
**CysK**	112	129	VLTEGAKGMKGAIQKAEE	1859.9835	5.370654	60.000004	7.756494	0.159539
**CysK**	112	129	VLTEGAKGMKGAIQKAEE	1859.9835	5.355173	720.000061	8.928101	0.152216
**maxD control**	112	129	VLTEGAKGMKGAIQKAEE	1859.9835	5.338923	0	0	0
**maxD control**	112	129	VLTEGAKGMKGAIQKAEE	1859.9835	5.517506	720.000061	9.572325	0.327663
**CS complex**	113	129	LTEGAKGMKGAIQKAEE	1760.9152	6.147094	0	0	0
**CS complex**	113	129	LTEGAKGMKGAIQKAEE	1760.9152	6.165846	0.033	3.257861	0.226251
**CS complex**	113	129	LTEGAKGMKGAIQKAEE	1760.9152	6.189451	1.667	4.979792	0.192899
**CS complex**	113	129	LTEGAKGMKGAIQKAEE	1760.9152	6.086328	16.667002	6.541336	0.186283
**CS complex**	113	129	LTEGAKGMKGAIQKAEE	1760.9152	6.17889	60.000004	7.277478	0.199654
**CS complex**	113	129	LTEGAKGMKGAIQKAEE	1760.9152	6.169586	720.000061	8.759959	0.191398
**CysK**	113	129	LTEGAKGMKGAIQKAEE	1760.9152	6.142509	0	0	0
**CysK**	113	129	LTEGAKGMKGAIQKAEE	1760.9152	6.124493	0.033	3.453812	0.222727
**CysK**	113	129	LTEGAKGMKGAIQKAEE	1760.9152	6.182212	1.667	5.766343	0.221377
**CysK**	113	129	LTEGAKGMKGAIQKAEE	1760.9152	6.110374	16.667002	7.459956	0.18096
**CysK**	113	129	LTEGAKGMKGAIQKAEE	1760.9152	6.179813	60.000004	8.105683	0.182493
**CysK**	113	129	LTEGAKGMKGAIQKAEE	1760.9152	6.161276	720.000061	9.343233	0.190531
**maxD control**	113	129	LTEGAKGMKGAIQKAEE	1760.9152	6.147094	0	0	0
**maxD control**	113	129	LTEGAKGMKGAIQKAEE	1760.9152	6.305015	720.000061	10.035421	0.357407
**CS complex**	114	129	TEGAKGMKGAIQKAEE	1647.831	6.144722	0	0	0
**CS complex**	114	129	TEGAKGMKGAIQKAEE	1647.831	6.156093	0.033	3.219951	0.24257

**CS complex**	114	129	TEGAKGMKGAIQKAEE	1647.831	6.185628	1.667	4.911884	0.18886
**CS complex**	114	129	TEGAKGMKGAIQKAEE	1647.831	6.085247	16.667002	6.437638	0.255543
**CS complex**	114	129	TEGAKGMKGAIQKAEE	1647.831	6.173954	60.000004	7.148111	0.247805
**CS complex**	114	129	TEGAKGMKGAIQKAEE	1647.831	6.162316	720.000061	8.571451	0.203728
**CysK**	114	129	TEGAKGMKGAIQKAEE	1647.831	6.142352	0	0	0
**CysK**	114	129	TEGAKGMKGAIQKAEE	1647.831	6.122426	0.033	3.444514	0.139629
**CysK**	114	129	TEGAKGMKGAIQKAEE	1647.831	6.180807	1.667	5.709958	0.190216
**CysK**	114	129	TEGAKGMKGAIQKAEE	1647.831	6.108626	16.667002	7.331739	0.172936
**CysK**	114	129	TEGAKGMKGAIQKAEE	1647.831	6.177139	60.000004	7.925022	0.185384
**CysK**	114	129	TEGAKGMKGAIQKAEE	1647.831	6.160555	720.000061	9.052916	0.255928
**maxD control**	114	129	TEGAKGMKGAIQKAEE	1647.831	6.144722	0	0	0
**maxD control**	114	129	TEGAKGMKGAIQKAEE	1647.831	6.30696	720.000061	9.659379	0.265685
**CS complex**	130	138	IVASNPEKY	1020.5299	4.900314	0	0	0
**CS complex**	130	138	IVASNPEKY	1020.5299	4.938551	0.033	0.70966	0.026377
**CS complex**	130	138	IVASNPEKY	1020.5299	4.962554	1.667	2.011859	0.048149
**CS complex**	130	138	IVASNPEKY	1020.5299	4.912745	16.667002	2.36681	1.61E-13
**CS complex**	130	138	IVASNPEKY	1020.5299	4.971045	60.000004	2.490284	1.14E-13
**CS complex**	130	138	IVASNPEKY	1020.5299	4.96854	720.000061	2.853392	1.14E-13
**CysK**	130	138	IVASNPEKY	1020.5299	4.902336	0	0	0
**CysK**	130	138	IVASNPEKY	1020.5299	4.910479	0.033	0.706056	0.035596
**CysK**	130	138	IVASNPEKY	1020.5299	4.962643	1.667	2.142293	0.064085
**CysK**	130	138	IVASNPEKY	1020.5299	4.921865	16.667002	2.371621	1.14E-13
**CysK**	130	138	IVASNPEKY	1020.5299	4.978951	60.000004	2.550937	1.14E-13
**CysK**	130	138	IVASNPEKY	1020.5299	4.966756	720.000061	2.940279	1.14E-13
**maxD control**	130	138	IVASNPEKY	1020.5299	4.900314	0	0	0
**maxD control**	130	138	IVASNPEKY	1020.5299	5.100392	720.000061	3.597086	0.091219
**CS complex**	130	140	IVASNPEKYLL	1246.6979	7.721302	0	0	0
**CS complex**	130	140	IVASNPEKYLL	1246.6979	7.766295	0.033	0.470256	0.040605
**CS complex**	130	140	IVASNPEKYLL	1246.6979	7.809861	1.667	1.540779	0.057475
**CS complex**	130	140	IVASNPEKYLL	1246.6979	7.711622	16.667002	2.11112	0
**CS complex**	130	140	IVASNPEKYLL	1246.6979	7.81032	60.000004	2.541225	0
**CS complex**	130	140	IVASNPEKYLL	1246.6979	7.812012	720.000061	3.139724	0
**CysK**	130	140	IVASNPEKYLL	1246.6979	7.716197	0	0	0
**CysK**	130	140	IVASNPEKYLL	1246.6979	7.71597	0.033	0.657917	0.031262
**CysK**	130	140	IVASNPEKYLL	1246.6979	7.80746	1.667	1.864397	0.059255
**CysK**	130	140	IVASNPEKYLL	1246.6979	7.725213	16.667002	2.476401	0
**CysK**	130	140	IVASNPEKYLL	1246.6979	7.813963	60.000004	2.81841	0
**CysK**	130	140	IVASNPEKYLL	1246.6979	7.808412	720.000061	3.368208	0
**maxD control**	130	140	IVASNPEKYLL	1246.6979	7.721134	0	0	0
**maxD control**	130	140	IVASNPEKYLL	1246.6979	7.949271	720.000061	4.737295	0.100043
**CS complex**	130	141	IVASNPEKYLLL	1359.7819	8.574184	0	0	0

**CS complex**	130	141	IVASNPEKYLLL	1359.7819	8.618691	0.033	0.565629	0.027674
**CS complex**	130	141	IVASNPEKYLLL	1359.7819	8.666426	1.667	1.548064	0.044519
**CS complex**	130	141	IVASNPEKYLLL	1359.7819	8.57186	16.667002	2.149747	0
**CS complex**	130	141	IVASNPEKYLLL	1359.7819	8.662721	60.000004	2.577778	0
**CS complex**	130	141	IVASNPEKYLLL	1359.7819	8.667206	720.000061	3.386395	0
**CysK**	130	141	IVASNPEKYLLL	1359.7819	8.568001	0	0	0
**CysK**	130	141	IVASNPEKYLLL	1359.7819	8.5672	0.033	0.530608	0.040999
**CysK**	130	141	IVASNPEKYLLL	1359.7819	8.66368	1.667	1.684053	0.050989
**CysK**	130	141	IVASNPEKYLLL	1359.7819	8.577326	16.667002	2.221058	0
**CysK**	130	141	IVASNPEKYLLL	1359.7819	8.671788	60.000004	2.750016	0
**CysK**	130	141	IVASNPEKYLLL	1359.7819	8.664648	720.000061	3.779313	0
**maxD control**	130	141	IVASNPEKYLLL	1359.7819	8.574184	0	0	0
**maxD control**	130	141	IVASNPEKYLLL	1359.7819	8.807031	720.000061	5.317437	0.09465
**CS complex**	142	154	QQFSNPANPEIHE	1510.6858	5.592453	0	0	0
**CS complex**	142	154	QQFSNPANPEIHE	1510.6858	5.62968	0.033	0.880604	0.065676
**CS complex**	142	154	QQFSNPANPEIHE	1510.6858	5.653893	1.667	1.502704	0.049296
**CS complex**	142	154	QQFSNPANPEIHE	1510.6858	5.590767	16.667002	1.853069	0
**CS complex**	142	154	QQFSNPANPEIHE	1510.6858	5.65928	60.000004	2.009232	0
**CS complex**	142	154	QQFSNPANPEIHE	1510.6858	5.66124	720.000061	2.780156	0
**CysK**	142	154	QQFSNPANPEIHE	1510.6858	5.585183	0	0	0
**CysK**	142	154	QQFSNPANPEIHE	1510.6858	5.591839	0.033	0.934501	0.010154
**CysK**	142	154	QQFSNPANPEIHE	1510.6858	5.646762	1.667	1.658741	0.079752
**CysK**	142	154	QQFSNPANPEIHE	1510.6858	5.599135	16.667002	2.182811	0
**CysK**	142	154	QQFSNPANPEIHE	1510.6858	5.662573	60.000004	2.238446	0
**CysK**	142	154	QQFSNPANPEIHE	1510.6858	5.653265	720.000061	3.215255	0
**maxD control**	142	154	QQFSNPANPEIHE	1510.6858	5.592453	0	0	0
**maxD control**	142	154	QQFSNPANPEIHE	1510.6858	5.748199	720.000061	5.124794	0.160463
**CS complex**	144	154	FSNPANPEIHE	1254.5686	5.496301	0	0	0
**CS complex**	144	154	FSNPANPEIHE	1254.5686	5.529365	0.033	0.720879	0.024673
**CS complex**	144	154	FSNPANPEIHE	1254.5686	5.562713	1.667	0.682731	0.061239
**CS complex**	144	154	FSNPANPEIHE	1254.5686	5.545352	16.667002	0.534937	0
**CS complex**	144	154	FSNPANPEIHE	1254.5686	5.567097	60.000004	0.699456	0
**CS complex**	144	154	FSNPANPEIHE	1254.5686	5.569135	720.000061	1.398807	0
**CysK**	144	154	FSNPANPEIHE	1254.5686	5.491408	0	0	0
**CysK**	144	154	FSNPANPEIHE	1254.5686	5.497879	0.033	0.678239	0.026928
**CysK**	144	154	FSNPANPEIHE	1254.5686	5.563335	1.667	0.70205	0.051508
**CysK**	144	154	FSNPANPEIHE	1254.5686	5.509929	16.667002	0.696718	0
**CysK**	144	154	FSNPANPEIHE	1254.5686	5.575793	60.000004	0.890347	0
**CysK**	144	154	FSNPANPEIHE	1254.5686	5.568534	720.000061	1.793232	0
**maxD control**	144	154	FSNPANPEIHE	1254.5686	5.496301	0	0	0
**maxD control**	144	154	FSNPANPEIHE	1254.5686	5.664002	720.000061	3.786867	0.168513

**CS complex**	147	161	PANPEIHEKTTGPEI	1632.8164	5.930828	0	0	0
**CS complex**	147	161	PANPEIHEKTTGPEI	1632.8164	5.965939	0.033	0.783348	0.013667
**CS complex**	147	161	PANPEIHEKTTGPEI	1632.8164	5.998353	1.667	0.787627	0.080728
**CS complex**	147	161	PANPEIHEKTTGPEI	1632.8164	5.918642	16.667002	0.790134	2.27E-13
**CS complex**	147	161	PANPEIHEKTTGPEI	1632.8164	5.999107	60.000004	0.737362	2.27E-13
**CS complex**	147	161	PANPEIHEKTTGPEI	1632.8164	6.003958	720.000061	1.403452	2.27E-13
**CysK**	147	161	PANPEIHEKTTGPEI	1632.8164	5.931142	0	0	0
**CysK**	147	161	PANPEIHEKTTGPEI	1632.8164	5.930998	0.033	0.742701	0.052274
**CysK**	147	161	PANPEIHEKTTGPEI	1632.8164	5.999459	1.667	0.828579	0.112675
**CysK**	147	161	PANPEIHEKTTGPEI	1632.8164	5.943592	16.667002	0.893315	0
**CysK**	147	161	PANPEIHEKTTGPEI	1632.8164	6.014709	60.000004	0.911853	0
**CysK**	147	161	PANPEIHEKTTGPEI	1632.8164	6.00454	720.000061	1.665414	0
**maxD control**	147	161	PANPEIHEKTTGPEI	1632.8164	5.930828	0	0	0
**maxD control**	147	161	PANPEIHEKTTGPEI	1632.8164	6.166997	720.000061	6.227288	0.186597
**CS complex**	155	163	KTTGPEIWE	1060.5244	6.976603	0	0	0
**CS complex**	155	163	KTTGPEIWE	1060.5244	7.021844	0.033	0.309754	0.030158
**CS complex**	155	163	KTTGPEIWE	1060.5244	7.066173	1.667	0.316723	0.038299
**CS complex**	155	163	KTTGPEIWE	1060.5244	6.973067	16.667002	0.290303	0
**CS complex**	155	163	KTTGPEIWE	1060.5244	7.073381	60.000004	0.234142	0
**CS complex**	155	163	KTTGPEIWE	1060.5244	7.067266	720.000061	0.368116	0
**CysK**	155	163	KTTGPEIWE	1060.5244	6.974663	0	0	0
**CysK**	155	163	KTTGPEIWE	1060.5244	6.978564	0.033	0.321373	0.012272
**CysK**	155	163	KTTGPEIWE	1060.5244	7.0644	1.667	0.321785	0.053465
**CysK**	155	163	KTTGPEIWE	1060.5244	6.986749	16.667002	0.31522	0
**CysK**	155	163	KTTGPEIWE	1060.5244	7.078753	60.000004	0.257685	0
**CysK**	155	163	KTTGPEIWE	1060.5244	7.068541	720.000061	0.332134	0
**maxD control**	155	163	KTTGPEIWE	1060.5244	6.976603	0	0	0
**maxD control**	155	163	KTTGPEIWE	1060.5244	7.219446	720.000061	3.759368	0.157482
**CS complex**	162	172	WEDTDGQVDVF	1310.547	8.747394	0	0	0
**CS complex**	162	172	WEDTDGQVDVF	1310.547	8.804814	0.033	0.416443	0.043335
**CS complex**	162	172	WEDTDGQVDVF	1310.547	8.843768	1.667	1.161056	0.062827
**CS complex**	162	172	WEDTDGQVDVF	1310.547	8.743958	16.667002	1.438003	0.05031
**CS complex**	162	172	WEDTDGQVDVF	1310.547	8.847433	60.000004	1.802475	0.163156
**CS complex**	162	172	WEDTDGQVDVF	1310.547	8.84719	720.000061	2.560119	0.108067
**CysK**	162	172	WEDTDGQVDVF	1310.547	8.746188	0	0	0
**CysK**	162	172	WEDTDGQVDVF	1310.547	8.75122	0.033	0.408497	0.034135
**CysK**	162	172	WEDTDGQVDVF	1310.547	8.842526	1.667	1.168159	0.049123
**CysK**	162	172	WEDTDGQVDVF	1310.547	8.756292	16.667002	1.560974	0.062934
**CysK**	162	172	WEDTDGQVDVF	1310.547	8.855559	60.000004	1.763875	0.125227
**CysK**	162	172	WEDTDGQVDVF	1310.547	8.845284	720.000061	2.423639	0.145575
**maxD control**	162	172	WEDTDGQVDVF	1310.547	8.747394	0	0	0

**maxD control**	162	172	WEDTDGQVDVF	1310.547	8.921339	720.000061	4.043484	0.238062
**CS complex**	163	172	EDTDGQVDVF	1124.4677	7.691687	0	0	0
**CS complex**	163	172	EDTDGQVDVF	1124.4677	7.735795	0.033	0.447412	0.013091
**CS complex**	163	172	EDTDGQVDVF	1124.4677	7.772322	1.667	1.226271	0.02536
**CS complex**	163	172	EDTDGQVDVF	1124.4677	7.687296	16.667002	1.50324	0
**CS complex**	163	172	EDTDGQVDVF	1124.4677	7.776271	60.000004	1.941778	0
**CS complex**	163	172	EDTDGQVDVF	1124.4677	7.775086	720.000061	2.690185	0
**CysK**	163	172	EDTDGQVDVF	1124.4677	7.687206	0	0	0
**CysK**	163	172	EDTDGQVDVF	1124.4677	7.691699	0.033	0.422292	0.019388
**CysK**	163	172	EDTDGQVDVF	1124.4677	7.770535	1.667	1.226374	0.024532
**CysK**	163	172	EDTDGQVDVF	1124.4677	7.698156	16.667002	1.549262	0
**CysK**	163	172	EDTDGQVDVF	1124.4677	7.783896	60.000004	1.934308	0
**CysK**	163	172	EDTDGQVDVF	1124.4677	7.773328	720.000061	2.530596	0
**maxD control**	163	172	EDTDGQVDVF	1124.4677	7.691687	0	0	0
**maxD control**	163	172	EDTDGQVDVF	1124.4677	7.855979	720.000061	3.987876	0.08783
**CS complex**	172	198	FIAGVGTGGTLTGVSRYIKGTKGKTDL	2697.4868	6.808139	0	0	0
**CS complex**	172	198	FIAGVGTGGTLTGVSRYIKGTKGKTDL	2697.4868	6.841708	0.033	2.477914	0.06155
**CS complex**	172	198	FIAGVGTGGTLTGVSRYIKGTKGKTDL	2697.4868	6.881789	1.667	3.762762	0.049278
**CS complex**	172	198	FIAGVGTGGTLTGVSRYIKGTKGKTDL	2697.4868	6.791623	16.667002	4.498743	0.016447
**CS complex**	172	198	FIAGVGTGGTLTGVSRYIKGTKGKTDL	2697.4868	6.884814	60.000004	4.936598	0.021673
**CS complex**	172	198	FIAGVGTGGTLTGVSRYIKGTKGKTDL	2697.4868	6.881583	720.000061	5.071446	0.069348
**CysK**	172	198	FIAGVGTGGTLTGVSRYIKGTKGKTDL	2697.4868	6.804217	0	0	0
**CysK**	172	198	FIAGVGTGGTLTGVSRYIKGTKGKTDL	2697.4868	6.795965	0.033	2.296716	0.117286
**CysK**	172	198	FIAGVGTGGTLTGVSRYIKGTKGKTDL	2697.4868	6.881841	1.667	3.805649	0.120727
**CysK**	172	198	FIAGVGTGGTLTGVSRYIKGTKGKTDL	2697.4868	6.803178	16.667002	4.752272	0.109316
**CysK**	172	198	FIAGVGTGGTLTGVSRYIKGTKGKTDL	2697.4868	6.886129	60.000004	5.062606	0.053025
**CysK**	172	198	FIAGVGTGGTLTGVSRYIKGTKGKTDL	2697.4868	6.877083	720.000061	5.192918	0.058128
**maxD control**	172	198	FIAGVGTGGTLTGVSRYIKGTKGKTDL	2697.4868	6.808139	0	0	0
**maxD control**	172	198	FIAGVGTGGTLTGVSRYIKGTKGKTDL	2697.4868	6.989143	720.000061	15.204287	0.542299
**CS complex**	173	186	IAGVGTGGTLTGVS	1189.6359	6.377246	0	0	0
**CS complex**	173	186	IAGVGTGGTLTGVS	1189.6359	6.403476	0.033	0.831882	0.079633
**CS complex**	173	186	IAGVGTGGTLTGVS	1189.6359	6.435372	1.667	0.823457	0.107905
**CS complex**	173	186	IAGVGTGGTLTGVS	1189.6359	6.36473	16.667002	0.709959	0.050606
**CS complex**	173	186	IAGVGTGGTLTGVS	1189.6359	6.435781	60.000004	0.707358	0.051106
**CS complex**	173	186	IAGVGTGGTLTGVS	1189.6359	6.441864	720.000061	0.872914	0.040181
**CysK**	173	186	IAGVGTGGTLTGVS	1189.6359	6.364838	0	0	0
**CysK**	173	186	IAGVGTGGTLTGVS	1189.6359	6.368618	0.033	0.696127	0.082516
**CysK**	173	186	IAGVGTGGTLTGVS	1189.6359	6.436005	1.667	0.74525	0.113245
**CysK**	173	186	IAGVGTGGTLTGVS	1189.6359	6.384357	16.667002	0.781888	0.050682
**CysK**	173	186	IAGVGTGGTLTGVS	1189.6359	6.447774	60.000004	0.704619	0.042637
**CysK**	173	186	IAGVGTGGTLTGVS	1189.6359	6.440892	720.000061	0.871596	0.051324

**maxD control**	173	186	IAGVGTGGTLTGVS	1189.6359	6.377246	0	0	0
**maxD control**	173	186	IAGVGTGGTLTGVS	1189.6359	6.491658	720.000061	7.178868	0.388742
**CS complex**	173	198	IAGVGTGGTLTGVSRYIKGTKGKTDL	2550.4185	6.43241	0	0	0
**CS complex**	173	198	IAGVGTGGTLTGVSRYIKGTKGKTDL	2550.4185	6.467838	0.033	2.387938	0.185838
**CS complex**	173	198	IAGVGTGGTLTGVSRYIKGTKGKTDL	2550.4185	6.499755	1.667	3.671385	0.17708
**CS complex**	173	198	IAGVGTGGTLTGVSRYIKGTKGKTDL	2550.4185	6.418024	16.667002	4.402457	0.171337
**CS complex**	173	198	IAGVGTGGTLTGVSRYIKGTKGKTDL	2550.4185	6.50318	60.000004	4.780008	0.175692
**CS complex**	173	198	IAGVGTGGTLTGVSRYIKGTKGKTDL	2550.4185	6.505498	720.000061	4.904401	0.166998
**CysK**	173	198	IAGVGTGGTLTGVSRYIKGTKGKTDL	2550.4185	6.428047	0	0	0
**CysK**	173	198	IAGVGTGGTLTGVSRYIKGTKGKTDL	2550.4185	6.422157	0.033	2.163432	0.184348
**CysK**	173	198	IAGVGTGGTLTGVSRYIKGTKGKTDL	2550.4185	6.500088	1.667	3.693879	0.183761
**CysK**	173	198	IAGVGTGGTLTGVSRYIKGTKGKTDL	2550.4185	6.438547	16.667002	4.478213	0.144377
**CysK**	173	198	IAGVGTGGTLTGVSRYIKGTKGKTDL	2550.4185	6.508809	60.000004	4.894271	0.177785
**CysK**	173	198	IAGVGTGGTLTGVSRYIKGTKGKTDL	2550.4185	6.499534	720.000061	5.000637	0.155769
**maxD control**	173	198	IAGVGTGGTLTGVSRYIKGTKGKTDL	2550.4185	6.43241	0	0	0
**maxD control**	173	198	IAGVGTGGTLTGVSRYIKGTKGKTDL	2550.4185	6.618968	720.000061	14.068477	0.416861
**CS complex**	183	200	TGVSRYIKGTKGKTDLIS	1924.0796	5.001148	0	0	0
**CS complex**	183	200	TGVSRYIKGTKGKTDLIS	1924.0796	5.038443	0.033	2.183547	0.099201
**CS complex**	183	200	TGVSRYIKGTKGKTDLIS	1924.0796	5.070461	1.667	3.535669	0.15168
**CS complex**	183	200	TGVSRYIKGTKGKTDLIS	1924.0796	5.054476	16.667002	4.200916	0.099691
**CS complex**	183	200	TGVSRYIKGTKGKTDLIS	1924.0796	5.073131	60.000004	4.731399	0.091377
**CS complex**	183	200	TGVSRYIKGTKGKTDLIS	1924.0796	5.076154	720.000061	4.729179	0.079749
**CysK**	183	200	TGVSRYIKGTKGKTDLIS	1924.0796	5.00288	0	0	0
**CysK**	183	200	TGVSRYIKGTKGKTDLIS	1924.0796	5.00241	0.033	1.886121	0.141712
**CysK**	183	200	TGVSRYIKGTKGKTDLIS	1924.0796	5.06742	1.667	3.495535	0.110885
**CysK**	183	200	TGVSRYIKGTKGKTDLIS	1924.0796	5.020628	16.667002	4.332554	0.096207
**CysK**	183	200	TGVSRYIKGTKGKTDLIS	1924.0796	5.080315	60.000004	4.650076	0.08392
**CysK**	183	200	TGVSRYIKGTKGKTDLIS	1924.0796	5.073737	720.000061	4.624872	0.088975
**maxD control**	183	200	TGVSRYIKGTKGKTDLIS	1924.0796	5.001148	0	0	0
**maxD control**	183	200	TGVSRYIKGTKGKTDLIS	1924.0796	5.253312	720.000061	9.523317	0.336878
**CS complex**	187	198	RYIKGTKGKTDL	1379.7941	3.48386	0	0	0
**CS complex**	187	198	RYIKGTKGKTDL	1379.7941	3.515411	0.033	1.68759	0.038457
**CS complex**	187	198	RYIKGTKGKTDL	1379.7941	3.546469	1.667	2.782158	0.01143
**CS complex**	187	198	RYIKGTKGKTDL	1379.7941	3.57041	60.000004	3.742936	0
**CS complex**	187	198	RYIKGTKGKTDL	1379.7941	3.573694	720.000061	3.727214	0
**CysK**	187	198	RYIKGTKGKTDL	1379.7941	3.476248	0	0	0
**CysK**	187	198	RYIKGTKGKTDL	1379.7941	3.500092	0.033	1.565303	0.123362
**CysK**	187	198	RYIKGTKGKTDL	1379.7941	3.5436	1.667	2.811616	0.027396
**CysK**	187	198	RYIKGTKGKTDL	1379.7941	3.54977	16.667002	3.657364	2.27E-13
**CysK**	187	198	RYIKGTKGKTDL	1379.7941	3.576992	60.000004	3.780198	2.27E-13
**CysK**	187	198	RYIKGTKGKTDL	1379.7941	3.572355	720.000061	3.756558	2.27E-13

**maxD control**	187	198	RYIKGTKGKTDL	1379.7941	3.48386	0	0	0
**maxD control**	187	198	RYIKGTKGKTDL	1379.7941	3.840144	720.000061	5.132507	0.07265
**CS complex**	187	200	RYIKGTKGKTDLIS	1579.91	4.070712	0	0	0
**CS complex**	187	200	RYIKGTKGKTDLIS	1579.91	4.10114	0.033	1.732	0.062542
**CS complex**	187	200	RYIKGTKGKTDLIS	1579.91	4.123961	1.667	3.003582	0.044034
**CS complex**	187	200	RYIKGTKGKTDLIS	1579.91	4.134231	60.000004	4.01733	0
**CS complex**	187	200	RYIKGTKGKTDLIS	1579.91	4.140042	720.000061	4.036793	0
**CysK**	187	200	RYIKGTKGKTDLIS	1579.91	4.070251	0	0	0
**CysK**	187	200	RYIKGTKGKTDLIS	1579.91	4.082694	0.033	1.609409	0.084096
**CysK**	187	200	RYIKGTKGKTDLIS	1579.91	4.119705	1.667	3.053231	0.040177
**CysK**	187	200	RYIKGTKGKTDLIS	1579.91	4.097424	16.667002	3.875659	0
**CysK**	187	200	RYIKGTKGKTDLIS	1579.91	4.145523	60.000004	4.086721	0
**CysK**	187	200	RYIKGTKGKTDLIS	1579.91	4.13677	720.000061	4.132339	0
**maxD control**	187	200	RYIKGTKGKTDLIS	1579.91	4.070712	0	0	0
**maxD control**	187	200	RYIKGTKGKTDLIS	1579.91	4.395772	720.000061	6.667636	0.095277
**CS complex**	199	212	ISVAVEPTDSPVIA	1397.7454	8.573763	0	0	0
**CS complex**	199	212	ISVAVEPTDSPVIA	1397.7454	8.619394	0.033	0.663701	0.035125
**CS complex**	199	212	ISVAVEPTDSPVIA	1397.7454	8.66566	1.667	1.771911	0.047034
**CS complex**	199	212	ISVAVEPTDSPVIA	1397.7454	8.570005	16.667002	2.388144	0
**CS complex**	199	212	ISVAVEPTDSPVIA	1397.7454	8.662475	60.000004	2.81192	0
**CS complex**	199	212	ISVAVEPTDSPVIA	1397.7454	8.667795	720.000061	3.66265	0
**CysK**	199	212	ISVAVEPTDSPVIA	1397.7454	8.568867	0	0	0
**CysK**	199	212	ISVAVEPTDSPVIA	1397.7454	8.567499	0.033	0.607353	0.027542
**CysK**	199	212	ISVAVEPTDSPVIA	1397.7454	8.663169	1.667	1.87731	0.061405
**CysK**	199	212	ISVAVEPTDSPVIA	1397.7454	8.577206	16.667002	2.437324	0
**CysK**	199	212	ISVAVEPTDSPVIA	1397.7454	8.669676	60.000004	3.001554	0
**CysK**	199	212	ISVAVEPTDSPVIA	1397.7454	8.663643	720.000061	4.144343	2.27E-13
**maxD control**	199	212	ISVAVEPTDSPVIA	1397.7454	8.573763	0	0	0
**maxD control**	199	212	ISVAVEPTDSPVIA	1397.7454	8.80759	720.000061	5.669265	0.112728
**CS complex**	201	212	VAVEPTDSPVIA	1197.6293	6.269046	0	0	0
**CS complex**	201	212	VAVEPTDSPVIA	1197.6293	6.295449	0.033	0.609547	0.042228
**CS complex**	201	212	VAVEPTDSPVIA	1197.6293	6.331092	1.667	0.706725	0.050528
**CS complex**	201	212	VAVEPTDSPVIA	1197.6293	6.26438	16.667002	0.762031	0.043557
**CS complex**	201	212	VAVEPTDSPVIA	1197.6293	6.331277	60.000004	0.952746	0.048055
**CS complex**	201	212	VAVEPTDSPVIA	1197.6293	6.333415	720.000061	1.491295	0.046518
**CysK**	201	212	VAVEPTDSPVIA	1197.6293	6.265387	0	0	0
**CysK**	201	212	VAVEPTDSPVIA	1197.6293	6.264657	0.033	0.568009	0.03939
**CysK**	201	212	VAVEPTDSPVIA	1197.6293	6.332818	1.667	0.734789	0.038832
**CysK**	201	212	VAVEPTDSPVIA	1197.6293	6.28356	16.667002	0.824669	0.028154
**CysK**	201	212	VAVEPTDSPVIA	1197.6293	6.340561	60.000004	0.968564	0.028595
**CysK**	201	212	VAVEPTDSPVIA	1197.6293	6.336587	720.000061	1.885395	0.062127

**maxD control**	201	212	VAVEPTDSPVIA	1197.6293	6.269046	0	0	0
**maxD control**	201	212	VAVEPTDSPVIA	1197.6293	6.402856	720.000061	5.171694	0.18122
**CS complex**	201	215	VAVEPTDSPVIAQAL	1509.8091	7.590754	0	0	0
**CS complex**	201	215	VAVEPTDSPVIAQAL	1509.8091	7.615755	0.033	0.786468	0.023088
**CS complex**	201	215	VAVEPTDSPVIAQAL	1509.8091	7.655544	1.667	1.046696	0.049317
**CS complex**	201	215	VAVEPTDSPVIAQAL	1509.8091	7.575479	16.667002	1.139255	0
**CS complex**	201	215	VAVEPTDSPVIAQAL	1509.8091	7.652748	60.000004	1.475694	0
**CS complex**	201	215	VAVEPTDSPVIAQAL	1509.8091	7.657271	720.000061	2.644224	0
**CysK**	201	215	VAVEPTDSPVIAQAL	1509.8091	7.58562	0	0	0
**CysK**	201	215	VAVEPTDSPVIAQAL	1509.8091	7.572609	0.033	0.579068	0.072851
**CysK**	201	215	VAVEPTDSPVIAQAL	1509.8091	7.654723	1.667	0.833816	0.050695
**CysK**	201	215	VAVEPTDSPVIAQAL	1509.8091	7.603635	16.667002	0.96671	2.27E-13
**CysK**	201	215	VAVEPTDSPVIAQAL	1509.8091	7.658827	60.000004	1.656104	0
**CysK**	201	215	VAVEPTDSPVIAQAL	1509.8091	7.644923	720.000061	3.736897	0
**maxD control**	201	215	VAVEPTDSPVIAQAL	1509.8091	7.590754	0	0	0
**maxD control**	201	215	VAVEPTDSPVIAQAL	1509.8091	7.70904	720.000061	7.197413	0.10815
**CS complex**	201	218	VAVEPTDSPVIAQALAGE	1766.9104	8.031617	0	0	0
**CS complex**	201	218	VAVEPTDSPVIAQALAGE	1766.9104	8.064873	0.033	0.987981	0.04177
**CS complex**	201	218	VAVEPTDSPVIAQALAGE	1766.9104	8.105565	1.667	1.721274	0.014702
**CS complex**	201	218	VAVEPTDSPVIAQALAGE	1766.9104	8.013295	16.667002	1.829287	0
**CS complex**	201	218	VAVEPTDSPVIAQALAGE	1766.9104	8.101911	60.000004	2.416565	0
**CS complex**	201	218	VAVEPTDSPVIAQALAGE	1766.9104	8.093921	720.000061	4.140594	0
**CysK**	201	218	VAVEPTDSPVIAQALAGE	1766.9104	8.023335	0	0	0
**CysK**	201	218	VAVEPTDSPVIAQALAGE	1766.9104	8.013158	0.033	0.904482	0.0351
**CysK**	201	218	VAVEPTDSPVIAQALAGE	1766.9104	8.104786	1.667	1.709422	0.041268
**CysK**	201	218	VAVEPTDSPVIAQALAGE	1766.9104	8.022311	16.667002	1.968321	2.27E-13
**CysK**	201	218	VAVEPTDSPVIAQALAGE	1766.9104	8.103263	60.000004	2.844235	2.27E-13
**CysK**	201	218	VAVEPTDSPVIAQALAGE	1766.9104	8.089045	720.000061	5.616169	2.27E-13
**maxD control**	201	218	VAVEPTDSPVIAQALAGE	1766.9104	8.031617	0	0	0
**maxD control**	201	218	VAVEPTDSPVIAQALAGE	1766.9104	8.123513	720.000061	8.567887	0.227492
**CS complex**	205	212	PTDSPVIA	799.41961	6.280359	0	0	0
**CS complex**	205	212	PTDSPVIA	799.41961	6.308172	0.033	0.392411	0.018014
**CS complex**	205	212	PTDSPVIA	799.41961	6.34179	1.667	0.519378	0.027284
**CS complex**	205	212	PTDSPVIA	799.41961	6.26874	16.667002	0.422337	0
**CS complex**	205	212	PTDSPVIA	799.41961	6.33739	60.000004	0.742369	0
**CS complex**	205	212	PTDSPVIA	799.41961	6.344547	720.000061	1.073993	0
**CysK**	205	212	PTDSPVIA	799.41961	6.02023	0	0	0
**CysK**	205	212	PTDSPVIA	799.41961	6.022166	0.033	0.310712	0.024538
**CysK**	205	212	PTDSPVIA	799.41961	6.337454	1.667	0.485934	0.046132
**CysK**	205	212	PTDSPVIA	799.41961	6.286885	16.667002	0.506023	0
**CysK**	205	212	PTDSPVIA	799.41961	6.344664	60.000004	0.665523	0

**CysK**	205	212	PTDSPVIA	799.41961	6.33869	720.000061	1.357978	0
**maxD control**	205	212	PTDSPVIA	799.41961	6.280359	0	0	0
**maxD control**	205	212	PTDSPVIA	799.41961	6.160476	720.000061	3.291867	0.153053
**CS complex**	213	239	QALAGEEIKPGPHKIQGIGAGFIPANL	2726.4924	7.639173	0	0	0
**CS complex**	213	239	QALAGEEIKPGPHKIQGIGAGFIPANL	2726.4924	7.681355	0.033	2.628491	0.067608
**CS complex**	213	239	QALAGEEIKPGPHKIQGIGAGFIPANL	2726.4924	7.725274	1.667	4.985658	0.091798
**CS complex**	213	239	QALAGEEIKPGPHKIQGIGAGFIPANL	2726.4924	7.625256	16.667002	7.186084	0.061298
**CS complex**	213	239	QALAGEEIKPGPHKIQGIGAGFIPANL	2726.4924	7.725592	60.000004	8.749569	0.046968
**CS complex**	213	239	QALAGEEIKPGPHKIQGIGAGFIPANL	2726.4924	7.726224	720.000061	10.999544	0.047078
**CysK**	213	239	QALAGEEIKPGPHKIQGIGAGFIPANL	2726.4924	7.636166	0	0	0
**CysK**	213	239	QALAGEEIKPGPHKIQGIGAGFIPANL	2726.4924	7.631011	0.033	3.04032	0.130343
**CysK**	213	239	QALAGEEIKPGPHKIQGIGAGFIPANL	2726.4924	7.723146	1.667	7.194401	0.192877
**CysK**	213	239	QALAGEEIKPGPHKIQGIGAGFIPANL	2726.4924	7.636612	16.667002	9.133606	0.048829
**CysK**	213	239	QALAGEEIKPGPHKIQGIGAGFIPANL	2726.4924	7.730754	60.000004	10.489397	0.058774
**CysK**	213	239	QALAGEEIKPGPHKIQGIGAGFIPANL	2726.4924	7.726318	720.000061	11.452598	0.048224
**maxD control**	213	239	QALAGEEIKPGPHKIQGIGAGFIPANL	2726.4924	7.639173	0	0	0
**maxD control**	213	239	QALAGEEIKPGPHKIQGIGAGFIPANL	2726.4924	7.885022	720.000061	11.540015	0.351245
**CS complex**	214	239	ALAGEEIKPGPHKIQGIGAGFIPANL	2598.4338	7.653371	0	0	0
**CS complex**	214	239	ALAGEEIKPGPHKIQGIGAGFIPANL	2598.4338	7.695626	0.033	2.580241	0.1004
**CS complex**	214	239	ALAGEEIKPGPHKIQGIGAGFIPANL	2598.4338	7.738664	1.667	4.772742	0.048137
**CS complex**	214	239	ALAGEEIKPGPHKIQGIGAGFIPANL	2598.4338	7.636521	16.667002	7.101731	0
**CS complex**	214	239	ALAGEEIKPGPHKIQGIGAGFIPANL	2598.4338	7.740537	60.000004	8.570164	0
**CS complex**	214	239	ALAGEEIKPGPHKIQGIGAGFIPANL	2598.4338	7.741604	720.000061	10.235991	0
**CysK**	214	239	ALAGEEIKPGPHKIQGIGAGFIPANL	2598.4338	7.648797	0	0	0
**CysK**	214	239	ALAGEEIKPGPHKIQGIGAGFIPANL	2598.4338	7.645589	0.033	2.84049	0.103937
**CysK**	214	239	ALAGEEIKPGPHKIQGIGAGFIPANL	2598.4338	7.737773	1.667	6.989079	0.1288
**CysK**	214	239	ALAGEEIKPGPHKIQGIGAGFIPANL	2598.4338	7.650651	16.667002	8.721506	0
**CysK**	214	239	ALAGEEIKPGPHKIQGIGAGFIPANL	2598.4338	7.744307	60.000004	10.071431	0
**CysK**	214	239	ALAGEEIKPGPHKIQGIGAGFIPANL	2598.4338	7.738017	720.000061	10.628494	0
**maxD control**	214	239	ALAGEEIKPGPHKIQGIGAGFIPANL	2598.4338	7.653371	0	0	0
**maxD control**	214	239	ALAGEEIKPGPHKIQGIGAGFIPANL	2598.4338	7.905569	720.000061	11.014211	0.351049
**CS complex**	216	239	AGEEIKPGPHKIQGIGAGFIPANL	2414.3127	7.564093	0	0	0
**CS complex**	216	239	AGEEIKPGPHKIQGIGAGFIPANL	2414.3127	7.606784	0.033	2.569573	0.075232
**CS complex**	216	239	AGEEIKPGPHKIQGIGAGFIPANL	2414.3127	7.645397	1.667	4.316202	0.072351
**CS complex**	216	239	AGEEIKPGPHKIQGIGAGFIPANL	2414.3127	7.55353	16.667002	6.342729	0.037249
**CS complex**	216	239	AGEEIKPGPHKIQGIGAGFIPANL	2414.3127	7.648335	60.000004	7.788967	0.070251
**CS complex**	216	239	AGEEIKPGPHKIQGIGAGFIPANL	2414.3127	7.650412	720.000061	9.236289	0.030272
**CysK**	216	239	AGEEIKPGPHKIQGIGAGFIPANL	2414.3127	7.564965	0	0	0
**CysK**	216	239	AGEEIKPGPHKIQGIGAGFIPANL	2414.3127	7.561249	0.033	2.842684	0.112439
**CysK**	216	239	AGEEIKPGPHKIQGIGAGFIPANL	2414.3127	7.644449	1.667	6.485386	0.124676
**CysK**	216	239	AGEEIKPGPHKIQGIGAGFIPANL	2414.3127	7.565643	16.667002	8.158782	0.037456

**CysK**	216	239	AGEEIKPGPHKIQGIGAGFIPANL	2414.3127	7.649708	60.000004	9.102044	0.025854
**CysK**	216	239	AGEEIKPGPHKIQGIGAGFIPANL	2414.3127	7.643812	720.000061	9.522241	0.04525
**maxD control**	216	239	AGEEIKPGPHKIQGIGAGFIPANL	2414.3127	7.564093	0	0	0
**maxD control**	216	239	AGEEIKPGPHKIQGIGAGFIPANL	2414.3127	7.820196	720.000061	9.751254	0.30912
**CS complex**	218	239	EEIKPGPHKIQGIGAGFIPANL	2286.2542	7.571092	0	0	0
**CS complex**	218	239	EEIKPGPHKIQGIGAGFIPANL	2286.2542	7.61162	0.033	2.097655	0.087618
**CS complex**	218	239	EEIKPGPHKIQGIGAGFIPANL	2286.2542	7.651271	1.667	3.46711	0.079834
**CS complex**	218	239	EEIKPGPHKIQGIGAGFIPANL	2286.2542	7.568881	16.667002	5.407313	0.014255
**CS complex**	218	239	EEIKPGPHKIQGIGAGFIPANL	2286.2542	7.654011	60.000004	6.728787	0.036346
**CS complex**	218	239	EEIKPGPHKIQGIGAGFIPANL	2286.2542	7.655126	720.000061	8.099738	0.071983
**CysK**	218	239	EEIKPGPHKIQGIGAGFIPANL	2286.2542	7.576107	0	0	0
**CysK**	218	239	EEIKPGPHKIQGIGAGFIPANL	2286.2542	7.565324	0.033	2.445768	0.096496
**CysK**	218	239	EEIKPGPHKIQGIGAGFIPANL	2286.2542	7.651185	1.667	5.464326	0.140412
**CysK**	218	239	EEIKPGPHKIQGIGAGFIPANL	2286.2542	7.571615	16.667002	7.036569	0.067502
**CysK**	218	239	EEIKPGPHKIQGIGAGFIPANL	2286.2542	7.65989	60.000004	7.96776	0.036135
**CysK**	218	239	EEIKPGPHKIQGIGAGFIPANL	2286.2542	7.650854	720.000061	8.450548	0.021957
**maxD control**	218	239	EEIKPGPHKIQGIGAGFIPANL	2286.2542	7.571092	0	0	0
**maxD control**	218	239	EEIKPGPHKIQGIGAGFIPANL	2286.2542	7.826287	720.000061	8.886493	0.282137
**CS complex**	240	253	DLKLVDKVIGITNE	1556.8828	7.94664	0	0	0
**CS complex**	240	253	DLKLVDKVIGITNE	1556.8828	7.978323	0.033	1.274182	0.058181
**CS complex**	240	253	DLKLVDKVIGITNE	1556.8828	8.001755	1.667	3.488198	0.074595
**CS complex**	240	253	DLKLVDKVIGITNE	1556.8828	7.916045	16.667002	4.229836	0.047891
**CS complex**	240	253	DLKLVDKVIGITNE	1556.8828	7.999364	60.000004	4.432672	0.059763
**CS complex**	240	253	DLKLVDKVIGITNE	1556.8828	7.992664	720.000061	5.174583	0.045798
**CysK**	240	253	DLKLVDKVIGITNE	1556.8828	7.940465	0	0	0
**CysK**	240	253	DLKLVDKVIGITNE	1556.8828	7.934657	0.033	1.173499	0.101774
**CysK**	240	253	DLKLVDKVIGITNE	1556.8828	7.999542	1.667	3.599	0.06388
**CysK**	240	253	DLKLVDKVIGITNE	1556.8828	7.927516	16.667002	4.270594	0.037581
**CysK**	240	253	DLKLVDKVIGITNE	1556.8828	8.005266	60.000004	4.517905	0.067172
**CysK**	240	253	DLKLVDKVIGITNE	1556.8828	7.988209	720.000061	5.414699	0.054046
**maxD control**	240	253	DLKLVDKVIGITNE	1556.8828	7.94664	0	0	0
**maxD control**	240	253	DLKLVDKVIGITNE	1556.8828	8.06409	720.000061	8.062396	0.179747
**CS complex**	242	253	KLVDKVIGITNE	1328.7717	6.013695	0	0	0
**CS complex**	242	253	KLVDKVIGITNE	1328.7717	6.047043	0.033	1.214359	0.050429
**CS complex**	242	253	KLVDKVIGITNE	1328.7717	6.076216	1.667	3.039596	0.02989
**CS complex**	242	253	KLVDKVIGITNE	1328.7717	5.99892	16.667002	3.793849	0
**CS complex**	242	253	KLVDKVIGITNE	1328.7717	6.077202	60.000004	3.862274	0
**CS complex**	242	253	KLVDKVIGITNE	1328.7717	6.076584	720.000061	4.078825	0
**CysK**	242	253	KLVDKVIGITNE	1328.7717	6.006989	0	0	0
**CysK**	242	253	KLVDKVIGITNE	1328.7717	6.007157	0.033	1.072407	0.097537
**CysK**	242	253	KLVDKVIGITNE	1328.7717	6.073552	1.667	3.079164	0.04632

**CysK**	242	253	KLVDKVIGITNE	1328.7717	6.011471	16.667002	3.720717	0
**CysK**	242	253	KLVDKVIGITNE	1328.7717	6.08034	60.000004	3.81827	0
**CysK**	242	253	KLVDKVIGITNE	1328.7717	6.07336	720.000061	4.09958	0
**maxD control**	242	253	KLVDKVIGITNE	1328.7717	6.013695	0	0	0
**maxD control**	242	253	KLVDKVIGITNE	1328.7717	6.197224	720.000061	6.767417	0.133539
**CS complex**	244	253	VDKVIGITNE	1087.5927	5.661108	0	0	0
**CS complex**	244	253	VDKVIGITNE	1087.5927	5.692141	0.033	0.873712	0.17382
**CS complex**	244	253	VDKVIGITNE	1087.5927	5.720864	1.667	2.537555	0.155294
**CS complex**	244	253	VDKVIGITNE	1087.5927	5.649805	16.667002	3.164922	0.152351
**CS complex**	244	253	VDKVIGITNE	1087.5927	5.724822	60.000004	3.216298	0.154304
**CS complex**	244	253	VDKVIGITNE	1087.5927	5.7269	720.000061	3.209365	0.155121
**CysK**	244	253	VDKVIGITNE	1087.5927	5.656466	0	0	0
**CysK**	244	253	VDKVIGITNE	1087.5927	5.661718	0.033	0.811139	0.162898
**CysK**	244	253	VDKVIGITNE	1087.5927	5.719027	1.667	2.612324	0.136054
**CysK**	244	253	VDKVIGITNE	1087.5927	5.671894	16.667002	3.193252	0.118548
**CysK**	244	253	VDKVIGITNE	1087.5927	5.730117	60.000004	3.257288	0.117273
**CysK**	244	253	VDKVIGITNE	1087.5927	5.724387	720.000061	3.246906	0.116839
**maxD control**	244	253	VDKVIGITNE	1087.5927	5.661108	0	0	0
**maxD control**	244	253	VDKVIGITNE	1087.5927	5.845542	720.000061	4.634232	0.185672
**CS complex**	244	255	VDKVIGITNEEA	1287.6724	5.810719	0	0	0
**CS complex**	244	255	VDKVIGITNEEA	1287.6724	5.841245	0.033	1.069503	0.035393
**CS complex**	244	255	VDKVIGITNEEA	1287.6724	5.867655	1.667	3.330247	0.01895
**CS complex**	244	255	VDKVIGITNEEA	1287.6724	5.800646	16.667002	4.028255	0
**CS complex**	244	255	VDKVIGITNEEA	1287.6724	5.867734	60.000004	4.106501	0
**CS complex**	244	255	VDKVIGITNEEA	1287.6724	5.85653	720.000061	4.090659	0
**CysK**	244	255	VDKVIGITNEEA	1287.6724	5.804723	0	0	0
**CysK**	244	255	VDKVIGITNEEA	1287.6724	5.805382	0.033	0.982309	0.075098
**CysK**	244	255	VDKVIGITNEEA	1287.6724	5.866773	1.667	3.507099	0.045809
**CysK**	244	255	VDKVIGITNEEA	1287.6724	5.814458	16.667002	4.055693	0
**CysK**	244	255	VDKVIGITNEEA	1287.6724	5.877366	60.000004	4.131251	0
**CysK**	244	255	VDKVIGITNEEA	1287.6724	5.869903	720.000061	4.200084	0
**maxD control**	244	255	VDKVIGITNEEA	1287.6724	5.810719	0	0	0
**maxD control**	244	255	VDKVIGITNEEA	1287.6724	5.988842	720.000061	6.189191	0.109869
**CS complex**	254	269	EAISTARRLMEEEGIL	1817.9359	7.829728	0	0	0
**CS complex**	254	269	EAISTARRLMEEEGIL	1817.9359	7.873368	0.033	0.19509	0.149934
**CS complex**	254	269	EAISTARRLMEEEGIL	1817.9359	7.916061	1.667	0.36126	0.162242
**CS complex**	254	269	EAISTARRLMEEEGIL	1817.9359	7.820503	16.667002	1.146479	0.126252
**CS complex**	254	269	EAISTARRLMEEEGIL	1817.9359	7.912112	60.000004	1.822609	0.125255
**CS complex**	254	269	EAISTARRLMEEEGIL	1817.9359	7.912063	720.000061	2.228045	0.159917
**CysK**	254	269	EAISTARRLMEEEGIL	1817.9359	7.82732	0	0	0
**CysK**	254	269	EAISTARRLMEEEGIL	1817.9359	7.825089	0.033	0.202956	0.118575

**CysK**	254	269	EAISTARRLMEEEGIL	1817.9359	7.913305	1.667	0.439212	0.134324
**CysK**	254	269	EAISTARRLMEEEGIL	1817.9359	7.831537	16.667002	1.189522	0.114372
**CysK**	254	269	EAISTARRLMEEEGIL	1817.9359	7.920736	60.000004	1.71146	0.101433
**CysK**	254	269	EAISTARRLMEEEGIL	1817.9359	7.911229	720.000061	2.209359	0.09408
**maxD control**	254	269	EAISTARRLMEEEGIL	1817.9359	7.829737	0	0	0
**maxD control**	254	269	EAISTARRLMEEEGIL	1817.9359	8.024908	720.000061	10.585419	0.163797
**CS complex**	259	264	ARRLME	775.4177	4.035339	0	0	0
**CS complex**	259	264	ARRLME	775.4177	4.067773	0.033	0.190617	0.010292
**CS complex**	259	264	ARRLME	775.4177	4.090513	1.667	0.164062	0.025681
**CS complex**	259	264	ARRLME	775.4177	4.109698	60.000004	0.193145	0
**CS complex**	259	264	ARRLME	775.4177	4.110861	720.000061	0.191283	0
**CysK**	259	264	ARRLME	775.4177	4.02981	0	0	0
**CysK**	259	264	ARRLME	775.4177	4.059463	0.033	0.238497	0.015192
**CysK**	259	264	ARRLME	775.4177	4.092667	1.667	0.257564	0.014772
**CysK**	259	264	ARRLME	775.4177	4.077725	16.667002	0.199102	0
**CysK**	259	264	ARRLME	775.4177	4.1176	60.000004	0.244421	0
**CysK**	259	264	ARRLME	775.4177	4.109997	720.000061	0.238224	0
**maxD control**	259	264	ARRLME	775.4177	4.035339	0	0	0
**maxD control**	259	264	ARRLME	775.4177	4.338785	720.000061	2.642323	0.007412
**CS complex**	259	269	ARRLMEEEGIL	1316.6924	6.804604	0	0	0
**CS complex**	259	269	ARRLMEEEGIL	1316.6924	6.844976	0.033	0.16718	0.031558
**CS complex**	259	269	ARRLMEEEGIL	1316.6924	6.884031	1.667	0.242445	0.043072
**CS complex**	259	269	ARRLMEEEGIL	1316.6924	6.79779	16.667002	0.427009	0
**CS complex**	259	269	ARRLMEEEGIL	1316.6924	6.886458	60.000004	0.59769	0
**CS complex**	259	269	ARRLMEEEGIL	1316.6924	6.88435	720.000061	0.739488	0
**CysK**	259	269	ARRLMEEEGIL	1316.6924	6.8642	0	0	0
**CysK**	259	269	ARRLMEEEGIL	1316.6924	6.800211	0.033	0.218571	0.016938
**CysK**	259	269	ARRLMEEEGIL	1316.6924	6.883971	1.667	0.322836	0.037567
**CysK**	259	269	ARRLMEEEGIL	1316.6924	6.810309	16.667002	0.566771	0
**CysK**	259	269	ARRLMEEEGIL	1316.6924	6.894001	60.000004	0.654277	0
**CysK**	259	269	ARRLMEEEGIL	1316.6924	6.888299	720.000061	0.672252	0
**maxD control**	259	269	ARRLMEEEGIL	1316.6924	6.804604	0	0	0
**maxD control**	259	269	ARRLMEEEGIL	1316.6924	7.087483	720.000061	4.879656	0.071993
**CS complex**	270	281	AGISSGAAVAAA	945.4935	5.222746	0	0	0
**CS complex**	270	281	AGISSGAAVAAA	945.4935	5.248292	0.033	0.857998	0.03881
**CS complex**	270	281	AGISSGAAVAAA	945.4935	5.269571	1.667	2.401059	0.026341
**CS complex**	270	281	AGISSGAAVAAA	945.4935	5.190423	16.667002	2.501781	0
**CS complex**	270	281	AGISSGAAVAAA	945.4935	5.275149	60.000004	2.51184	0
**CS complex**	270	281	AGISSGAAVAAA	945.4935	5.277642	720.000061	2.742031	0
**CysK**	270	281	AGISSGAAVAAA	945.4935	5.20793	0	0	0
**CysK**	270	281	AGISSGAAVAAA	945.4935	5.216927	0.033	0.758204	0.102303

**CysK**	270	281	AGISSGAAVAAA	945.4935	5.26505	1.667	2.165656	0.010556
**CysK**	270	281	AGISSGAAVAAA	945.4935	5.232127	16.667002	2.280877	0
**CysK**	270	281	AGISSGAAVAAA	945.4935	5.27927	60.000004	2.326559	0
**CysK**	270	281	AGISSGAAVAAA	945.4935	5.272304	720.000061	2.409555	0
**maxD control**	270	281	AGISSGAAVAAA	945.4935	5.222746	0	0	0
**maxD control**	270	281	AGISSGAAVAAA	945.4935	5.323161	720.000061	6.702436	0.15242
**CS complex**	271	280	GISSGAAVAA	803.4193	5.698179	0	0	0
**CS complex**	271	280	GISSGAAVAA	803.4193	5.725926	0.033	0.206187	0.00462
**CS complex**	271	280	GISSGAAVAA	803.4193	5.756173	1.667	0.403145	0.012628
**CS complex**	271	280	GISSGAAVAA	803.4193	5.694621	16.667002	0.554009	0
**CS complex**	271	280	GISSGAAVAA	803.4193	5.758574	60.000004	0.669553	0
**CS complex**	271	280	GISSGAAVAA	803.4193	5.765335	720.000061	0.602596	0
**CysK**	271	280	GISSGAAVAA	803.4193	5.693846	0	0	0
**CysK**	271	280	GISSGAAVAA	803.4193	5.698103	0.033	0.179112	0.014831
**CysK**	271	280	GISSGAAVAA	803.4193	5.755382	1.667	0.464478	0.030124
**CysK**	271	280	GISSGAAVAA	803.4193	5.714895	16.667002	0.701638	0
**CysK**	271	280	GISSGAAVAA	803.4193	5.767945	60.000004	0.766852	0
**CysK**	271	280	GISSGAAVAA	803.4193	5.76085	720.000061	0.681437	0
**maxD control**	271	280	GISSGAAVAA	803.4193	5.698179	0	0	0
**maxD control**	271	280	GISSGAAVAA	803.4193	5.829607	720.000061	1.189289	0.047269
**CS complex**	280	289	AALKLQEDES	1103.5514	5.137721	0	0	0
**CS complex**	280	289	AALKLQEDES	1103.5514	5.166834	0.033	1.328961	0.054847
**CS complex**	280	289	AALKLQEDES	1103.5514	5.195197	1.667	2.024471	0.144705
**CS complex**	280	289	AALKLQEDES	1103.5514	5.135173	16.667002	2.058167	0
**CS complex**	280	289	AALKLQEDES	1103.5514	5.202518	60.000004	2.7247	0
**CS complex**	280	289	AALKLQEDES	1103.5514	5.201027	720.000061	3.403749	0
**CysK**	280	289	AALKLQEDES	1103.5514	5.13413	0	0	0
**CysK**	280	289	AALKLQEDES	1103.5514	5.138328	0.033	1.208887	0.046134
**CysK**	280	289	AALKLQEDES	1103.5514	5.194553	1.667	1.973394	0.142693
**CysK**	280	289	AALKLQEDES	1103.5514	5.15803	16.667002	1.937335	0
**CysK**	280	289	AALKLQEDES	1103.5514	5.208915	60.000004	2.67412	0
**CysK**	280	289	AALKLQEDES	1103.5514	5.198874	720.000061	3.192659	0
**maxD control**	280	289	AALKLQEDES	1103.5514	5.137721	0	0	0
**maxD control**	280	289	AALKLQEDES	1103.5514	5.324894	720.000061	4.862109	0.095439
**CS complex**	282	289	LKLQEDES	961.4772	4.764318	0	0	0
**CS complex**	282	289	LKLQEDES	961.4772	4.795054	0.033	1.191129	0.040094
**CS complex**	282	289	LKLQEDES	961.4772	4.818046	1.667	1.790052	0.029291
**CS complex**	282	289	LKLQEDES	961.4772	4.78708	16.667002	2.328162	1.14E-13
**CS complex**	282	289	LKLQEDES	961.4772	4.827198	60.000004	2.608523	0
**CS complex**	282	289	LKLQEDES	961.4772	4.826918	720.000061	2.894554	0
**CysK**	282	289	LKLQEDES	961.4772	4.757948	0	0	0

**CysK**	282	289	LKLQEDES	961.4772	4.771145	0.033	1.185939	0.045286
**CysK**	282	289	LKLQEDES	961.4772	4.819308	1.667	1.788637	0.072766
**CysK**	282	289	LKLQEDES	961.4772	4.786697	16.667002	2.205418	1.14E-13
**CysK**	282	289	LKLQEDES	961.4772	4.833426	60.000004	2.579392	0
**CysK**	282	289	LKLQEDES	961.4772	4.824578	720.000061	2.808108	0
**maxD control**	282	289	LKLQEDES	961.4772	4.764318	0	0	0
**maxD control**	282	289	LKLQEDES	961.4772	4.960623	720.000061	3.333168	0.082476
**CS complex**	290	296	FTNKNIV	835.4605	5.465073	0	0	0
**CS complex**	290	296	FTNKNIV	835.4605	5.502586	0.033	0.605104	0.154912
**CS complex**	290	296	FTNKNIV	835.4605	5.533825	1.667	1.303056	0.129375
**CS complex**	290	296	FTNKNIV	835.4605	5.454679	16.667002	2.1824	0.166363
**CS complex**	290	296	FTNKNIV	835.4605	5.539279	60.000004	2.352663	0.118741
**CS complex**	290	296	FTNKNIV	835.4605	5.542144	720.000061	2.378057	0.118906
**CysK**	290	296	FTNKNIV	835.4605	5.462091	0	0	0
**CysK**	290	296	FTNKNIV	835.4605	5.470035	0.033	0.599593	0.155317
**CysK**	290	296	FTNKNIV	835.4605	5.533174	1.667	1.360068	0.134961
**CysK**	290	296	FTNKNIV	835.4605	5.482056	16.667002	2.015356	0.127271
**CysK**	290	296	FTNKNIV	835.4605	5.552545	60.000004	2.437283	0.128429
**CysK**	290	296	FTNKNIV	835.4605	5.541482	720.000061	2.446066	0.129626
**maxD control**	290	296	FTNKNIV	835.4605	5.465073	0	0	0
**maxD control**	290	296	FTNKNIV	835.4605	5.691566	720.000061	3.033328	0.121237
**CS complex**	290	299	FTNKNIVVIL	1160.6969	8.029482	0	0	0
**CS complex**	290	299	FTNKNIVVIL	1160.6969	8.072215	0.033	0.445379	0.017117
**CS complex**	290	299	FTNKNIVVIL	1160.6969	8.120275	1.667	1.11894	0.026353
**CS complex**	290	299	FTNKNIVVIL	1160.6969	8.016898	16.667002	1.759752	0
**CS complex**	290	299	FTNKNIVVIL	1160.6969	8.117874	60.000004	2.256265	0
**CS complex**	290	299	FTNKNIVVIL	1160.6969	8.120653	720.000061	2.344172	0
**CysK**	290	299	FTNKNIVVIL	1160.6969	8.02043	0	0	0
**CysK**	290	299	FTNKNIVVIL	1160.6969	8.013094	0.033	0.448994	0.01144
**CysK**	290	299	FTNKNIVVIL	1160.6969	8.117788	1.667	1.176101	0.024133
**CysK**	290	299	FTNKNIVVIL	1160.6969	8.030892	16.667002	1.791053	0
**CysK**	290	299	FTNKNIVVIL	1160.6969	8.124357	60.000004	2.313928	0
**CysK**	290	299	FTNKNIVVIL	1160.6969	8.121809	720.000061	2.297768	0
**maxD control**	290	299	FTNKNIVVIL	1160.6969	8.029482	0	0	0
**maxD control**	290	299	FTNKNIVVIL	1160.6969	8.267704	720.000061	5.374023	0.109007
**CS complex**	295	306	IVVILPSSGERY	1332.7454	7.20073	0	0	0
**CS complex**	295	306	IVVILPSSGERY	1332.7454	7.236058	0.033	0.680264	0.016443
**CS complex**	295	306	IVVILPSSGERY	1332.7454	7.279593	1.667	1.917055	0.037372
**CS complex**	295	306	IVVILPSSGERY	1332.7454	7.179027	16.667002	2.97086	0
**CS complex**	295	306	IVVILPSSGERY	1332.7454	7.280355	60.000004	3.252648	0
**CS complex**	295	306	IVVILPSSGERY	1332.7454	7.284604	720.000061	3.225091	0

**CysK**	295	306	IVVILPSSGERY	1332.7454	7.191671	0	0	0
**CysK**	295	306	IVVILPSSGERY	1332.7454	7.19262	0.033	0.740081	0.01463
**CysK**	295	306	IVVILPSSGERY	1332.7454	7.276885	1.667	2.277625	0.06871
**CysK**	295	306	IVVILPSSGERY	1332.7454	7.204357	16.667002	3.232205	0
**CysK**	295	306	IVVILPSSGERY	1332.7454	7.285907	60.000004	3.349601	0
**CysK**	295	306	IVVILPSSGERY	1332.7454	7.279194	720.000061	3.321142	0
**maxD control**	295	306	IVVILPSSGERY	1332.7454	7.20073	0	0	0
**maxD control**	295	306	IVVILPSSGERY	1332.7454	7.395128	720.000061	5.24812	0.064597
**CS complex**	295	311	IVVILPSSGERYLSTAL	1818.0303	8.463657	0	0	0
**CS complex**	295	311	IVVILPSSGERYLSTAL	1818.0303	8.505435	0.033	2.540011	0.239793
**CS complex**	295	311	IVVILPSSGERYLSTAL	1818.0303	8.550966	1.667	5.024053	0.408072
**CS complex**	295	311	IVVILPSSGERYLSTAL	1818.0303	8.434744	16.667002	6.094022	0.360369
**CS complex**	295	311	IVVILPSSGERYLSTAL	1818.0303	8.535913	60.000004	6.467336	0.429186
**CS complex**	295	311	IVVILPSSGERYLSTAL	1818.0303	8.548895	720.000061	6.398605	0.476919
**CysK**	295	311	IVVILPSSGERYLSTAL	1818.0303	8.456211	0	0	0
**CysK**	295	311	IVVILPSSGERYLSTAL	1818.0303	8.448702	0.033	2.521846	0.251034
**CysK**	295	311	IVVILPSSGERYLSTAL	1818.0303	8.54503	1.667	5.359723	0.391556
**CysK**	295	311	IVVILPSSGERYLSTAL	1818.0303	8.450139	16.667002	6.346497	0.423023
**CysK**	295	311	IVVILPSSGERYLSTAL	1818.0303	8.548217	60.000004	6.563347	0.462969
**CysK**	295	311	IVVILPSSGERYLSTAL	1818.0303	8.541868	720.000061	6.459621	0.458888
**maxD control**	295	311	IVVILPSSGERYLSTAL	1818.0303	8.463657	0	0	0
**maxD control**	295	311	IVVILPSSGERYLSTAL	1818.0303	8.645617	720.000061	8.717647	0.45012
**CS complex**	297	308	VILPSSGERYLS	1320.709	6.600675	0	0	0
**CS complex**	297	308	VILPSSGERYLS	1320.709	6.632965	0.033	1.999386	0.064919
**CS complex**	297	308	VILPSSGERYLS	1320.709	6.665191	1.667	3.365117	0.103647
**CS complex**	297	308	VILPSSGERYLS	1320.709	6.588786	16.667002	4.391384	0
**CS complex**	297	308	VILPSSGERYLS	1320.709	6.666493	60.000004	4.636302	0
**CS complex**	297	308	VILPSSGERYLS	1320.709	6.673475	720.000061	4.569793	0
**CysK**	297	308	VILPSSGERYLS	1320.709	6.595341	0	0	0
**CysK**	297	308	VILPSSGERYLS	1320.709	6.591931	0.033	1.962184	0.079685
**CysK**	297	308	VILPSSGERYLS	1320.709	6.665619	1.667	3.715271	0.044896
**CysK**	297	308	VILPSSGERYLS	1320.709	6.606721	16.667002	4.751301	0
**CysK**	297	308	VILPSSGERYLS	1320.709	6.679042	60.000004	4.945836	0
**CysK**	297	308	VILPSSGERYLS	1320.709	6.667364	720.000061	4.788283	0
**maxD control**	297	308	VILPSSGERYLS	1320.709	6.600675	0	0	0
**maxD control**	297	308	VILPSSGERYLS	1320.709	6.77718	720.000061	5.386761	0.117698
**CS complex**	297	309	VILPSSGERYLST	1421.7566	6.606465	0	0	0
**CS complex**	297	309	VILPSSGERYLST	1421.7566	6.640994	0.033	1.860273	0.063423
**CS complex**	297	309	VILPSSGERYLST	1421.7566	6.673521	1.667	3.849073	0.034669
**CS complex**	297	309	VILPSSGERYLST	1421.7566	6.593467	16.667002	4.935157	0
**CS complex**	297	309	VILPSSGERYLST	1421.7566	6.675492	60.000004	5.339161	2.27E-13

**CS complex**	297	309	VILPSSGERYLST	1421.7566	6.676992	720.000061	5.246094	0
**CysK**	297	309	VILPSSGERYLST	1421.7566	6.601631	0	0	0
**CysK**	297	309	VILPSSGERYLST	1421.7566	6.601788	0.033	1.763246	0.086327
**CysK**	297	309	VILPSSGERYLST	1421.7566	6.67424	1.667	4.119266	0.099333
**CysK**	297	309	VILPSSGERYLST	1421.7566	6.608262	16.667002	5.151943	0
**CysK**	297	309	VILPSSGERYLST	1421.7566	6.682068	60.000004	5.321602	0
**CysK**	297	309	VILPSSGERYLST	1421.7566	6.675711	720.000061	5.21759	0
**maxD control**	297	309	VILPSSGERYLST	1421.7566	6.606465	0	0	0
**maxD control**	297	309	VILPSSGERYLST	1421.7566	6.784778	720.000061	5.888419	0.112925
**CS complex**	297	311	VILPSSGERYLSTAL	1605.8778	7.809882	0	0	0
**CS complex**	297	311	VILPSSGERYLSTAL	1605.8778	7.845407	0.033	2.081444	0.024094
**CS complex**	297	311	VILPSSGERYLSTAL	1605.8778	7.879889	1.667	4.214525	0.05234
**CS complex**	297	311	VILPSSGERYLSTAL	1605.8778	7.778575	16.667002	5.363952	0
**CS complex**	297	311	VILPSSGERYLSTAL	1605.8778	7.876192	60.000004	5.552884	0
**CS complex**	297	311	VILPSSGERYLSTAL	1605.8778	7.876063	720.000061	5.51341	0
**CysK**	297	311	VILPSSGERYLSTAL	1605.8778	7.802691	0	0	0
**CysK**	297	311	VILPSSGERYLSTAL	1605.8778	7.79446	0.033	2.060059	0.113006
**CysK**	297	311	VILPSSGERYLSTAL	1605.8778	7.876452	1.667	4.516406	0.02151
**CysK**	297	311	VILPSSGERYLSTAL	1605.8778	7.791706	16.667002	5.455642	0
**CysK**	297	311	VILPSSGERYLSTAL	1605.8778	7.881896	60.000004	5.609252	0
**CysK**	297	311	VILPSSGERYLSTAL	1605.8778	7.873906	720.000061	5.449268	0
**maxD control**	297	311	VILPSSGERYLSTAL	1605.8778	7.809882	0	0	0
**maxD control**	297	311	VILPSSGERYLSTAL	1605.8778	7.983233	720.000061	6.319658	0.089022
**CS complex**	300	311	PSSGERYLSTAL	1280.6412	6.907252	0	0	0
**CS complex**	300	311	PSSGERYLSTAL	1280.6412	6.940645	0.033	2.430239	0.04671
**CS complex**	300	311	PSSGERYLSTAL	1280.6412	7.002673	1.667	4.78769	0.159835
**CS complex**	300	311	PSSGERYLSTAL	1280.6412	6.881904	16.667002	5.528757	0
**CS complex**	300	311	PSSGERYLSTAL	1280.6412	7.015262	60.000004	6.215368	0
**CS complex**	300	311	PSSGERYLSTAL	1280.6412	6.969331	720.000061	5.712536	0
**CysK**	300	311	PSSGERYLSTAL	1280.6412	6.902996	0	0	0
**CysK**	300	311	PSSGERYLSTAL	1280.6412	6.898749	0.033	2.501523	0.064933
**CysK**	300	311	PSSGERYLSTAL	1280.6412	6.967988	1.667	4.93434	0.063849
**CysK**	300	311	PSSGERYLSTAL	1280.6412	6.894449	16.667002	5.860375	0
**CysK**	300	311	PSSGERYLSTAL	1280.6412	6.976539	60.000004	5.901193	0
**CysK**	300	311	PSSGERYLSTAL	1280.6412	6.969856	720.000061	5.824434	0
**maxD control**	300	311	PSSGERYLSTAL	1280.6412	6.907252	0	0	0
**maxD control**	300	311	PSSGERYLSTAL	1280.6412	7.108418	720.000061	5.7098	0.089703
**CS complex**	304	311	ERYLSTAL	952.503	6.642999	0	0	0
**CS complex**	304	311	ERYLSTAL	952.503	6.680213	0.033	2.102952	0.06583
**CS complex**	304	311	ERYLSTAL	952.503	6.713329	1.667	3.717486	0.047241
**CS complex**	304	311	ERYLSTAL	952.503	6.630857	16.667002	4.279955	0.046925

**CS complex**	304	311	ERYLSTAL	952.503	6.716484	60.000004	4.283475	0.097629
**CS complex**	304	311	ERYLSTAL	952.503	6.717296	720.000061	4.196927	0.092899
**CysK**	304	311	ERYLSTAL	952.503	6.643903	0	0	0
**CysK**	304	311	ERYLSTAL	952.503	6.643431	0.033	2.160484	0.075748
**CysK**	304	311	ERYLSTAL	952.503	6.718313	1.667	3.882201	0.04637
**CysK**	304	311	ERYLSTAL	952.503	6.653904	16.667002	4.206774	0.027513
**CysK**	304	311	ERYLSTAL	952.503	6.729323	60.000004	4.269916	0.025794
**CysK**	304	311	ERYLSTAL	952.503	6.721028	720.000061	4.251796	0.039415
**maxD control**	304	311	ERYLSTAL	952.503	6.64251	0	0	0
**maxD control**	304	311	ERYLSTAL	952.503	6.870979	720.000061	4.110459	0.115262
**CS complex**	316	333	FTEKELQQTSLEHHHHHH	2275.068	4.066747	0	0	0
**CS complex**	316	333	FTEKELQQTSLEHHHHHH	2275.068	4.100889	0.033	6.155373	0.04087
**CS complex**	316	333	FTEKELQQTSLEHHHHHH	2275.068	4.115479	1.667	6.175293	0.052183
**CS complex**	316	333	FTEKELQQTSLEHHHHHH	2275.068	4.134032	16.667002	5.848736	0
**CS complex**	316	333	FTEKELQQTSLEHHHHHH	2275.068	4.128866	60.000004	6.349895	0
**CS complex**	316	333	FTEKELQQTSLEHHHHHH	2275.068	4.128721	720.000061	6.279996	0
**CysK**	316	333	FTEKELQQTSLEHHHHHH	2275.068	4.076198	0	0	0
**CysK**	316	333	FTEKELQQTSLEHHHHHH	2275.068	4.075824	0.033	6.244582	0.111098
**CysK**	316	333	FTEKELQQTSLEHHHHHH	2275.068	4.112149	1.667	6.240718	0.099895
**CysK**	316	333	FTEKELQQTSLEHHHHHH	2275.068	4.089364	16.667002	6.200315	0
**CysK**	316	333	FTEKELQQTSLEHHHHHH	2275.068	4.139862	60.000004	6.366231	0
**CysK**	316	333	FTEKELQQTSLEHHHHHH	2275.068	4.130645	720.000061	6.339667	0
**maxD control**	316	333	FTEKELQQTSLEHHHHHH	2275.068	4.066747	0	0	0
**maxD control**	316	333	FTEKELQQTSLEHHHHHH	2275.068	4.364241	720.000061	6.345028	0.112991
**CS complex**	324	333	TSLEHHHHHH	1271.577676	4.068463	0	0	0
**CS complex**	324	333	TSLEHHHHHH	1271.577676	4.092585	0.033	2.272641	0.049362
**CS complex**	324	333	TSLEHHHHHH	1271.577676	4.116645	1.667	2.253562	0.034796
**CS complex**	324	333	TSLEHHHHHH	1271.577676	4.129745	60.000004	2.362046	0.050478
**CS complex**	324	333	TSLEHHHHHH	1271.577676	4.131367	720.000061	2.414914	0.07465
**CysK**	324	333	TSLEHHHHHH	1271.577676	4.072215	0	0	0
**CysK**	324	333	TSLEHHHHHH	1271.577676	4.07578	0.033	2.286664	0.073126
**CysK**	324	333	TSLEHHHHHH	1271.577676	4.114022	1.667	2.261191	0.082777
**CysK**	324	333	TSLEHHHHHH	1271.577676	4.090357	16.667002	2.315191	0.06884
**CysK**	324	333	TSLEHHHHHH	1271.577676	4.140894	60.000004	2.349899	0.051952
**CysK**	324	333	TSLEHHHHHH	1271.577676	4.13077	720.000061	2.379717	0.067554
**maxD control**	324	333	TSLEHHHHHH	1271.577676	4.068463	0	0	0
**maxD control**	324	333	TSLEHHHHHH	1271.577676	4.366164	720.000061	2.173337	0.102244

Supplementary Table 4. HDX DATA TABLE CysE. DATA OUTPUT FOR KINETICS EXPERIMENTS (see Supplementary Dataset 4 for full-scale excel version).

Protein state	Start	End	Sequence	Peptide mass (Da)	Retention time (min)	HDX time (min)	Uptake (D)	Uptake SD (D)
CS complex	1	7	SGTSCEE	712.245411	3.376470	0	0	0
CS complex	1	7	SGTSCEE	712.245411	3.393990	0.033	2.80924	0.050818
CS complex	1	7	SGTSCEE	712.245411	3.414794	1.667	2.882292	0.027664
CS complex	1	7	SGTSCEE	712.245411	3.451391	60.000004	3.064193	0
CysE	1	7	SGTSCEE	712.245411	3.347365	0	0	0
CysE	1	7	SGTSCEE	712.245411	3.381499	0.033	2.716221	0.038712
CysE	1	7	SGTSCEE	712.245411	3.407563	1.667	2.776724	0.023909
CysE	1	7	SGTSCEE	712.245411	3.350226	16.667002	2.711647	0
CysE	1	7	SGTSCEE	712.245411	3.407516	60.000004	2.702874	0
maxD control	1	7	SGTSCEE	712.245411	3.376470	0	0	0
CS complex	1	8	SGTSCEEL	825.329475	6.098976	0	0	0
CS complex	1	8	SGTSCEEL	825.329475	6.131530	0.033	2.846161	0.030103
CS complex	1	8	SGTSCEEL	825.329475	6.157975	1.667	3.237142	0.048581
CS complex	1	8	SGTSCEEL	825.329475	6.081987	16.667002	3.194577	0
CS complex	1	8	SGTSCEEL	825.329475	6.170344	60.000004	3.158628	0
CysE	1	8	SGTSCEEL	825.329475	6.101342	0	0	0
CysE	1	8	SGTSCEEL	825.329475	6.128274	0.033	2.644462	0.037732
CysE	1	8	SGTSCEEL	825.329475	6.161405	1.667	3.129675	0.047812
CysE	1	8	SGTSCEEL	825.329475	6.097842	16.667002	3.073567	0
CysE	1	8	SGTSCEEL	825.329475	6.165736	60.000004	3.075225	0
maxD control	1	8	SGTSCEEL	825.329475	6.098976	0	0	0
maxD control	1	8	SGTSCEEL	825.329475	6.362781	720.000061	4.232226	0.046838
CS complex	8	17	LEIVWNNIKA	1199.671	7.976032	0	0	0
CS complex	8	17	LEIVWNNIKA	1199.671	8.023772	0.033	0.454469	0.016716
CS complex	8	17	LEIVWNNIKA	1199.671	8.066896	1.667	2.987115	0.094932
CS complex	8	17	LEIVWNNIKA	1199.671	7.961702	16.667002	4.421138	0
CS complex	8	17	LEIVWNNIKA	1199.671	8.065548	60.000004	4.956872	0
CS complex	8	17	LEIVWNNIKA	1199.671	8.062929	720.000061	5.218404	0
CysE	8	17	LEIVWNNIKA	1199.671	7.980533	0	0	0
CysE	8	17	LEIVWNNIKA	1199.671	8.018882	0.033	0.398629	0.031116
CysE	8	17	LEIVWNNIKA	1199.671	8.065139	1.667	3.544135	0.026936
CysE	8	17	LEIVWNNIKA	1199.671	7.972692	16.667002	4.658622	0
CysE	8	17	LEIVWNNIKA	1199.671	8.067355	60.000004	5.164823	0
CysE	8	17	LEIVWNNIKA	1199.671	8.065930	720.000061	5.355799	0
maxD control	8	17	LEIVWNNIKA	1199.671	7.976032	0	0	0

**maxD control**	8	17	LEIVWNNIKA	1199.671	8.192841	720.000061	5.399732	0.201434
**CS complex**	8	19	LEIVWNNIKAEA	1399.7507	7.914838	0	0	0
**CS complex**	8	19	LEIVWNNIKAEA	1399.7507	7.956691	0.033	0.524959	0.010451
**CS complex**	8	19	LEIVWNNIKAEA	1399.7507	7.993749	1.667	3.158994	0.058532
**CS complex**	8	19	LEIVWNNIKAEA	1399.7507	7.902947	16.667002	4.938743	0
**CS complex**	8	19	LEIVWNNIKAEA	1399.7507	7.991818	60.000004	6.130533	0
**CS complex**	8	19	LEIVWNNIKAEA	1399.7507	7.973561	720.000061	6.584891	0
**CysE**	8	19	LEIVWNNIKAEA	1399.7507	7.923277	0	0	0
**CysE**	8	19	LEIVWNNIKAEA	1399.7507	7.957481	0.033	0.42699	0.017416
**CysE**	8	19	LEIVWNNIKAEA	1399.7507	7.997482	1.667	3.845757	0.053436
**CysE**	8	19	LEIVWNNIKAEA	1399.7507	7.906903	16.667002	5.644739	0
**CysE**	8	19	LEIVWNNIKAEA	1399.7507	7.993382	60.000004	6.480128	0
**CysE**	8	19	LEIVWNNIKAEA	1399.7507	7.991583	720.000061	6.972145	0
**maxD control**	8	19	LEIVWNNIKAEA	1399.7507	7.914838	0	0	0
**maxD control**	8	19	LEIVWNNIKAEA	1399.7507	7.990525	720.000061	9.114032	0.135975
**CS complex**	8	21	LEIVWNNIKAEART	1656.8995	7.214670	0	0	0
**CS complex**	8	21	LEIVWNNIKAEART	1656.8995	7.262631	0.033	1.125959	0.043825
**CS complex**	8	21	LEIVWNNIKAEART	1656.8995	7.286031	1.667	4.658335	0.082814
**CS complex**	8	21	LEIVWNNIKAEART	1656.8995	7.188894	16.667002	6.533366	0
**CS complex**	8	21	LEIVWNNIKAEART	1656.8995	7.281793	60.000004	7.777613	2.27E-13
**CS complex**	8	21	LEIVWNNIKAEART	1656.8995	7.281450	720.000061	9.928746	0
**CysE**	8	21	LEIVWNNIKAEART	1656.8995	7.215877	0	0	0
**CysE**	8	21	LEIVWNNIKAEART	1656.8995	7.249559	0.033	0.86838	0.042713
**CysE**	8	21	LEIVWNNIKAEART	1656.8995	7.286420	1.667	5.429423	0.014439
**CysE**	8	21	LEIVWNNIKAEART	1656.8995	7.198352	16.667002	7.217971	0
**CysE**	8	21	LEIVWNNIKAEART	1656.8995	7.284794	60.000004	8.193694	0
**CysE**	8	21	LEIVWNNIKAEART	1656.8995	7.281937	720.000061	10.300184	0
**maxD control**	8	21	LEIVWNNIKAEART	1656.8995	7.214646	0	0	0
**maxD control**	8	21	LEIVWNNIKAEART	1656.8995	7.549959	720.000061	7.814488	0.469745
**CS complex**	8	23	LEIVWNNIKAEARTLA	1841.0208	8.201705	0	0	0
**CS complex**	8	23	LEIVWNNIKAEARTLA	1841.0208	8.248284	0.033	1.196263	0.067083
**CS complex**	8	23	LEIVWNNIKAEARTLA	1841.0208	8.266167	1.667	5.461065	0.123779
**CS complex**	8	23	LEIVWNNIKAEARTLA	1841.0208	8.151255	16.667002	7.798298	0.04737
**CS complex**	8	23	LEIVWNNIKAEARTLA	1841.0208	8.245555	60.000004	9.192309	0.07245
**CS complex**	8	23	LEIVWNNIKAEARTLA	1841.0208	8.274560	720.000061	10.996341	0.042102
**CysE**	8	23	LEIVWNNIKAEARTLA	1841.0208	8.204641	0	0	0
**CysE**	8	23	LEIVWNNIKAEARTLA	1841.0208	8.238622	0.033	1.02175	0.104872
**CysE**	8	23	LEIVWNNIKAEARTLA	1841.0208	8.263047	1.667	6.43691	0.118912
**CysE**	8	23	LEIVWNNIKAEARTLA	1841.0208	8.156779	16.667002	8.580021	0.047372
**CysE**	8	23	LEIVWNNIKAEARTLA	1841.0208	8.247028	60.000004	9.788471	0.142076
**CysE**	8	23	LEIVWNNIKAEARTLA	1841.0208	8.275059	720.000061	11.276521	0.047301

**maxD control**	8	23	LEIVWNNIKAEARTLA	1841.0208	8.201690	0	0	0
**maxD control**	8	23	LEIVWNNIKAEARTLA	1841.0208	8.415424	720.000061	12.766851	0.113729
**CS complex**	9	17	EIVWNNIKA	1086.5869	7.263283	0	0	0
**CS complex**	9	17	EIVWNNIKA	1086.5869	7.307394	0.033	0.334504	0.050361
**CS complex**	9	17	EIVWNNIKA	1086.5869	7.338336	1.667	2.556444	0.007235
**CS complex**	9	17	EIVWNNIKA	1086.5869	7.255678	16.667002	3.445048	0
**CS complex**	9	17	EIVWNNIKA	1086.5869	7.341042	60.000004	3.957247	0
**CS complex**	9	17	EIVWNNIKA	1086.5869	7.344824	720.000061	4.482598	0
**CysE**	9	17	EIVWNNIKA	1086.5869	7.264539	0	0	0
**CysE**	9	17	EIVWNNIKA	1086.5869	7.300594	0.033	0.359873	0.029161
**CysE**	9	17	EIVWNNIKA	1086.5869	7.339931	1.667	2.796198	0.054692
**CysE**	9	17	EIVWNNIKA	1086.5869	7.254226	16.667002	3.76992	0
**CysE**	9	17	EIVWNNIKA	1086.5869	7.344290	60.000004	4.239749	0
**CysE**	9	17	EIVWNNIKA	1086.5869	7.342090	720.000061	4.808624	0
**maxD control**	9	17	EIVWNNIKA	1086.5869	7.263283	0	0	0
**maxD control**	9	17	EIVWNNIKA	1086.5869	7.450839	720.000061	4.87041	0.113379
**CS complex**	9	21	EIVWNNIKAEART	1543.8154	6.531430	0	0	0
**CS complex**	9	21	EIVWNNIKAEART	1543.8154	6.569177	0.033	0.957245	0.059519
**CS complex**	9	21	EIVWNNIKAEART	1543.8154	6.593750	1.667	4.044741	0.062545
**CS complex**	9	21	EIVWNNIKAEART	1543.8154	6.501819	16.667002	5.698192	0.07965
**CS complex**	9	21	EIVWNNIKAEART	1543.8154	6.588335	60.000004	6.820469	0.057948
**CS complex**	9	21	EIVWNNIKAEART	1543.8154	6.590795	720.000061	7.698902	0.166135
**CysE**	9	21	EIVWNNIKAEART	1543.8154	6.545852	0	0	0
**CysE**	9	21	EIVWNNIKAEART	1543.8154	6.567814	0.033	0.839829	0.088883
**CysE**	9	21	EIVWNNIKAEART	1543.8154	6.595237	1.667	4.435627	0.101655
**CysE**	9	21	EIVWNNIKAEART	1543.8154	6.514823	16.667002	6.149097	0.097176
**CysE**	9	21	EIVWNNIKAEART	1543.8154	6.589820	60.000004	7.231789	0.069285
**CysE**	9	21	EIVWNNIKAEART	1543.8154	6.587176	720.000061	8.113578	0.072617
**maxD control**	9	21	EIVWNNIKAEART	1543.8154	6.531430	0	0	0
**maxD control**	9	21	EIVWNNIKAEART	1543.8154	6.733624	720.000061	8.097501	0.137586
**CS complex**	10	21	IVWNNIKAEART	1414.7728	6.091010	0	0	0
**CS complex**	10	21	IVWNNIKAEART	1414.7728	6.119370	0.033	0.955479	0.051415
**CS complex**	10	21	IVWNNIKAEART	1414.7728	6.148279	1.667	3.250749	0.038447
**CS complex**	10	21	IVWNNIKAEART	1414.7728	6.066948	16.667002	4.547476	2.27E-13
**CS complex**	10	21	IVWNNIKAEART	1414.7728	6.149070	60.000004	5.683653	0
**CS complex**	10	21	IVWNNIKAEART	1414.7728	6.146509	720.000061	6.85616	0
**CysE**	10	21	IVWNNIKAEART	1414.7728	6.091511	0	0	0
**CysE**	10	21	IVWNNIKAEART	1414.7728	6.118938	0.033	0.883318	0.075385
**CysE**	10	21	IVWNNIKAEART	1414.7728	6.153158	1.667	3.564721	0.029918
**CysE**	10	21	IVWNNIKAEART	1414.7728	6.079552	16.667002	4.931012	0
**CysE**	10	21	IVWNNIKAEART	1414.7728	6.149500	60.000004	6.149146	0

**CysE**	10	21	IVWNNIKAEART	1414.7728	6.144140	720.000061	7.149502	0
**maxD control**	10	21	IVWNNIKAEART	1414.7728	6.091010	0	0	0
**maxD control**	10	21	IVWNNIKAEART	1414.7728	6.302297	720.000061	7.148147	0.094171
**CS complex**	10	22	IVWNNIKAEARTL	1527.857	7.226615	0	0	0
**CS complex**	10	22	IVWNNIKAEARTL	1527.857	7.264314	0.033	1.002555	0.045018
**CS complex**	10	22	IVWNNIKAEARTL	1527.857	7.292831	1.667	3.78139	0.049682
**CS complex**	10	22	IVWNNIKAEARTL	1527.857	7.193853	16.667002	5.417836	0
**CS complex**	10	22	IVWNNIKAEARTL	1527.857	7.283151	60.000004	6.559211	2.27E-13
**CS complex**	10	22	IVWNNIKAEARTL	1527.857	7.276295	720.000061	7.689146	0
**CysE**	10	22	IVWNNIKAEARTL	1527.857	7.230346	0	0	0
**CysE**	10	22	IVWNNIKAEARTL	1527.857	7.262021	0.033	0.822233	0.069044
**CysE**	10	22	IVWNNIKAEARTL	1527.857	7.297292	1.667	4.044044	0.057763
**CysE**	10	22	IVWNNIKAEARTL	1527.857	7.202278	16.667002	5.660972	0
**CysE**	10	22	IVWNNIKAEARTL	1527.857	7.286359	60.000004	6.887023	0
**CysE**	10	22	IVWNNIKAEARTL	1527.857	7.276858	720.000061	7.773308	0
**maxD control**	10	22	IVWNNIKAEARTL	1527.857	7.226615	0	0	0
**maxD control**	10	22	IVWNNIKAEARTL	1527.857	7.426837	720.000061	7.914358	0.184702
**CS complex**	10	23	IVWNNIKAEARTLA	1598.894	7.245727	0	0	0
**CS complex**	10	23	IVWNNIKAEARTLA	1598.894	7.283219	0.033	1.150688	0.107009
**CS complex**	10	23	IVWNNIKAEARTLA	1598.894	7.305532	1.667	4.388036	0.038537
**CS complex**	10	23	IVWNNIKAEARTLA	1598.894	7.211648	16.667002	5.916462	0.047356
**CS complex**	10	23	IVWNNIKAEARTLA	1598.894	7.291844	60.000004	7.396156	0.023958
**CS complex**	10	23	IVWNNIKAEARTLA	1598.894	7.285402	720.000061	8.441877	0.029088
**CysE**	10	23	IVWNNIKAEARTLA	1598.894	7.250489	0	0	0
**CysE**	10	23	IVWNNIKAEARTLA	1598.894	7.279013	0.033	0.973006	0.112074
**CysE**	10	23	IVWNNIKAEARTLA	1598.894	7.307822	1.667	4.780373	0.086432
**CysE**	10	23	IVWNNIKAEARTLA	1598.894	7.212474	16.667002	6.519343	0.045575
**CysE**	10	23	IVWNNIKAEARTLA	1598.894	7.294653	60.000004	7.784925	0.047254
**CysE**	10	23	IVWNNIKAEARTLA	1598.894	7.287148	720.000061	8.657001	0.051371
**maxD control**	10	23	IVWNNIKAEARTLA	1598.894	7.245727	0	0	0
**maxD control**	10	23	IVWNNIKAEARTLA	1598.894	7.435048	720.000061	8.646509	0.119761
**CS complex**	10	26	IVWNNIKAEARTLADCE	1945.973	7.956148	0	0	0
**CS complex**	10	26	IVWNNIKAEARTLADCE	1945.973	7.999075	0.033	1.701451	0.068703
**CS complex**	10	26	IVWNNIKAEARTLADCE	1945.973	8.015800	1.667	5.920791	0.102792
**CS complex**	10	26	IVWNNIKAEARTLADCE	1945.973	7.909557	16.667002	7.820434	2.27E-13
**CS complex**	10	26	IVWNNIKAEARTLADCE	1945.973	8.001083	60.000004	9.163802	0
**CS complex**	10	26	IVWNNIKAEARTLADCE	1945.973	7.988417	720.000061	10.159535	0
**CysE**	10	26	IVWNNIKAEARTLADCE	1945.973	7.960688	0	0	0
**CysE**	10	26	IVWNNIKAEARTLADCE	1945.973	7.994771	0.033	1.487766	0.111012
**CysE**	10	26	IVWNNIKAEARTLADCE	1945.973	8.013794	1.667	6.594447	0.029547
**CysE**	10	26	IVWNNIKAEARTLADCE	1945.973	7.912086	16.667002	8.329087	3.22E-13

**CysE**	10	26	IVWNNIKAEARTLADCE	1945.973	7.998252	60.000004	9.532809	2.27E-13
**CysE**	10	26	IVWNNIKAEARTLADCE	1945.973	7.993078	720.000061	10.409335	2.27E-13
**maxD control**	10	26	IVWNNIKAEARTLADCE	1945.973	7.956148	0	0	0
**maxD control**	10	26	IVWNNIKAEARTLADCE	1945.973	8.119884	720.000061	10.139396	0.071473
**CS complex**	12	21	WNNIKAEART	1202.6204	6.534048	0	0	0
**CS complex**	12	21	WNNIKAEART	1202.6204	6.317258	0.033	1.043547	0.171979
**CS complex**	12	21	WNNIKAEART	1202.6204	6.139807	1.667	3.058664	0.039321
**CS complex**	12	21	WNNIKAEART	1202.6204	6.502333	16.667002	4.948869	0.038627
**CS complex**	12	21	WNNIKAEART	1202.6204	6.141635	60.000004	5.429362	0.038627
**CS complex**	12	21	WNNIKAEART	1202.6204	6.138089	720.000061	6.311171	0.038627
**CysE**	12	21	WNNIKAEART	1202.6204	6.090951	0	0	0
**CysE**	12	21	WNNIKAEART	1202.6204	6.115664	0.033	0.906476	0.082481
**CysE**	12	21	WNNIKAEART	1202.6204	6.151540	1.667	3.260131	0.049749
**CysE**	12	21	WNNIKAEART	1202.6204	6.079000	16.667002	4.720596	0
**CysE**	12	21	WNNIKAEART	1202.6204	6.147583	60.000004	5.738971	0
**CysE**	12	21	WNNIKAEART	1202.6204	6.142732	720.000061	6.570162	0
**maxD control**	12	21	WNNIKAEART	1202.6204	6.534235	0	0	0
**maxD control**	12	21	WNNIKAEART	1202.6204	6.448050	720.000061	6.889009	0.077785
**CS complex**	18	29	EARTLADCEPML	1348.6166	7.793537	0	0	0
**CS complex**	18	29	EARTLADCEPML	1348.6166	7.837936	0.033	2.046381	0.09465
**CS complex**	18	29	EARTLADCEPML	1348.6166	7.875876	1.667	4.952107	0.047129
**CS complex**	18	29	EARTLADCEPML	1348.6166	7.780304	16.667002	5.840505	0
**CS complex**	18	29	EARTLADCEPML	1348.6166	7.876690	60.000004	6.112597	0
**CS complex**	18	29	EARTLADCEPML	1348.6166	7.876514	720.000061	6.065106	0
**CysE**	18	29	EARTLADCEPML	1348.6166	7.799802	0	0	0
**CysE**	18	29	EARTLADCEPML	1348.6166	7.834648	0.033	1.754306	0.12298
**CysE**	18	29	EARTLADCEPML	1348.6166	7.875888	1.667	5.239777	0.031442
**CysE**	18	29	EARTLADCEPML	1348.6166	7.786168	16.667002	6.003201	0
**CysE**	18	29	EARTLADCEPML	1348.6166	7.873120	60.000004	6.141256	0
**CysE**	18	29	EARTLADCEPML	1348.6166	7.875021	720.000061	6.194539	0
**maxD control**	18	29	EARTLADCEPML	1348.6166	7.793537	0	0	0
**maxD control**	18	29	EARTLADCEPML	1348.6166	7.477215	720.000061	5.44841	0.157352
**CS complex**	20	29	RTLADCEPML	1148.5369	7.601039	0	0	0
**CS complex**	20	29	RTLADCEPML	1148.5369	7.642837	0.033	1.422029	0.057344
**CS complex**	20	29	RTLADCEPML	1148.5369	7.676751	1.667	3.804711	0.035001
**CS complex**	20	29	RTLADCEPML	1148.5369	7.586478	16.667002	4.603107	0
**CS complex**	20	29	RTLADCEPML	1148.5369	7.678655	60.000004	4.901282	0
**CS complex**	20	29	RTLADCEPML	1148.5369	7.679816	720.000061	4.887117	0
**CysE**	20	29	RTLADCEPML	1148.5369	7.602534	0	0	0
**CysE**	20	29	RTLADCEPML	1148.5369	7.632186	0.033	1.187144	0.087768
**CysE**	20	29	RTLADCEPML	1148.5369	7.675018	1.667	4.009503	0.068347

**CysE**	20	29	RTLADCEPML	1148.5369	7.588795	16.667002	4.634818	0
**CysE**	20	29	RTLADCEPML	1148.5369	7.672035	60.000004	4.83955	0
**CysE**	20	29	RTLADCEPML	1148.5369	7.671485	720.000061	4.901508	0
**maxD control**	20	29	RTLADCEPML	1148.5369	7.601039	0	0	0
**maxD control**	20	29	RTLADCEPML	1148.5369	7.864760	720.000061	4.916076	0.026965
**CS complex**	22	26	LADCE	550.2106	4.502817	0	0	0
**CS complex**	22	26	LADCE	550.2106	4.533865	0.033	0.813803	0.026797
**CS complex**	22	26	LADCE	550.2106	4.559845	1.667	2.002818	0.016354
**CS complex**	22	26	LADCE	550.2106	4.556912	16.667002	2.042663	0
**CS complex**	22	26	LADCE	550.2106	4.573484	60.000004	2.12429	0
**CS complex**	22	26	LADCE	550.2106	4.573754	720.000061	2.101705	0
**CysE**	22	26	LADCE	550.2106	4.496787	0	0	0
**CysE**	22	26	LADCE	550.2106	4.526904	0.033	0.712643	0.040804
**CysE**	22	26	LADCE	550.2106	4.561649	1.667	2.068911	0.021439
**CysE**	22	26	LADCE	550.2106	4.499382	16.667002	2.062152	0
**CysE**	22	26	LADCE	550.2106	4.561462	60.000004	2.127286	0
**CysE**	22	26	LADCE	550.2106	4.572051	720.000061	2.1406	0
**maxD control**	22	26	LADCE	550.2106	4.502817	0	0	0
**maxD control**	22	26	LADCE	550.2106	4.599584	720.000061	3.580622	0.024813
**CS complex**	30	37	ASFYHATL	909.4393	6.858321	0	0	0
**CS complex**	30	37	ASFYHATL	909.4393	6.900162	0.033	0.598981	0.07128
**CS complex**	30	37	ASFYHATL	909.4393	6.934804	1.667	0.817156	0.075543
**CS complex**	30	37	ASFYHATL	909.4393	6.852010	16.667002	1.16989	0.077633
**CS complex**	30	37	ASFYHATL	909.4393	6.939672	60.000004	1.78457	0.159519
**CS complex**	30	37	ASFYHATL	909.4393	6.934943	720.000061	3.294171	0.066382
**CysE**	30	37	ASFYHATL	909.4393	6.861961	0	0	0
**CysE**	30	37	ASFYHATL	909.4393	6.897383	0.033	0.490337	0.067674
**CysE**	30	37	ASFYHATL	909.4393	6.941795	1.667	0.83254	0.063302
**CysE**	30	37	ASFYHATL	909.4393	6.862514	16.667002	1.210689	0.067687
**CysE**	30	37	ASFYHATL	909.4393	6.943984	60.000004	1.930115	0.15735
**CysE**	30	37	ASFYHATL	909.4393	6.936732	720.000061	3.35221	0.066793
**maxD control**	30	37	ASFYHATL	909.4393	6.858190	0	0	0
**maxD control**	30	37	ASFYHATL	909.4393	7.097738	720.000061	3.528496	0.084953
**CS complex**	30	50	ASFYHATLLKHENLGSALSYM	2353.164529	7.758383	0	0	0
**CS complex**	30	50	ASFYHATLLKHENLGSALSYM	2353.164529	7.806159	0.033	0.995295	0.049882
**CS complex**	30	50	ASFYHATLLKHENLGSALSYM	2353.164529	7.846250	1.667	2.931868	0.053238
**CS complex**	30	50	ASFYHATLLKHENLGSALSYM	2353.164529	7.749499	16.667002	3.099442	0
**CS complex**	30	50	ASFYHATLLKHENLGSALSYM	2353.164529	7.852305	60.000004	3.557128	0
**CS complex**	30	50	ASFYHATLLKHENLGSALSYM	2353.164529	7.848268	720.000061	4.589277	0
**CysE**	30	50	ASFYHATLLKHENLGSALSYM	2353.164529	7.760023	0	0	0
**CysE**	30	50	ASFYHATLLKHENLGSALSYM	2353.164529	7.797709	0.033	0.94587	0.0728

**CysE**	30	50	ASFYHATLLKHENLGSALSYM	2353.164529	7.846209	1.667	3.046957	0.015963
**CysE**	30	50	ASFYHATLLKHENLGSALSYM	2353.164529	7.753189	16.667002	3.183757	0
**CysE**	30	50	ASFYHATLLKHENLGSALSYM	2353.164529	7.849057	60.000004	3.569954	0
**CysE**	30	50	ASFYHATLLKHENLGSALSYM	2353.164529	7.844261	720.000061	4.761522	0
**maxD control**	30	50	ASFYHATLLKHENLGSALSYM	2353.164529	7.758383	0	0	0
**maxD control**	30	50	ASFYHATLLKHENLGSALSYM	2353.164529	8.015172	720.000061	9.322023	0
**CS complex**	33	47	YHATLLKHENLGSAL	1666.891131	6.136073	0	0	0
**CS complex**	33	47	YHATLLKHENLGSAL	1666.891131	6.177141	0.033	0.961775	0.044709
**CS complex**	33	47	YHATLLKHENLGSAL	1666.891131	6.212236	1.667	2.648347	0.053143
**CS complex**	33	47	YHATLLKHENLGSAL	1666.891131	6.114441	16.667002	3.103177	0.036966
**CS complex**	33	47	YHATLLKHENLGSAL	1666.891131	6.216964	60.000004	3.167437	0.053259
**CS complex**	33	47	YHATLLKHENLGSAL	1666.891131	6.218181	720.000061	3.878106	0.048501
**CysE**	33	47	YHATLLKHENLGSAL	1666.891131	6.145610	0	0	0
**CysE**	33	47	YHATLLKHENLGSAL	1666.891131	6.173254	0.033	0.793976	0.08177
**CysE**	33	47	YHATLLKHENLGSAL	1666.891131	6.214883	1.667	2.649432	0.04866
**CysE**	33	47	YHATLLKHENLGSAL	1666.891131	6.138496	16.667002	2.881995	0.042832
**CysE**	33	47	YHATLLKHENLGSAL	1666.891131	6.216448	60.000004	3.052332	0.049321
**CysE**	33	47	YHATLLKHENLGSAL	1666.891131	6.213981	720.000061	3.998181	0.041509
**maxD control**	33	47	YHATLLKHENLGSAL	1666.891131	6.136085	0	0	0
**maxD control**	33	47	YHATLLKHENLGSAL	1666.891131	6.407122	720.000061	6.012011	0.117958
**CS complex**	38	46	LKHENLGSA	968.5087	3.854591	0	0	0
**CS complex**	38	46	LKHENLGSA	968.5087	3.889908	0.033	1.203364	0.023441
**CS complex**	38	46	LKHENLGSA	968.5087	3.911682	1.667	2.582129	0.016614
**CS complex**	38	46	LKHENLGSA	968.5087	3.932191	60.000004	2.528572	0
**CS complex**	38	46	LKHENLGSA	968.5087	3.931914	720.000061	2.561573	0
**CysE**	38	46	LKHENLGSA	968.5087	3.849287	0	0	0
**CysE**	38	46	LKHENLGSA	968.5087	3.881287	0.033	0.976425	0.081727
**CysE**	38	46	LKHENLGSA	968.5087	3.913005	1.667	2.486703	0.033834
**CysE**	38	46	LKHENLGSA	968.5087	3.855687	16.667002	2.445543	0
**CysE**	38	46	LKHENLGSA	968.5087	3.910316	60.000004	2.474402	0
**CysE**	38	46	LKHENLGSA	968.5087	3.932813	720.000061	2.617516	0
**maxD control**	38	46	LKHENLGSA	968.5087	3.854425	0	0	0
**maxD control**	38	46	LKHENLGSA	968.5087	4.117317	720.000061	3.003598	0.034327
**CS complex**	38	47	LKHENLGSAL	1081.5927	5.574953	0	0	0
**CS complex**	38	47	LKHENLGSAL	1081.5927	5.609022	0.033	0.926375	0.027109
**CS complex**	38	47	LKHENLGSAL	1081.5927	5.632919	1.667	2.379575	0.019264
**CS complex**	38	47	LKHENLGSAL	1081.5927	5.571939	16.667002	2.412906	0
**CS complex**	38	47	LKHENLGSAL	1081.5927	5.639020	60.000004	2.433014	0
**CS complex**	38	47	LKHENLGSAL	1081.5927	5.641152	720.000061	2.438698	0
**CysE**	38	47	LKHENLGSAL	1081.5927	5.580190	0	0	0
**CysE**	38	47	LKHENLGSAL	1081.5927	5.601262	0.033	0.812931	0.044505

**CysE**	38	47	LKHENLGSAL	1081.5927	5.642030	1.667	2.442254	0.011236
**CysE**	38	47	LKHENLGSAL	1081.5927	5.574862	16.667002	2.424833	0
**CysE**	38	47	LKHENLGSAL	1081.5927	5.643159	60.000004	2.486723	0
**CysE**	38	47	LKHENLGSAL	1081.5927	5.645223	720.000061	2.522351	0
**maxD control**	38	47	LKHENLGSAL	1081.5927	5.574931	0	0	0
**maxD control**	38	47	LKHENLGSAL	1081.5927	5.850317	720.000061	3.789574	0.073086
**CS complex**	38	48	LKHENLGSALS	1168.6248	5.166171	0	0	0
**CS complex**	38	48	LKHENLGSALS	1168.6248	5.200277	0.033	1.007818	0.025372
**CS complex**	38	48	LKHENLGSALS	1168.6248	5.220852	1.667	2.310292	0.014221
**CS complex**	38	48	LKHENLGSALS	1168.6248	5.149956	16.667002	2.337566	0
**CS complex**	38	48	LKHENLGSALS	1168.6248	5.228132	60.000004	2.385302	0
**CS complex**	38	48	LKHENLGSALS	1168.6248	5.228013	720.000061	2.484764	0
**CysE**	38	48	LKHENLGSALS	1168.6248	5.166581	0	0	0
**CysE**	38	48	LKHENLGSALS	1168.6248	5.197114	0.033	0.86469	0.048268
**CysE**	38	48	LKHENLGSALS	1168.6248	5.226645	1.667	2.314312	0.018731
**CysE**	38	48	LKHENLGSALS	1168.6248	5.163064	16.667002	2.333887	0
**CysE**	38	48	LKHENLGSALS	1168.6248	5.230265	60.000004	2.343632	0
**CysE**	38	48	LKHENLGSALS	1168.6248	5.228314	720.000061	2.556435	0
**maxD control**	38	48	LKHENLGSALS	1168.6248	5.166167	0	0	0
**maxD control**	38	48	LKHENLGSALS	1168.6248	5.421011	720.000061	4.335923	0.032511
**CS complex**	38	49	LKHENLGSALSY	1331.688	6.190700	0	0	0
**CS complex**	38	49	LKHENLGSALSY	1331.688	6.230524	0.033	0.938432	0.019526
**CS complex**	38	49	LKHENLGSALSY	1331.688	6.261062	1.667	2.199401	0.025017
**CS complex**	38	49	LKHENLGSALSY	1331.688	6.201571	16.667002	2.221318	2.27E-13
**CS complex**	38	49	LKHENLGSALSY	1331.688	6.264664	60.000004	2.374475	0
**CS complex**	38	49	LKHENLGSALSY	1331.688	6.266358	720.000061	2.387309	0
**CysE**	38	49	LKHENLGSALSY	1331.688	6.197726	0	0	0
**CysE**	38	49	LKHENLGSALSY	1331.688	6.228016	0.033	0.883188	0.060967
**CysE**	38	49	LKHENLGSALSY	1331.688	6.265644	1.667	2.339608	0.014081
**CysE**	38	49	LKHENLGSALSY	1331.688	6.192739	16.667002	2.429692	0
**CysE**	38	49	LKHENLGSALSY	1331.688	6.268755	60.000004	2.507822	0
**CysE**	38	49	LKHENLGSALSY	1331.688	6.265133	720.000061	2.554627	0
**maxD control**	38	49	LKHENLGSALSY	1331.688	6.190815	0	0	0
**maxD control**	38	49	LKHENLGSALSY	1331.688	6.449075	720.000061	5.084573	0.140966
**CS complex**	50	60	MLANKLSSPIM	1204.636	7.070284	0	0	0
**CS complex**	50	60	MLANKLSSPIM	1204.636	7.101219	0.033	1.042792	0.031453
**CS complex**	50	60	MLANKLSSPIM	1204.636	7.137578	1.667	2.403121	0.018873
**CS complex**	50	60	MLANKLSSPIM	1204.636	7.055493	16.667002	3.470218	0
**CS complex**	50	60	MLANKLSSPIM	1204.636	7.133482	60.000004	4.038393	0
**CS complex**	50	60	MLANKLSSPIM	1204.636	7.133418	720.000061	4.466547	2.27E-13
**CysE**	50	60	MLANKLSSPIM	1204.636	7.073021	0	0	0

**CysE**	50	60	MLANKLSSPIM	1204.636	7.101439	0.033	0.905665	0.06071
**CysE**	50	60	MLANKLSSPIM	1204.636	7.141307	1.667	2.503559	0.066657
**CysE**	50	60	MLANKLSSPIM	1204.636	7.063838	16.667002	3.809613	0
**CysE**	50	60	MLANKLSSPIM	1204.636	7.140102	60.000004	4.164354	0
**CysE**	50	60	MLANKLSSPIM	1204.636	7.137144	720.000061	4.779695	0
**maxD control**	50	60	MLANKLSSPIM	1204.636	7.070248	0	0	0
**maxD control**	50	60	MLANKLSSPIM	1204.636	7.278554	720.000061	5.130399	0.083384
**CS complex**	51	60	LANKLSSPIM	1073.5955	6.548738	0	0	0
**CS complex**	51	60	LANKLSSPIM	1073.5955	6.580547	0.033	1.131857	0.030818
**CS complex**	51	60	LANKLSSPIM	1073.5955	6.611848	1.667	2.452862	0.050297
**CS complex**	51	60	LANKLSSPIM	1073.5955	6.530045	16.667002	3.578634	0
**CS complex**	51	60	LANKLSSPIM	1073.5955	6.612083	60.000004	3.896553	0
**CS complex**	51	60	LANKLSSPIM	1073.5955	6.615470	720.000061	4.272087	0
**CysE**	51	60	LANKLSSPIM	1073.5955	6.556376	0	0	0
**CysE**	51	60	LANKLSSPIM	1073.5955	6.576049	0.033	0.987798	0.057185
**CysE**	51	60	LANKLSSPIM	1073.5955	6.618575	1.667	2.568404	0.008017
**CysE**	51	60	LANKLSSPIM	1073.5955	6.551186	16.667002	3.731373	0
**CysE**	51	60	LANKLSSPIM	1073.5955	6.616155	60.000004	4.058618	0
**CysE**	51	60	LANKLSSPIM	1073.5955	6.614356	720.000061	4.676421	0
**maxD control**	51	60	LANKLSSPIM	1073.5955	6.548738	0	0	0
**maxD control**	51	60	LANKLSSPIM	1073.5955	6.775304	720.000061	4.52065	0.061154
**CS complex**	51	64	LANKLSSPIMPAIA	1425.8066	7.338434	0	0	0
**CS complex**	51	64	LANKLSSPIMPAIA	1425.8066	7.373120	0.033	2.011022	0.037915
**CS complex**	51	64	LANKLSSPIMPAIA	1425.8066	7.414142	1.667	3.875053	0.051898
**CS complex**	51	64	LANKLSSPIMPAIA	1425.8066	7.317515	16.667002	5.360629	0
**CS complex**	51	64	LANKLSSPIMPAIA	1425.8066	7.410669	60.000004	5.788651	0
**CS complex**	51	64	LANKLSSPIMPAIA	1425.8066	7.412428	720.000061	6.249497	0
**CysE**	51	64	LANKLSSPIMPAIA	1425.8066	7.338168	0	0	0
**CysE**	51	64	LANKLSSPIMPAIA	1425.8066	7.370049	0.033	1.858368	0.067849
**CysE**	51	64	LANKLSSPIMPAIA	1425.8066	7.411930	1.667	4.053216	0.032662
**CysE**	51	64	LANKLSSPIMPAIA	1425.8066	7.327270	16.667002	5.556385	0
**CysE**	51	64	LANKLSSPIMPAIA	1425.8066	7.408880	60.000004	5.913781	0
**CysE**	51	64	LANKLSSPIMPAIA	1425.8066	7.410672	720.000061	6.590783	0
**maxD control**	51	64	LANKLSSPIMPAIA	1425.8066	7.338419	0	0	0
**maxD control**	51	64	LANKLSSPIMPAIA	1425.8066	7.545230	720.000061	6.144462	0.050882
**CS complex**	53	60	NKLSSPIM	889.4743	6.144658	0	0	0
**CS complex**	53	60	NKLSSPIM	889.4743	6.182407	0.033	1.055169	0.021857
**CS complex**	53	60	NKLSSPIM	889.4743	6.213077	1.667	2.020519	0.025214
**CS complex**	53	60	NKLSSPIM	889.4743	6.128592	16.667002	2.721997	0
**CS complex**	53	60	NKLSSPIM	889.4743	6.214904	60.000004	3.023912	0
**CS complex**	53	60	NKLSSPIM	889.4743	6.215947	720.000061	3.363517	0

**CysE**	53	60	NKLSSPIM	889.4743	6.152368	0	0	0
**CysE**	53	60	NKLSSPIM	889.4743	6.176689	0.033	0.901368	0.05336
**CysE**	53	60	NKLSSPIM	889.4743	6.218095	1.667	2.075773	0.025742
**CysE**	53	60	NKLSSPIM	889.4743	6.147508	16.667002	2.837975	0
**CysE**	53	60	NKLSSPIM	889.4743	6.218175	60.000004	3.043657	0
**CysE**	53	60	NKLSSPIM	889.4743	6.216455	720.000061	3.536014	0
**maxD control**	53	60	NKLSSPIM	889.4743	6.144658	0	0	0
**maxD control**	53	60	NKLSSPIM	889.4743	6.385057	720.000061	3.490673	0.030693
**CS complex**	65	72	IREVVEEA	944.4977	4.965465	0	0	0
**CS complex**	65	72	IREVVEEA	944.4977	4.993865	0.033	0.087093	0.03674
**CS complex**	65	72	IREVVEEA	944.4977	5.018127	1.667	0.156036	0.029724
**CS complex**	65	72	IREVVEEA	944.4977	4.942708	16.667002	0.561333	0.025834
**CS complex**	65	72	IREVVEEA	944.4977	5.029759	60.000004	0.853737	0.051435
**CS complex**	65	72	IREVVEEA	944.4977	5.030396	720.000061	1.668688	0.027485
**CysE**	65	72	IREVVEEA	944.4977	4.962636	0	0	0
**CysE**	65	72	IREVVEEA	944.4977	4.991487	0.033	0.107872	0.051976
**CysE**	65	72	IREVVEEA	944.4977	5.026429	1.667	0.189547	0.051249
**CysE**	65	72	IREVVEEA	944.4977	4.965959	16.667002	0.641956	0.082939
**CysE**	65	72	IREVVEEA	944.4977	5.028157	60.000004	0.860878	0.039963
**CysE**	65	72	IREVVEEA	944.4977	5.031878	720.000061	1.700924	0.058952
**maxD control**	65	72	IREVVEEA	944.4977	4.965549	0	0	0
**maxD control**	65	72	IREVVEEA	944.4977	5.156855	720.000061	3.795373	0.181184
**CS complex**	65	79	IREVVEEAYAADPEM	1721.7977	6.938056	0	0	0
**CS complex**	65	79	IREVVEEAYAADPEM	1721.7977	6.974131	0.033	0.519466	0.03325
**CS complex**	65	79	IREVVEEAYAADPEM	1721.7977	7.012214	1.667	1.076129	0.00332
**CS complex**	65	79	IREVVEEAYAADPEM	1721.7977	6.936759	16.667002	2.036296	2.27E-13
**CS complex**	65	79	IREVVEEAYAADPEM	1721.7977	7.012824	60.000004	2.607578	2.27E-13
**CS complex**	65	79	IREVVEEAYAADPEM	1721.7977	7.000940	720.000061	4.146935	2.27E-13
**CysE**	65	79	IREVVEEAYAADPEM	1721.7977	6.950388	0	0	0
**CysE**	65	79	IREVVEEAYAADPEM	1721.7977	6.978218	0.033	0.434388	0.044312
**CysE**	65	79	IREVVEEAYAADPEM	1721.7977	7.025629	1.667	1.036417	0.017544
**CysE**	65	79	IREVVEEAYAADPEM	1721.7977	6.947418	16.667002	1.764276	0
**CysE**	65	79	IREVVEEAYAADPEM	1721.7977	7.023914	60.000004	2.521505	0
**CysE**	65	79	IREVVEEAYAADPEM	1721.7977	7.008765	720.000061	4.102566	0
**maxD control**	65	79	IREVVEEAYAADPEM	1721.7977	6.938056	0	0	0
**maxD control**	65	79	IREVVEEAYAADPEM	1721.7977	7.124280	720.000061	7.880371	0.240792
**CS complex**	68	72	VVEEA	546.27	3.813607	0	0	0
**CS complex**	68	72	VVEEA	546.27	3.832650	0.033	0.178669	0.008952
**CS complex**	68	72	VVEEA	546.27	3.856294	1.667	0.142383	0.04843
**CS complex**	68	72	VVEEA	546.27	3.881540	60.000004	0.194689	0
**CS complex**	68	72	VVEEA	546.27	3.879535	720.000061	0.472839	0

**CysE**	68	72	VVEEA	546.27	3.799892	0	0	0
**CysE**	68	72	VVEEA	546.27	3.833848	0.033	0.151033	0.024963
**CysE**	68	72	VVEEA	546.27	3.855522	1.667	0.090209	0.034014
**CysE**	68	72	VVEEA	546.27	3.808296	16.667002	0.072785	0
**CysE**	68	72	VVEEA	546.27	3.852718	60.000004	0.116759	0
**CysE**	68	72	VVEEA	546.27	3.878458	720.000061	0.52048	0
**maxD control**	68	72	VVEEA	546.27	3.813607	0	0	0
**maxD control**	68	72	VVEEA	546.27	3.969967	720.000061	1.641799	0.002317
**CS complex**	71	79	EAYAADPEM	996.3906	6.192480	0	0	0
**CS complex**	71	79	EAYAADPEM	996.3906	6.230395	0.033	0.491119	0.028133
**CS complex**	71	79	EAYAADPEM	996.3906	6.257626	1.667	0.985103	0.029934
**CS complex**	71	79	EAYAADPEM	996.3906	6.217056	16.667002	1.570266	0.025894
**CS complex**	71	79	EAYAADPEM	996.3906	6.266092	60.000004	1.70847	0.025894
**CS complex**	71	79	EAYAADPEM	996.3906	6.261639	720.000061	2.439032	0.025894
**CysE**	71	79	EAYAADPEM	996.3906	6.194692	0	0	0
**CysE**	71	79	EAYAADPEM	996.3906	6.226396	0.033	0.389347	0.017663
**CysE**	71	79	EAYAADPEM	996.3906	6.260758	1.667	0.975863	0.014261
**CysE**	71	79	EAYAADPEM	996.3906	6.195003	16.667002	1.559663	0
**CysE**	71	79	EAYAADPEM	996.3906	6.266396	60.000004	1.673067	0
**CysE**	71	79	EAYAADPEM	996.3906	6.259802	720.000061	2.480051	0
**maxD control**	71	79	EAYAADPEM	996.3906	6.191956	0	0	0
**maxD control**	71	79	EAYAADPEM	996.3906	6.344643	720.000061	3.434173	0.115907
**CS complex**	80	86	IASAACD	650.281402	3.932478	0	0	0
**CS complex**	80	86	IASAACD	650.281402	3.953458	0.033	0.385058	0.016095
**CS complex**	80	86	IASAACD	650.281402	3.973089	1.667	0.394418	0.01633
**CS complex**	80	86	IASAACD	650.281402	3.995011	60.000004	1.036153	0
**CS complex**	80	86	IASAACD	650.281402	3.992407	720.000061	1.229871	0
**CysE**	80	86	IASAACD	650.281402	3.918307	0	0	0
**CysE**	80	86	IASAACD	650.281402	3.944781	0.033	0.357104	0.028357
**CysE**	80	86	IASAACD	650.281402	3.974216	1.667	0.409525	0.013442
**CysE**	80	86	IASAACD	650.281402	3.925977	16.667002	0.629732	0
**CysE**	80	86	IASAACD	650.281402	3.968903	60.000004	0.863147	0
**CysE**	80	86	IASAACD	650.281402	3.992925	720.000061	1.25247	0
**maxD control**	80	86	IASAACD	650.281402	3.932478	0	0	0
**maxD control**	80	86	IASAACD	650.281402	4.078146	720.000061	3.146328	0.067116
**CS complex**	86	105	DIQAVRTRDPAVDKYSTPLL	2258.2063	6.729254	0	0	0
**CS complex**	86	105	DIQAVRTRDPAVDKYSTPLL	2258.2063	6.766695	0.033	1.307496	0.006859
**CS complex**	86	105	DIQAVRTRDPAVDKYSTPLL	2258.2063	6.803238	1.667	2.545927	0.006524
**CS complex**	86	105	DIQAVRTRDPAVDKYSTPLL	2258.2063	6.727795	16.667002	4.226168	0
**CS complex**	86	105	DIQAVRTRDPAVDKYSTPLL	2258.2063	6.802518	60.000004	5.586864	0
**CS complex**	86	105	DIQAVRTRDPAVDKYSTPLL	2258.2063	6.799905	720.000061	6.803494	0

**CysE**	86	105	DIQAVRTRDPAVDKYSTPLL	2258.2063	6.745653	0	0	0
**CysE**	86	105	DIQAVRTRDPAVDKYSTPLL	2258.2063	6.775190	0.033	1.216684	0.091381
**CysE**	86	105	DIQAVRTRDPAVDKYSTPLL	2258.2063	6.819416	1.667	2.279581	0.053932
**CysE**	86	105	DIQAVRTRDPAVDKYSTPLL	2258.2063	6.743486	16.667002	3.109525	0
**CysE**	86	105	DIQAVRTRDPAVDKYSTPLL	2258.2063	6.819072	60.000004	4.074766	0
**CysE**	86	105	DIQAVRTRDPAVDKYSTPLL	2258.2063	6.817112	720.000061	6.489045	0
**maxD control**	86	105	DIQAVRTRDPAVDKYSTPLL	2258.2063	6.729254	0	0	0
**maxD control**	86	105	DIQAVRTRDPAVDKYSTPLL	2258.2063	6.987923	720.000061	10.051522	0.246337
**CS complex**	87	105	IQAVRTRDPAVDKYSTPLL	2143.1794	6.488885	0	0	0
**CS complex**	87	105	IQAVRTRDPAVDKYSTPLL	2143.1794	6.533795	0.033	1.272976	0.01881
**CS complex**	87	105	IQAVRTRDPAVDKYSTPLL	2143.1794	6.575685	1.667	2.445936	0.044095
**CS complex**	87	105	IQAVRTRDPAVDKYSTPLL	2143.1794	6.480123	16.667002	4.192763	0
**CS complex**	87	105	IQAVRTRDPAVDKYSTPLL	2143.1794	6.575495	60.000004	5.334857	0
**CS complex**	87	105	IQAVRTRDPAVDKYSTPLL	2143.1794	6.580606	720.000061	6.560287	0
**CysE**	87	105	IQAVRTRDPAVDKYSTPLL	2143.1794	6.488163	0	0	0
**CysE**	87	105	IQAVRTRDPAVDKYSTPLL	2143.1794	6.521834	0.033	1.195433	0.047412
**CysE**	87	105	IQAVRTRDPAVDKYSTPLL	2143.1794	6.571817	1.667	2.285001	0.013337
**CysE**	87	105	IQAVRTRDPAVDKYSTPLL	2143.1794	6.489013	16.667002	3.118819	0
**CysE**	87	105	IQAVRTRDPAVDKYSTPLL	2143.1794	6.569075	60.000004	4.289093	0
**CysE**	87	105	IQAVRTRDPAVDKYSTPLL	2143.1794	6.574744	720.000061	6.372946	0
**maxD control**	87	105	IQAVRTRDPAVDKYSTPLL	2143.1794	6.488885	0	0	0
**maxD control**	87	105	IQAVRTRDPAVDKYSTPLL	2143.1794	6.782249	720.000061	9.190296	0.231712
**CS complex**	89	105	AVRTRDPAVDKYSTPLL	1902.0367	6.365633	0	0	0
**CS complex**	89	105	AVRTRDPAVDKYSTPLL	1902.0367	6.402396	0.033	1.293477	0.036103
**CS complex**	89	105	AVRTRDPAVDKYSTPLL	1902.0367	6.442180	1.667	2.523024	0.054329
**CS complex**	89	105	AVRTRDPAVDKYSTPLL	1902.0367	6.361327	16.667002	4.206604	0.119725
**CS complex**	89	105	AVRTRDPAVDKYSTPLL	1902.0367	6.441222	60.000004	5.376622	0.044062
**CS complex**	89	105	AVRTRDPAVDKYSTPLL	1902.0367	6.443223	720.000061	6.337657	0.131836
**CysE**	89	105	AVRTRDPAVDKYSTPLL	1902.0367	6.371795	0	0	0
**CysE**	89	105	AVRTRDPAVDKYSTPLL	1902.0367	6.404743	0.033	1.155802	0.060006
**CysE**	89	105	AVRTRDPAVDKYSTPLL	1902.0367	6.452652	1.667	2.313499	0.030102
**CysE**	89	105	AVRTRDPAVDKYSTPLL	1902.0367	6.375113	16.667002	3.255363	0.019852
**CysE**	89	105	AVRTRDPAVDKYSTPLL	1902.0367	6.451289	60.000004	4.397355	0.009804
**CysE**	89	105	AVRTRDPAVDKYSTPLL	1902.0367	6.450392	720.000061	6.304395	0.048241
**maxD control**	89	105	AVRTRDPAVDKYSTPLL	1902.0367	6.365685	0	0	0
**maxD control**	89	105	AVRTRDPAVDKYSTPLL	1902.0367	6.681698	720.000061	7.609399	0.189692
**CS complex**	90	105	VRTRDPAVDKYSTPLL	1830.9996	6.358395	0	0	0
**CS complex**	90	105	VRTRDPAVDKYSTPLL	1830.9996	6.393008	0.033	1.035319	0.02614
**CS complex**	90	105	VRTRDPAVDKYSTPLL	1830.9996	6.437656	1.667	2.370633	0.026211
**CS complex**	90	105	VRTRDPAVDKYSTPLL	1830.9996	6.351736	16.667002	3.624082	0
**CS complex**	90	105	VRTRDPAVDKYSTPLL	1830.9996	6.437109	60.000004	4.672686	0

**CS complex**	90	105	VRTRDPAVDKYSTPLL	1830.9996	6.438895	720.000061	5.473212	0
**CysE**	90	105	VRTRDPAVDKYSTPLL	1830.9996	6.366014	0	0	0
**CysE**	90	105	VRTRDPAVDKYSTPLL	1830.9996	6.406236	0.033	0.895593	0.035016
**CysE**	90	105	VRTRDPAVDKYSTPLL	1830.9996	6.448430	1.667	1.991669	0.081931
**CysE**	90	105	VRTRDPAVDKYSTPLL	1830.9996	6.366970	16.667002	2.928505	0
**CysE**	90	105	VRTRDPAVDKYSTPLL	1830.9996	6.444946	60.000004	3.719165	0
**CysE**	90	105	VRTRDPAVDKYSTPLL	1830.9996	6.446115	720.000061	5.395822	0
**maxD control**	90	105	VRTRDPAVDKYSTPLL	1830.9996	6.358395	0	0	0
**maxD control**	90	105	VRTRDPAVDKYSTPLL	1830.9996	6.677875	720.000061	6.524475	0.112853
**CS complex**	106	115	YLKGFHALQA	1147.6183	6.362812	0	0	0
**CS complex**	106	115	YLKGFHALQA	1147.6183	6.401119	0.033	0.367783	0.24098
**CS complex**	106	115	YLKGFHALQA	1147.6183	6.442461	1.667	0.689731	0.184783
**CS complex**	106	115	YLKGFHALQA	1147.6183	6.359974	16.667002	0.950945	0.151734
**CS complex**	106	115	YLKGFHALQA	1147.6183	6.445597	60.000004	1.106482	0.192677
**CS complex**	106	115	YLKGFHALQA	1147.6183	6.444963	720.000061	1.419792	0.165689
**CysE**	106	115	YLKGFHALQA	1147.6183	6.371402	0	0	0
**CysE**	106	115	YLKGFHALQA	1147.6183	6.407389	0.033	0.373756	0.181151
**CysE**	106	115	YLKGFHALQA	1147.6183	6.451162	1.667	0.624593	0.111312
**CysE**	106	115	YLKGFHALQA	1147.6183	6.372169	16.667002	0.870355	0.102376
**CysE**	106	115	YLKGFHALQA	1147.6183	6.453481	60.000004	1.107121	0.111131
**CysE**	106	115	YLKGFHALQA	1147.6183	6.450871	720.000061	1.308865	0.109627
**maxD control**	106	115	YLKGFHALQA	1147.6183	6.362819	0	0	0
**maxD control**	106	115	YLKGFHALQA	1147.6183	6.631169	720.000061	3.930527	0.158413
**CS complex**	106	133	YLKGFHALQAYRIGHWLWNQGRRALAIF	3385.833384	9.795223	0	0	0
**CS complex**	106	133	YLKGFHALQAYRIGHWLWNQGRRALAIF	3385.833384	9.777124	0.033	1.747651	0.136012
**CS complex**	106	133	YLKGFHALQAYRIGHWLWNQGRRALAIF	3385.833384	9.834195	1.667	3.499558	0.205322
**CS complex**	106	133	YLKGFHALQAYRIGHWLWNQGRRALAIF	3385.833384	9.706794	16.667002	4.85373	4.55E-13
**CS complex**	106	133	YLKGFHALQAYRIGHWLWNQGRRALAIF	3385.833384	9.830957	60.000004	5.312966	4.55E-13
**CS complex**	106	133	YLKGFHALQAYRIGHWLWNQGRRALAIF	3385.833384	9.810940	720.000061	6.352714	4.55E-13
**CysE**	106	133	YLKGFHALQAYRIGHWLWNQGRRALAIF	3385.833384	9.812201	0	0	0
**CysE**	106	133	YLKGFHALQAYRIGHWLWNQGRRALAIF	3385.833384	9.794626	0.033	1.674966	0.090171
**CysE**	106	133	YLKGFHALQAYRIGHWLWNQGRRALAIF	3385.833384	9.863458	1.667	3.611012	0.09544
**CysE**	106	133	YLKGFHALQAYRIGHWLWNQGRRALAIF	3385.833384	9.773822	16.667002	4.739224	0
**CysE**	106	133	YLKGFHALQAYRIGHWLWNQGRRALAIF	3385.833384	9.878263	60.000004	5.325754	0
**CysE**	106	133	YLKGFHALQAYRIGHWLWNQGRRALAIF	3385.833384	9.863440	720.000061	6.483525	0
**maxD control**	106	133	YLKGFHALQAYRIGHWLWNQGRRALAIF	3385.833384	9.856859	0	0	0
**maxD control**	106	133	YLKGFHALQAYRIGHWLWNQGRRALAIF	3385.833384	9.934566	720.000061	15.699789	0.073583
**CS complex**	116	126	YRIGHWLWNQG	1429.7051	8.100384	0	0	0
**CS complex**	116	126	YRIGHWLWNQG	1429.7051	8.150746	0.033	0.639331	0.03085
**CS complex**	116	126	YRIGHWLWNQG	1429.7051	8.192530	1.667	0.815079	0.021641
**CS complex**	116	126	YRIGHWLWNQG	1429.7051	8.090816	16.667002	1.302477	0

**CS complex**	116	126	YRIGHWLWNQG	1429.7051	8.195461	60.000004	1.802519	0
**CS complex**	116	126	YRIGHWLWNQG	1429.7051	8.192788	720.000061	1.943652	0
**CysE**	116	126	YRIGHWLWNQG	1429.7051	8.103643	0	0	0
**CysE**	116	126	YRIGHWLWNQG	1429.7051	8.143759	0.033	0.58759	0.030259
**CysE**	116	126	YRIGHWLWNQG	1429.7051	8.193997	1.667	0.843899	0.004286
**CysE**	116	126	YRIGHWLWNQG	1429.7051	8.100378	16.667002	1.315622	0
**CysE**	116	126	YRIGHWLWNQG	1429.7051	8.199827	60.000004	1.802703	0
**CysE**	116	126	YRIGHWLWNQG	1429.7051	8.191665	720.000061	2.013819	0
**maxD control**	116	126	YRIGHWLWNQG	1429.7051	8.100384	0	0	0
**maxD control**	116	126	YRIGHWLWNQG	1429.7051	8.355453	720.000061	5.249985	0.093745
**CS complex**	116	130	YRIGHWLWNQGRRAL	1926.0286	7.238129	0	0	0
**CS complex**	116	130	YRIGHWLWNQGRRAL	1926.0286	7.265226	0.033	1.582369	0.165107
**CS complex**	116	130	YRIGHWLWNQGRRAL	1926.0286	7.311738	1.667	2.979427	0.159665
**CS complex**	116	130	YRIGHWLWNQGRRAL	1926.0286	7.250785	16.667002	3.784768	0.186138
**CS complex**	116	130	YRIGHWLWNQGRRAL	1926.0286	7.312631	60.000004	4.548533	0.166659
**CS complex**	116	130	YRIGHWLWNQGRRAL	1926.0286	7.307870	720.000061	4.913806	0.172201
**CysE**	116	130	YRIGHWLWNQGRRAL	1926.0286	7.271700	0	0	0
**CysE**	116	130	YRIGHWLWNQGRRAL	1926.0286	7.287940	0.033	1.470785	0.130368
**CysE**	116	130	YRIGHWLWNQGRRAL	1926.0286	7.340618	1.667	3.037442	0.104067
**CysE**	116	130	YRIGHWLWNQGRRAL	1926.0286	7.252351	16.667002	3.868476	0.11775
**CysE**	116	130	YRIGHWLWNQGRRAL	1926.0286	7.343120	60.000004	4.501257	0.100274
**CysE**	116	130	YRIGHWLWNQGRRAL	1926.0286	7.327281	720.000061	5.01434	0.080828
**maxD control**	116	130	YRIGHWLWNQGRRAL	1926.0286	7.238129	0	0	0
**maxD control**	116	130	YRIGHWLWNQGRRAL	1926.0286	7.505730	720.000061	8.498508	0.18785
**CS complex**	116	131	YRIGHWLWNQGRRALA	1997.0657	7.209635	0	0	0
**CS complex**	116	131	YRIGHWLWNQGRRALA	1997.0657	7.235003	0.033	1.879453	0.02632
**CS complex**	116	131	YRIGHWLWNQGRRALA	1997.0657	7.278238	1.667	3.374014	0.029516
**CS complex**	116	131	YRIGHWLWNQGRRALA	1997.0657	7.200795	16.667002	4.391538	0
**CS complex**	116	131	YRIGHWLWNQGRRALA	1997.0657	7.270609	60.000004	5.015786	0
**CS complex**	116	131	YRIGHWLWNQGRRALA	1997.0657	7.264437	720.000061	5.645995	2.27E-13
**CysE**	116	131	YRIGHWLWNQGRRALA	1997.0657	7.248513	0	0	0
**CysE**	116	131	YRIGHWLWNQGRRALA	1997.0657	7.248825	0.033	1.617329	0.074255
**CysE**	116	131	YRIGHWLWNQGRRALA	1997.0657	7.308654	1.667	3.33041	0.00894
**CysE**	116	131	YRIGHWLWNQGRRALA	1997.0657	7.222836	16.667002	4.21731	0
**CysE**	116	131	YRIGHWLWNQGRRALA	1997.0657	7.315917	60.000004	4.999138	2.27E-13
**CysE**	116	131	YRIGHWLWNQGRRALA	1997.0657	7.291369	720.000061	5.743209	0
**maxD control**	116	131	YRIGHWLWNQGRRALA	1997.0657	7.210385	0	0	0
**maxD control**	116	131	YRIGHWLWNQGRRALA	1997.0657	7.458240	720.000061	9.8094	0.154208
**CS complex**	116	133	YRIGHWLWNQGRRALAIF	2257.218	8.767119	0	0	0
**CS complex**	116	133	YRIGHWLWNQGRRALAIF	2257.218	8.793997	0.033	1.527705	0.125642
**CS complex**	116	133	YRIGHWLWNQGRRALAIF	2257.218	8.842793	1.667	2.98529	0.122417

**CS complex**	116	133	YRIGHWLWNQGRRALAIF	2257.218	8.744150	16.667002	3.949659	0.119379
**CS complex**	116	133	YRIGHWLWNQGRRALAIF	2257.218	8.837549	60.000004	4.612847	0.120924
**CS complex**	116	133	YRIGHWLWNQGRRALAIF	2257.218	8.827524	720.000061	5.234106	0.122284
**CysE**	116	133	YRIGHWLWNQGRRALAIF	2257.218	8.826587	0	0	0
**CysE**	116	133	YRIGHWLWNQGRRALAIF	2257.218	8.813457	0.033	1.381447	0.121293
**CysE**	116	133	YRIGHWLWNQGRRALAIF	2257.218	8.881928	1.667	3.008124	0.099848
**CysE**	116	133	YRIGHWLWNQGRRALAIF	2257.218	8.784795	16.667002	3.911107	0.091792
**CysE**	116	133	YRIGHWLWNQGRRALAIF	2257.218	8.878711	60.000004	4.585041	0.09138
**CysE**	116	133	YRIGHWLWNQGRRALAIF	2257.218	8.844720	720.000061	5.442133	0.091295
**maxD control**	116	133	YRIGHWLWNQGRRALAIF	2257.218	8.767119	0	0	0
**maxD control**	116	133	YRIGHWLWNQGRRALAIF	2257.218	9.002043	720.000061	10.995793	0.157604
**CS complex**	121	133	WLWNQGRRALAIF	1630.8892	8.772659	0	0	0
**CS complex**	121	133	WLWNQGRRALAIF	1630.8892	8.798336	0.033	1.647079	0.057141
**CS complex**	121	133	WLWNQGRRALAIF	1630.8892	8.844321	1.667	3.16753	0.055545
**CS complex**	121	133	WLWNQGRRALAIF	1630.8892	8.751412	16.667002	3.91522	0
**CS complex**	121	133	WLWNQGRRALAIF	1630.8892	8.836378	60.000004	4.687993	0
**CS complex**	121	133	WLWNQGRRALAIF	1630.8892	8.826031	720.000061	5.424933	0
**CysE**	121	133	WLWNQGRRALAIF	1630.8892	8.833219	0	0	0
**CysE**	121	133	WLWNQGRRALAIF	1630.8892	8.816209	0.033	1.461008	0.085441
**CysE**	121	133	WLWNQGRRALAIF	1630.8892	8.889980	1.667	3.137409	0.045748
**CysE**	121	133	WLWNQGRRALAIF	1630.8892	8.786713	16.667002	3.851578	0
**CysE**	121	133	WLWNQGRRALAIF	1630.8892	8.886265	60.000004	4.664525	0
**CysE**	121	133	WLWNQGRRALAIF	1630.8892	8.851434	720.000061	5.405838	2.27E-13
**maxD control**	121	133	WLWNQGRRALAIF	1630.8892	8.772659	0	0	0
**maxD control**	121	133	WLWNQGRRALAIF	1630.8892	8.993083	720.000061	9.18011	0.015624
**CS complex**	122	133	LWNQGRRALAIF	1444.8098	8.078577	0	0	0
**CS complex**	122	133	LWNQGRRALAIF	1444.8098	8.123821	0.033	1.291927	0.113817
**CS complex**	122	133	LWNQGRRALAIF	1444.8098	8.166333	1.667	2.614106	0.06423
**CS complex**	122	133	LWNQGRRALAIF	1444.8098	8.066569	16.667002	3.230606	0.06202
**CS complex**	122	133	LWNQGRRALAIF	1444.8098	8.166559	60.000004	3.611768	0.067733
**CS complex**	122	133	LWNQGRRALAIF	1444.8098	8.166001	720.000061	4.055693	0.064848
**CysE**	122	133	LWNQGRRALAIF	1444.8098	8.081584	0	0	0
**CysE**	122	133	LWNQGRRALAIF	1444.8098	8.116261	0.033	1.166526	0.092306
**CysE**	122	133	LWNQGRRALAIF	1444.8098	8.168720	1.667	2.73814	0.057524
**CysE**	122	133	LWNQGRRALAIF	1444.8098	8.075969	16.667002	3.369843	0.039992
**CysE**	122	133	LWNQGRRALAIF	1444.8098	8.172064	60.000004	3.739602	0.039931
**CysE**	122	133	LWNQGRRALAIF	1444.8098	8.167765	720.000061	4.366741	0.039486
**maxD control**	122	133	LWNQGRRALAIF	1444.8098	8.078577	0	0	0
**maxD control**	122	133	LWNQGRRALAIF	1444.8098	8.355282	720.000061	6.090163	0.126945
**CS complex**	132	139	IFLQNQVS	948.514908	6.830243	0	0	0
**CS complex**	132	139	IFLQNQVS	948.514908	6.866086	0.033	0.196634	0.012004

**CS complex**	132	139	IFLQNQVS	948.514908	6.903675	1.667	0.183199	0.019125
**CS complex**	132	139	IFLQNQVS	948.514908	6.826653	16.667002	0.185761	0
**CS complex**	132	139	IFLQNQVS	948.514908	6.906715	60.000004	0.186086	0
**CS complex**	132	139	IFLQNQVS	948.514908	6.907108	720.000061	0.35464	0
**CysE**	132	139	IFLQNQVS	948.514908	6.834333	0	0	0
**CysE**	132	139	IFLQNQVS	948.514908	6.860864	0.033	0.18251	0.015187
**CysE**	132	139	IFLQNQVS	948.514908	6.905131	1.667	0.164212	0.006508
**CysE**	132	139	IFLQNQVS	948.514908	6.833754	16.667002	0.172728	0
**CysE**	132	139	IFLQNQVS	948.514908	6.909165	60.000004	0.198148	0
**CysE**	132	139	IFLQNQVS	948.514908	6.907566	720.000061	0.44067	0
**maxD control**	132	139	IFLQNQVS	948.514908	6.830243	0	0	0
**maxD control**	132	139	IFLQNQVS	948.514908	6.978339	720.000061	4.320385	0.092193
**CS complex**	134	141	LQNQVSVT	888.478522	4.858504	0	0	0
**CS complex**	134	141	LQNQVSVT	888.478522	4.893192	0.033	0.163201	0.00876
**CS complex**	134	141	LQNQVSVT	888.478522	4.915198	1.667	0.148009	0.008185
**CS complex**	134	141	LQNQVSVT	888.478522	4.875757	16.667002	-0.049459	0
**CS complex**	134	141	LQNQVSVT	888.478522	4.925848	60.000004	0.17555	0
**CS complex**	134	141	LQNQVSVT	888.478522	4.925358	720.000061	0.626214	0
**CysE**	134	141	LQNQVSVT	888.478522	4.864959	0	0	0
**CysE**	134	141	LQNQVSVT	888.478522	4.888153	0.033	0.133663	0.01478
**CysE**	134	141	LQNQVSVT	888.478522	4.918685	1.667	0.143127	0.008942
**CysE**	134	141	LQNQVSVT	888.478522	4.864706	16.667002	0.134477	0
**CysE**	134	141	LQNQVSVT	888.478522	4.918828	60.000004	0.195288	0
**CysE**	134	141	LQNQVSVT	888.478522	4.925485	720.000061	0.73635	0
**maxD control**	134	141	LQNQVSVT	888.478522	4.858504	0	0	0
**maxD control**	134	141	LQNQVSVT	888.478522	4.999744	720.000061	4.281985	0.066141
**CS complex**	134	142	LQNQVSVTF	1035.5393	7.562437	0	0	0
**CS complex**	134	142	LQNQVSVTF	1035.5393	7.600765	0.033	0.218483	0.072981
**CS complex**	134	142	LQNQVSVTF	1035.5393	7.635550	1.667	0.181212	0.075351
**CS complex**	134	142	LQNQVSVTF	1035.5393	7.557333	16.667002	0.13968	0.06337
**CS complex**	134	142	LQNQVSVTF	1035.5393	7.639611	60.000004	0.242007	0.038288
**CS complex**	134	142	LQNQVSVTF	1035.5393	7.639686	720.000061	0.566294	0.063432
**CysE**	134	142	LQNQVSVTF	1035.5393	7.564245	0	0	0
**CysE**	134	142	LQNQVSVTF	1035.5393	7.596256	0.033	0.178643	0.045678
**CysE**	134	142	LQNQVSVTF	1035.5393	7.638561	1.667	0.174009	0.058539
**CysE**	134	142	LQNQVSVTF	1035.5393	7.562744	16.667002	0.187529	0.031492
**CysE**	134	142	LQNQVSVTF	1035.5393	7.639510	60.000004	0.260774	0.018241
**CysE**	134	142	LQNQVSVTF	1035.5393	7.639040	720.000061	0.779917	0.114576
**maxD control**	134	142	LQNQVSVTF	1035.5393	7.562437	0	0	0
**maxD control**	134	142	LQNQVSVTF	1035.5393	7.720261	720.000061	5.003739	0.476774
**CS complex**	142	157	FQVDIHPAAKIGRGIM	1752.9501	6.648123	0	0	0

**CS complex**	142	157	FQVDIHPAAKIGRGIM	1752.9501	6.682240	0.033	0.715061	0.027124
**CS complex**	142	157	FQVDIHPAAKIGRGIM	1752.9501	6.720010	1.667	0.882716	0.092739
**CS complex**	142	157	FQVDIHPAAKIGRGIM	1752.9501	6.636593	16.667002	1.220274	0
**CS complex**	142	157	FQVDIHPAAKIGRGIM	1752.9501	6.722000	60.000004	1.636873	0
**CS complex**	142	157	FQVDIHPAAKIGRGIM	1752.9501	6.721589	720.000061	2.243886	0
**CysE**	142	157	FQVDIHPAAKIGRGIM	1752.9501	6.654206	0	0	0
**CysE**	142	157	FQVDIHPAAKIGRGIM	1752.9501	6.681703	0.033	0.738424	0.032185
**CysE**	142	157	FQVDIHPAAKIGRGIM	1752.9501	6.724729	1.667	0.974912	0.040792
**CysE**	142	157	FQVDIHPAAKIGRGIM	1752.9501	6.647218	16.667002	1.286342	0
**CysE**	142	157	FQVDIHPAAKIGRGIM	1752.9501	6.723758	60.000004	1.77447	0
**CysE**	142	157	FQVDIHPAAKIGRGIM	1752.9501	6.721147	720.000061	2.20667	0
**maxD control**	142	157	FQVDIHPAAKIGRGIM	1752.9501	6.648123	0	0	0
**maxD control**	142	157	FQVDIHPAAKIGRGIM	1752.9501	6.874048	720.000061	7.406546	0.133832
**CS complex**	143	155	QVDIHPAAKIGRG	1361.7572	4.346455	0	0	0
**CS complex**	143	155	QVDIHPAAKIGRG	1361.7572	4.370391	0.033	0.776536	0.040286
**CS complex**	143	155	QVDIHPAAKIGRG	1361.7572	4.397264	1.667	0.793009	0.05956
**CS complex**	143	155	QVDIHPAAKIGRG	1361.7572	4.405169	60.000004	1.613518	0
**CS complex**	143	155	QVDIHPAAKIGRG	1361.7572	4.409848	720.000061	2.170693	0
**CysE**	143	155	QVDIHPAAKIGRG	1361.7572	4.342233	0	0	0
**CysE**	143	155	QVDIHPAAKIGRG	1361.7572	4.364789	0.033	0.727017	0.053382
**CysE**	143	155	QVDIHPAAKIGRG	1361.7572	4.401897	1.667	0.883528	0.047305
**CysE**	143	155	QVDIHPAAKIGRG	1361.7572	4.345794	16.667002	1.148051	0
**CysE**	143	155	QVDIHPAAKIGRG	1361.7572	4.397573	60.000004	1.732338	0
**CysE**	143	155	QVDIHPAAKIGRG	1361.7572	4.411983	720.000061	2.143859	0
**maxD control**	143	155	QVDIHPAAKIGRG	1361.7572	4.346455	0	0	0
**maxD control**	143	155	QVDIHPAAKIGRG	1361.7572	4.574421	720.000061	5.597479	0.173203
**CS complex**	143	157	QVDIHPAAKIGRGIM	1605.8817	5.791147	0	0	0
**CS complex**	143	157	QVDIHPAAKIGRGIM	1605.8817	5.827230	0.033	0.69564	0.031675
**CS complex**	143	157	QVDIHPAAKIGRGIM	1605.8817	5.860959	1.667	0.83714	0.069439
**CS complex**	143	157	QVDIHPAAKIGRGIM	1605.8817	5.787943	16.667002	1.071148	0
**CS complex**	143	157	QVDIHPAAKIGRGIM	1605.8817	5.863525	60.000004	1.640683	0
**CS complex**	143	157	QVDIHPAAKIGRGIM	1605.8817	5.862033	720.000061	2.18065	0
**CysE**	143	157	QVDIHPAAKIGRGIM	1605.8817	5.814468	0	0	0
**CysE**	143	157	QVDIHPAAKIGRGIM	1605.8817	5.826048	0.033	0.669055	0.051363
**CysE**	143	157	QVDIHPAAKIGRGIM	1605.8817	5.864017	1.667	0.88008	0.0287
**CysE**	143	157	QVDIHPAAKIGRGIM	1605.8817	5.799564	16.667002	1.193789	0
**CysE**	143	157	QVDIHPAAKIGRGIM	1605.8817	5.867134	60.000004	1.681255	0
**CysE**	143	157	QVDIHPAAKIGRGIM	1605.8817	5.862919	720.000061	2.158917	0
**maxD control**	143	157	QVDIHPAAKIGRGIM	1605.8817	5.791156	0	0	0
**maxD control**	143	157	QVDIHPAAKIGRGIM	1605.8817	6.040558	720.000061	6.467893	0.124422
**CS complex**	143	158	QVDIHPAAKIGRGIML	1718.9657	6.658374	0	0	0

**CS complex**	143	158	QVDIHPAAKIGRGIML	1718.9657	6.697033	0.033	0.649066	0.19606
**CS complex**	143	158	QVDIHPAAKIGRGIML	1718.9657	6.737534	1.667	0.754457	0.163958
**CS complex**	143	158	QVDIHPAAKIGRGIML	1718.9657	6.649705	16.667002	1.027538	0.134584
**CS complex**	143	158	QVDIHPAAKIGRGIML	1718.9657	6.737700	60.000004	1.656371	0.144645
**CS complex**	143	158	QVDIHPAAKIGRGIML	1718.9657	6.738905	720.000061	2.749153	0.175396
**CysE**	143	158	QVDIHPAAKIGRGIML	1718.9657	6.667734	0	0	0
**CysE**	143	158	QVDIHPAAKIGRGIML	1718.9657	6.696957	0.033	0.733199	0.205289
**CysE**	143	158	QVDIHPAAKIGRGIML	1718.9657	6.744725	1.667	0.83386	0.16841
**CysE**	143	158	QVDIHPAAKIGRGIML	1718.9657	6.663952	16.667002	1.193213	0.173347
**CysE**	143	158	QVDIHPAAKIGRGIML	1718.9657	6.743862	60.000004	1.769902	0.143069
**CysE**	143	158	QVDIHPAAKIGRGIML	1718.9657	6.740066	720.000061	2.685018	0.143824
**maxD control**	143	158	QVDIHPAAKIGRGIML	1718.9657	6.658412	0	0	0
**maxD control**	143	158	QVDIHPAAKIGRGIML	1718.9657	6.926283	720.000061	7.06961	0.272762
**CS complex**	158	170	LDHATGIVVGETA	1282.6558	5.740103	0	0	0
**CS complex**	158	170	LDHATGIVVGETA	1282.6558	5.770627	0.033	0.462405	0.039454
**CS complex**	158	170	LDHATGIVVGETA	1282.6558	5.799544	1.667	0.718953	0.018823
**CS complex**	158	170	LDHATGIVVGETA	1282.6558	5.731633	16.667002	1.289501	0
**CS complex**	158	170	LDHATGIVVGETA	1282.6558	5.803695	60.000004	1.882219	0
**CS complex**	158	170	LDHATGIVVGETA	1282.6558	5.804687	720.000061	3.591528	2.27E-13
**CysE**	158	170	LDHATGIVVGETA	1282.6558	5.738864	0	0	0
**CysE**	158	170	LDHATGIVVGETA	1282.6558	5.769810	0.033	0.477968	0.01409
**CysE**	158	170	LDHATGIVVGETA	1282.6558	5.805236	1.667	0.725629	0.008558
**CysE**	158	170	LDHATGIVVGETA	1282.6558	5.743364	16.667002	1.082786	0
**CysE**	158	170	LDHATGIVVGETA	1282.6558	5.804096	60.000004	1.413935	0
**CysE**	158	170	LDHATGIVVGETA	1282.6558	5.806190	720.000061	3.305783	0
**maxD control**	158	170	LDHATGIVVGETA	1282.6558	5.740103	0	0	0
**maxD control**	158	170	LDHATGIVVGETA	1282.6558	5.925486	720.000061	6.345428	0.10492
**CS complex**	158	171	LDHATGIVVGETAV	1381.7241	6.452367	0	0	0
**CS complex**	158	171	LDHATGIVVGETAV	1381.7241	6.490381	0.033	0.349785	0.017144
**CS complex**	158	171	LDHATGIVVGETAV	1381.7241	6.525058	1.667	0.651001	0.025718
**CS complex**	158	171	LDHATGIVVGETAV	1381.7241	6.450401	16.667002	1.173317	0
**CS complex**	158	171	LDHATGIVVGETAV	1381.7241	6.525877	60.000004	1.830633	0
**CS complex**	158	171	LDHATGIVVGETAV	1381.7241	6.530898	720.000061	3.518259	0
**CysE**	158	171	LDHATGIVVGETAV	1381.7241	6.459604	0	0	0
**CysE**	158	171	LDHATGIVVGETAV	1381.7241	6.487522	0.033	0.434503	0.01859
**CysE**	158	171	LDHATGIVVGETAV	1381.7241	6.530730	1.667	0.734399	0.013821
**CysE**	158	171	LDHATGIVVGETAV	1381.7241	6.459141	16.667002	1.009879	0
**CysE**	158	171	LDHATGIVVGETAV	1381.7241	6.532380	60.000004	1.234133	0
**CysE**	158	171	LDHATGIVVGETAV	1381.7241	6.525830	720.000061	3.357079	0
**maxD control**	158	171	LDHATGIVVGETAV	1381.7241	6.455467	0	0	0
**maxD control**	158	171	LDHATGIVVGETAV	1381.7241	6.642299	720.000061	7.184814	0.786344

**CS complex**	159	170	DHATGIVVGETA	1169.5717	5.459922	0	0	0
**CS complex**	159	170	DHATGIVVGETA	1169.5717	5.488153	0.033	0.401165	0.025807
**CS complex**	159	170	DHATGIVVGETA	1169.5717	5.515390	1.667	0.82023	0.030264
**CS complex**	159	170	DHATGIVVGETA	1169.5717	5.445479	16.667002	1.186142	0
**CS complex**	159	170	DHATGIVVGETA	1169.5717	5.514440	60.000004	1.841606	0
**CS complex**	159	170	DHATGIVVGETA	1169.5717	5.514736	720.000061	3.506222	0
**CysE**	159	170	DHATGIVVGETA	1169.5717	5.462895	0	0	0
**CysE**	159	170	DHATGIVVGETA	1169.5717	5.485203	0.033	0.358476	0.010105
**CysE**	159	170	DHATGIVVGETA	1169.5717	5.517045	1.667	0.746755	0.024962
**CysE**	159	170	DHATGIVVGETA	1169.5717	5.460530	16.667002	1.041086	0
**CysE**	159	170	DHATGIVVGETA	1169.5717	5.514072	60.000004	1.378185	0
**CysE**	159	170	DHATGIVVGETA	1169.5717	5.514280	720.000061	3.215406	0
**maxD control**	159	170	DHATGIVVGETA	1169.5717	5.459922	0	0	0
**maxD control**	159	170	DHATGIVVGETA	1169.5717	5.623622	720.000061	6.310506	0.106375
**CS complex**	159	171	DHATGIVVGETAV	1268.64	6.269506	0	0	0
**CS complex**	159	171	DHATGIVVGETAV	1268.64	6.297100	0.033	0.47781	0.023572
**CS complex**	159	171	DHATGIVVGETAV	1268.64	6.332868	1.667	0.822832	0.029593
**CS complex**	159	171	DHATGIVVGETAV	1268.64	6.262785	16.667002	1.301928	0
**CS complex**	159	171	DHATGIVVGETAV	1268.64	6.330503	60.000004	1.841007	0
**CS complex**	159	171	DHATGIVVGETAV	1268.64	6.330071	720.000061	3.575103	0
**CysE**	159	171	DHATGIVVGETAV	1268.64	6.274085	0	0	0
**CysE**	159	171	DHATGIVVGETAV	1268.64	6.301421	0.033	0.437652	0.015379
**CysE**	159	171	DHATGIVVGETAV	1268.64	6.338590	1.667	0.775705	0.015322
**CysE**	159	171	DHATGIVVGETAV	1268.64	6.273538	16.667002	1.016128	0
**CysE**	159	171	DHATGIVVGETAV	1268.64	6.339990	60.000004	1.337025	0
**CysE**	159	171	DHATGIVVGETAV	1268.64	6.338533	720.000061	3.146221	0
**maxD control**	159	171	DHATGIVVGETAV	1268.64	6.269506	0	0	0
**maxD control**	159	171	DHATGIVVGETAV	1268.64	6.442096	720.000061	7.203351	0.149262
**CS complex**	164	170	IVVGETA	688.3795	5.157131	0	0	0
**CS complex**	164	170	IVVGETA	688.3795	5.183127	0.033	0.312881	0.028232
**CS complex**	164	170	IVVGETA	688.3795	5.207794	1.667	0.341391	0.042893
**CS complex**	164	170	IVVGETA	688.3795	5.148291	16.667002	0.431277	0
**CS complex**	164	170	IVVGETA	688.3795	5.214599	60.000004	0.802114	0
**CS complex**	164	170	IVVGETA	688.3795	5.215924	720.000061	1.748044	0
**CysE**	164	170	IVVGETA	688.3795	5.155179	0	0	0
**CysE**	164	170	IVVGETA	688.3795	5.181025	0.033	0.307612	0.008343
**CysE**	164	170	IVVGETA	688.3795	5.209269	1.667	0.249631	0.019911
**CysE**	164	170	IVVGETA	688.3795	5.156548	16.667002	0.32839	0
**CysE**	164	170	IVVGETA	688.3795	5.211220	60.000004	0.529976	0
**CysE**	164	170	IVVGETA	688.3795	5.216284	720.000061	1.661682	0
**maxD control**	164	170	IVVGETA	688.3795	5.157581	0	0	0

**maxD control**	164	170	IVVGETA	688.3795	5.296956	720.000061	3.065552	0.071653
**CS complex**	164	171	IVVGETAV	787.455996	6.219038	0	0	0
**CS complex**	164	171	IVVGETAV	787.455996	6.249925	0.033	0.204279	0.024498
**CS complex**	164	171	IVVGETAV	787.455996	6.283274	1.667	0.28982	0.038779
**CS complex**	164	171	IVVGETAV	787.455996	6.227014	16.667002	0.396992	0
**CS complex**	164	171	IVVGETAV	787.455996	6.284460	60.000004	0.772844	0
**CS complex**	164	171	IVVGETAV	787.455996	6.285326	720.000061	1.814168	0
**CysE**	164	171	IVVGETAV	787.455996	6.221143	0	0	0
**CysE**	164	171	IVVGETAV	787.455996	6.243808	0.033	0.240013	0.016252
**CysE**	164	171	IVVGETAV	787.455996	6.282530	1.667	0.294442	0.009134
**CysE**	164	171	IVVGETAV	787.455996	6.219118	16.667002	0.332658	0
**CysE**	164	171	IVVGETAV	787.455996	6.283464	60.000004	0.515051	0
**CysE**	164	171	IVVGETAV	787.455996	6.283787	720.000061	1.69914	0
**maxD control**	164	171	IVVGETAV	787.455996	6.219038	0	0	0
**maxD control**	164	171	IVVGETAV	787.455996	6.359829	720.000061	3.974241	0.086138
**CS complex**	178	203	ILQSVTLGGTGKSGGDRHPKIREGVM	2693.451543	5.306172	0	0	0
**CS complex**	178	203	ILQSVTLGGTGKSGGDRHPKIREGVM	2693.451543	5.329465	0.033	2.574023	0.055758
**CS complex**	178	203	ILQSVTLGGTGKSGGDRHPKIREGVM	2693.451543	5.360506	1.667	3.147428	0.061121
**CS complex**	178	203	ILQSVTLGGTGKSGGDRHPKIREGVM	2693.451543	5.274740	16.667002	3.489946	0
**CS complex**	178	203	ILQSVTLGGTGKSGGDRHPKIREGVM	2693.451543	5.366873	60.000004	3.71015	0
**CS complex**	178	203	ILQSVTLGGTGKSGGDRHPKIREGVM	2693.451543	5.369524	720.000061	4.400216	0
**CysE**	178	203	ILQSVTLGGTGKSGGDRHPKIREGVM	2693.451543	5.321419	0	0	0
**CysE**	178	203	ILQSVTLGGTGKSGGDRHPKIREGVM	2693.451543	5.338003	0.033	2.344055	0.049603
**CysE**	178	203	ILQSVTLGGTGKSGGDRHPKIREGVM	2693.451543	5.380247	1.667	2.963278	0.017697
**CysE**	178	203	ILQSVTLGGTGKSGGDRHPKIREGVM	2693.451543	5.327441	16.667002	3.17904	0
**CysE**	178	203	ILQSVTLGGTGKSGGDRHPKIREGVM	2693.451543	5.379437	60.000004	3.681963	0
**CysE**	178	203	ILQSVTLGGTGKSGGDRHPKIREGVM	2693.451543	5.383280	720.000061	4.204649	0
**maxD control**	178	203	ILQSVTLGGTGKSGGDRHPKIREGVM	2693.451543	5.306013	0	0	0
**maxD control**	178	203	ILQSVTLGGTGKSGGDRHPKIREGVM	2693.451543	5.583470	720.000061	11.391986	0.512055
**CS complex**	180	200	QSVTLGGTGKSGGDRHPKIRE	2180.1455	3.837411	0	0	0
**CS complex**	180	200	QSVTLGGTGKSGGDRHPKIRE	2180.1455	3.865340	0.033	2.656436	0.029456
**CS complex**	180	200	QSVTLGGTGKSGGDRHPKIRE	2180.1455	3.892581	1.667	3.195096	0.023482
**CS complex**	180	200	QSVTLGGTGKSGGDRHPKIRE	2180.1455	3.907054	60.000004	3.609127	0
**CS complex**	180	200	QSVTLGGTGKSGGDRHPKIRE	2180.1455	3.907717	720.000061	3.776427	0
**CysE**	180	200	QSVTLGGTGKSGGDRHPKIRE	2180.1455	3.839047	0	0	0
**CysE**	180	200	QSVTLGGTGKSGGDRHPKIRE	2180.1455	3.863169	0.033	2.267423	0.038014
**CysE**	180	200	QSVTLGGTGKSGGDRHPKIRE	2180.1455	3.899126	1.667	2.874512	0.018061
**CysE**	180	200	QSVTLGGTGKSGGDRHPKIRE	2180.1455	3.844814	16.667002	3.275898	0
**CysE**	180	200	QSVTLGGTGKSGGDRHPKIRE	2180.1455	3.896932	60.000004	3.409934	0
**CysE**	180	200	QSVTLGGTGKSGGDRHPKIRE	2180.1455	3.912613	720.000061	3.886343	0
**maxD control**	180	200	QSVTLGGTGKSGGDRHPKIRE	2180.1455	3.837411	0	0	0

**maxD control**	180	200	QSVTLGGTGKSGGDRHPKIRE	2180.1455	4.097144	720.000061	8.900822	0.22495
**CS complex**	180	203	QSVTLGGTGKSGGDRHPKIREGVM	2467.2756	4.631894	0	0	0
**CS complex**	180	203	QSVTLGGTGKSGGDRHPKIREGVM	2467.2756	4.661508	0.033	2.650306	0.062848
**CS complex**	180	203	QSVTLGGTGKSGGDRHPKIREGVM	2467.2756	4.688272	1.667	3.13683	0.046056
**CS complex**	180	203	QSVTLGGTGKSGGDRHPKIREGVM	2467.2756	4.601604	16.667002	3.322725	0
**CS complex**	180	203	QSVTLGGTGKSGGDRHPKIREGVM	2467.2756	4.697857	60.000004	3.40322	0
**CS complex**	180	203	QSVTLGGTGKSGGDRHPKIREGVM	2467.2756	4.699940	720.000061	4.099577	0
**CysE**	180	203	QSVTLGGTGKSGGDRHPKIREGVM	2467.2756	4.636514	0	0	0
**CysE**	180	203	QSVTLGGTGKSGGDRHPKIREGVM	2467.2756	4.665033	0.033	2.253453	0.040155
**CysE**	180	203	QSVTLGGTGKSGGDRHPKIREGVM	2467.2756	4.700042	1.667	2.84363	0.005561
**CysE**	180	203	QSVTLGGTGKSGGDRHPKIREGVM	2467.2756	4.644756	16.667002	3.254532	0
**CysE**	180	203	QSVTLGGTGKSGGDRHPKIREGVM	2467.2756	4.698737	60.000004	3.384986	0
**CysE**	180	203	QSVTLGGTGKSGGDRHPKIREGVM	2467.2756	4.699993	720.000061	3.929426	0
**maxD control**	180	203	QSVTLGGTGKSGGDRHPKIREGVM	2467.2756	4.631852	0	0	0
**maxD control**	180	203	QSVTLGGTGKSGGDRHPKIREGVM	2467.2756	4.922654	720.000061	10.317647	0.259753
**CS complex**	182	203	VTLGGTGKSGGDRHPKIREGVM	2252.185	4.518622	0	0	0
**CS complex**	182	203	VTLGGTGKSGGDRHPKIREGVM	2252.185	4.544490	0.033	2.660556	0.049668
**CS complex**	182	203	VTLGGTGKSGGDRHPKIREGVM	2252.185	4.577235	1.667	3.068675	0.083042
**CS complex**	182	203	VTLGGTGKSGGDRHPKIREGVM	2252.185	4.574583	16.667002	3.147418	0
**CS complex**	182	203	VTLGGTGKSGGDRHPKIREGVM	2252.185	4.581354	60.000004	3.338197	0
**CS complex**	182	203	VTLGGTGKSGGDRHPKIREGVM	2252.185	4.584891	720.000061	3.937987	0
**CysE**	182	203	VTLGGTGKSGGDRHPKIREGVM	2252.185	4.544349	0	0	0
**CysE**	182	203	VTLGGTGKSGGDRHPKIREGVM	2252.185	4.540627	0.033	2.374972	0.055766
**CysE**	182	203	VTLGGTGKSGGDRHPKIREGVM	2252.185	4.581348	1.667	2.997693	0.054938
**CysE**	182	203	VTLGGTGKSGGDRHPKIREGVM	2252.185	4.531347	16.667002	3.313457	0
**CysE**	182	203	VTLGGTGKSGGDRHPKIREGVM	2252.185	4.584319	60.000004	3.562641	0
**CysE**	182	203	VTLGGTGKSGGDRHPKIREGVM	2252.185	4.586170	720.000061	3.980451	0
**maxD control**	182	203	VTLGGTGKSGGDRHPKIREGVM	2252.185	4.518622	0	0	0
**maxD control**	182	203	VTLGGTGKSGGDRHPKIREGVM	2252.185	4.803380	720.000061	8.796922	0.315194
**CS complex**	204	215	IGAGAKILGNIE	1155.6658	6.885064	0	0	0
**CS complex**	204	215	IGAGAKILGNIE	1155.6658	6.922191	0.033	0.878852	0.197607
**CS complex**	204	215	IGAGAKILGNIE	1155.6658	6.960202	1.667	1.120248	0.170816
**CS complex**	204	215	IGAGAKILGNIE	1155.6658	6.876008	16.667002	1.332762	0.125705
**CS complex**	204	215	IGAGAKILGNIE	1155.6658	6.963686	60.000004	1.531281	0.12665
**CS complex**	204	215	IGAGAKILGNIE	1155.6658	6.959281	720.000061	2.925102	0.149551
**CysE**	204	215	IGAGAKILGNIE	1155.6658	6.892992	0	0	0
**CysE**	204	215	IGAGAKILGNIE	1155.6658	6.924011	0.033	0.995811	0.086648
**CysE**	204	215	IGAGAKILGNIE	1155.6658	6.963417	1.667	1.301989	0.033059
**CysE**	204	215	IGAGAKILGNIE	1155.6658	6.888335	16.667002	1.50964	0.017348
**CysE**	204	215	IGAGAKILGNIE	1155.6658	6.966253	60.000004	1.67629	0.013276
**CysE**	204	215	IGAGAKILGNIE	1155.6658	6.959252	720.000061	2.613366	0.02132

**maxD control**	204	215	IGAGAKILGNIE	1155.6658	6.885064	0	0	0
**maxD control**	204	215	IGAGAKILGNIE	1155.6658	7.072864	720.000061	5.414853	0.184477
**CS complex**	204	226	IGAGAKILGNIEVGRGAKIGAGS	2109.2065	6.475778	0	0	0
**CS complex**	204	226	IGAGAKILGNIEVGRGAKIGAGS	2109.2065	6.512307	0.033	2.069803	0.065453
**CS complex**	204	226	IGAGAKILGNIEVGRGAKIGAGS	2109.2065	6.552952	1.667	2.582501	0.136867
**CS complex**	204	226	IGAGAKILGNIEVGRGAKIGAGS	2109.2065	6.461250	16.667002	3.225284	0.012538
**CS complex**	204	226	IGAGAKILGNIEVGRGAKIGAGS	2109.2065	6.551670	60.000004	3.902389	0.036786
**CS complex**	204	226	IGAGAKILGNIEVGRGAKIGAGS	2109.2065	6.547249	720.000061	6.544977	0.019923
**CysE**	204	226	IGAGAKILGNIEVGRGAKIGAGS	2109.2065	6.485472	0	0	0
**CysE**	204	226	IGAGAKILGNIEVGRGAKIGAGS	2109.2065	6.511590	0.033	1.774942	0.151477
**CysE**	204	226	IGAGAKILGNIEVGRGAKIGAGS	2109.2065	6.559493	1.667	2.545396	0.062199
**CysE**	204	226	IGAGAKILGNIEVGRGAKIGAGS	2109.2065	6.478271	16.667002	3.056698	0.05565
**CysE**	204	226	IGAGAKILGNIEVGRGAKIGAGS	2109.2065	6.558675	60.000004	3.55348	0.043407
**CysE**	204	226	IGAGAKILGNIEVGRGAKIGAGS	2109.2065	6.550748	720.000061	5.707383	0.047242
**maxD control**	204	226	IGAGAKILGNIEVGRGAKIGAGS	2109.2065	6.475778	0	0	0
**maxD control**	204	226	IGAGAKILGNIEVGRGAKIGAGS	2109.2065	6.665468	720.000061	11.766082	0.457252
**CS complex**	204	227	IGAGAKILGNIEVGRGAKIGAGSV	2208.275	6.764555	0	0	0
**CS complex**	204	227	IGAGAKILGNIEVGRGAKIGAGSV	2208.275	6.805140	0.033	2.058228	0.050385
**CS complex**	204	227	IGAGAKILGNIEVGRGAKIGAGSV	2208.275	6.841062	1.667	2.636173	0.162749
**CS complex**	204	227	IGAGAKILGNIEVGRGAKIGAGSV	2208.275	6.753317	16.667002	3.310691	0.041695
**CS complex**	204	227	IGAGAKILGNIEVGRGAKIGAGSV	2208.275	6.840395	60.000004	4.05669	0.029055
**CS complex**	204	227	IGAGAKILGNIEVGRGAKIGAGSV	2208.275	6.827510	720.000061	7.196467	0.043492
**CysE**	204	227	IGAGAKILGNIEVGRGAKIGAGSV	2208.275	6.772441	0	0	0
**CysE**	204	227	IGAGAKILGNIEVGRGAKIGAGSV	2208.275	6.799876	0.033	1.891681	0.113391
**CysE**	204	227	IGAGAKILGNIEVGRGAKIGAGSV	2208.275	6.845758	1.667	2.665882	0.085063
**CysE**	204	227	IGAGAKILGNIEVGRGAKIGAGSV	2208.275	6.764353	16.667002	3.125149	0.065566
**CysE**	204	227	IGAGAKILGNIEVGRGAKIGAGSV	2208.275	6.845391	60.000004	3.709738	0.069083
**CysE**	204	227	IGAGAKILGNIEVGRGAKIGAGSV	2208.275	6.835033	720.000061	6.224897	0.065952
**maxD control**	204	227	IGAGAKILGNIEVGRGAKIGAGSV	2208.275	6.764555	0	0	0
**maxD control**	204	227	IGAGAKILGNIEVGRGAKIGAGSV	2208.275	6.957861	720.000061	12.537134	0.432417
**CS complex**	216	226	VGRGAKIGAGS	972.5509	3.085394	0	0	0
**CS complex**	216	226	VGRGAKIGAGS	972.5509	3.100825	0.033	1.409588	0.098361
**CS complex**	216	226	VGRGAKIGAGS	972.5509	3.143425	1.667	1.439346	0.085809
**CS complex**	216	226	VGRGAKIGAGS	972.5509	3.200018	60.000004	2.1396	0.080409
**CS complex**	216	226	VGRGAKIGAGS	972.5509	3.192777	720.000061	3.504442	0.066333
**CysE**	216	226	VGRGAKIGAGS	972.5509	3.065717	0	0	0
**CysE**	216	226	VGRGAKIGAGS	972.5509	3.080090	0.033	1.38134	0.097704
**CysE**	216	226	VGRGAKIGAGS	972.5509	3.132602	1.667	1.576107	0.025548
**CysE**	216	226	VGRGAKIGAGS	972.5509	3.026894	16.667002	1.671255	1.14E-13
**CysE**	216	226	VGRGAKIGAGS	972.5509	3.127177	60.000004	1.939744	1.14E-13
**CysE**	216	226	VGRGAKIGAGS	972.5509	3.189374	720.000061	3.183257	1.14E-13

**maxD control**	216	226	VGRGAKIGAGS	972.5509	3.085394	0	0	0
**maxD control**	216	226	VGRGAKIGAGS	972.5509	3.381032	720.000061	4.590414	0.090238
**CS complex**	216	227	VGRGAKIGAGSV	1071.6194	4.336511	0	0	0
**CS complex**	216	227	VGRGAKIGAGSV	1071.6194	4.369612	0.033	1.231312	0.037678
**CS complex**	216	227	VGRGAKIGAGSV	1071.6194	4.396417	1.667	1.338856	0.050037
**CS complex**	216	227	VGRGAKIGAGSV	1071.6194	4.403740	60.000004	2.191245	0
**CS complex**	216	227	VGRGAKIGAGSV	1071.6194	4.403144	720.000061	3.915682	0
**CysE**	216	227	VGRGAKIGAGSV	1071.6194	4.333884	0	0	0
**CysE**	216	227	VGRGAKIGAGSV	1071.6194	4.363672	0.033	1.134878	0.049255
**CysE**	216	227	VGRGAKIGAGSV	1071.6194	4.400042	1.667	1.364611	0.015252
**CysE**	216	227	VGRGAKIGAGSV	1071.6194	4.338809	16.667002	1.453498	0
**CysE**	216	227	VGRGAKIGAGSV	1071.6194	4.397491	60.000004	1.808138	0
**CysE**	216	227	VGRGAKIGAGSV	1071.6194	4.407406	720.000061	3.47113	0
**maxD control**	216	227	VGRGAKIGAGSV	1071.6194	4.336493	0	0	0
**maxD control**	216	227	VGRGAKIGAGSV	1071.6194	4.589683	720.000061	5.337751	0.077075
**CS complex**	227	238	VVLQPVPPHTTA	1258.7076	5.323607	0	0	0
**CS complex**	227	238	VVLQPVPPHTTA	1258.7076	5.349960	0.033	0.496973	0.010639
**CS complex**	227	238	VVLQPVPPHTTA	1258.7076	5.378372	1.667	1.734276	0.022534
**CS complex**	227	238	VVLQPVPPHTTA	1258.7076	5.307370	16.667002	3.076018	0
**CS complex**	227	238	VVLQPVPPHTTA	1258.7076	5.378505	60.000004	3.363804	0
**CS complex**	227	238	VVLQPVPPHTTA	1258.7076	5.384678	720.000061	3.99187	0
**CysE**	227	238	VVLQPVPPHTTA	1258.7076	5.324545	0	0	0
**CysE**	227	238	VVLQPVPPHTTA	1258.7076	5.347882	0.033	0.430929	0.06829
**CysE**	227	238	VVLQPVPPHTTA	1258.7076	5.381919	1.667	1.229427	0.086293
**CysE**	227	238	VVLQPVPPHTTA	1258.7076	5.326318	16.667002	2.281543	0
**CysE**	227	238	VVLQPVPPHTTA	1258.7076	5.380169	60.000004	2.766549	0
**CysE**	227	238	VVLQPVPPHTTA	1258.7076	5.385575	720.000061	3.810164	0
**maxD control**	227	238	VVLQPVPPHTTA	1258.7076	5.323671	0	0	0
**maxD control**	227	238	VVLQPVPPHTTA	1258.7076	5.503368	720.000061	4.879231	0
**CS complex**	228	257	VLQPVPPHTTAAGVPARIVGKPDSDKPSMD	3080.6116	5.285079	0	0	0
**CS complex**	228	257	VLQPVPPHTTAAGVPARIVGKPDSDKPSMD	3080.6116	5.315046	0.033	5.753193	0.205929
**CS complex**	228	257	VLQPVPPHTTAAGVPARIVGKPDSDKPSMD	3080.6116	5.328506	1.667	8.715434	0.242731
**CS complex**	228	257	VLQPVPPHTTAAGVPARIVGKPDSDKPSMD	3080.6116	5.249702	16.667002	10.824266	0
**CS complex**	228	257	VLQPVPPHTTAAGVPARIVGKPDSDKPSMD	3080.6116	5.324672	60.000004	11.534659	0
**CS complex**	228	257	VLQPVPPHTTAAGVPARIVGKPDSDKPSMD	3080.6116	5.332283	720.000061	11.762795	0
**CysE**	228	257	VLQPVPPHTTAAGVPARIVGKPDSDKPSMD	3080.6116	5.293715	0	0	0
**CysE**	228	257	VLQPVPPHTTAAGVPARIVGKPDSDKPSMD	3080.6116	5.309556	0.033	4.315214	0.291958
**CysE**	228	257	VLQPVPPHTTAAGVPARIVGKPDSDKPSMD	3080.6116	5.337757	1.667	7.798837	0.229
**CysE**	228	257	VLQPVPPHTTAAGVPARIVGKPDSDKPSMD	3080.6116	5.279510	16.667002	9.046267	0
**CysE**	228	257	VLQPVPPHTTAAGVPARIVGKPDSDKPSMD	3080.6116	5.335982	60.000004	10.551984	4.55E-13
**CysE**	228	257	VLQPVPPHTTAAGVPARIVGKPDSDKPSMD	3080.6116	5.330916	720.000061	11.840977	0

**maxD control**	228	257	VLQPVPPHTTAAGVPARIVGKPDSDKPSMD	3080.6116	5.287581	0	0	0
**maxD control**	228	257	VLQPVPPHTTAAGVPARIVGKPDSDKPSMD	3080.6116	5.475109	720.000061	11.525367	0.307694
**CS complex**	257	270	DMDQHFNGINHTFE	1704.6997	6.653273	0	0	0
**CS complex**	257	270	DMDQHFNGINHTFE	1704.6997	6.685496	0.033	3.947787	0.081993
**CS complex**	257	270	DMDQHFNGINHTFE	1704.6997	6.714159	1.667	4.419884	0.07909
**CS complex**	257	270	DMDQHFNGINHTFE	1704.6997	6.637389	16.667002	4.497848	2.27E-13
**CS complex**	257	270	DMDQHFNGINHTFE	1704.6997	6.720567	60.000004	4.673387	0
**CS complex**	257	270	DMDQHFNGINHTFE	1704.6997	6.719541	720.000061	4.438256	0
**CysE**	257	270	DMDQHFNGINHTFE	1704.6997	6.659238	0	0	0
**CysE**	257	270	DMDQHFNGINHTFE	1704.6997	6.683537	0.033	4.357184	0.035326
**CysE**	257	270	DMDQHFNGINHTFE	1704.6997	6.720406	1.667	4.553725	0.030182
**CysE**	257	270	DMDQHFNGINHTFE	1704.6997	6.648732	16.667002	4.548848	0
**CysE**	257	270	DMDQHFNGINHTFE	1704.6997	6.725189	60.000004	4.516958	0
**CysE**	257	270	DMDQHFNGINHTFE	1704.6997	6.722744	720.000061	4.747985	0
**maxD control**	257	270	DMDQHFNGINHTFE	1704.6997	6.653273	0	0	0
**maxD control**	257	270	DMDQHFNGINHTFE	1704.6997	6.850272	720.000061	4.343348	0.110475
**CS complex**	257	275	DMDQHFNGINHTFEYGDGI	2209.924358	7.662336	0	0	0
**CS complex**	257	275	DMDQHFNGINHTFEYGDGI	2209.924358	7.702767	0.033	4.680438	0.069
**CS complex**	257	275	DMDQHFNGINHTFEYGDGI	2209.924358	7.740368	1.667	6.259286	0.116866
**CS complex**	257	275	DMDQHFNGINHTFEYGDGI	2209.924358	7.651055	16.667002	6.625188	0
**CS complex**	257	275	DMDQHFNGINHTFEYGDGI	2209.924358	7.750062	60.000004	6.755555	0
**CS complex**	257	275	DMDQHFNGINHTFEYGDGI	2209.924358	7.748156	720.000061	6.514163	0
**CysE**	257	275	DMDQHFNGINHTFEYGDGI	2209.924358	7.667610	0	0	0
**CysE**	257	275	DMDQHFNGINHTFEYGDGI	2209.924358	7.689669	0.033	5.84038	0.058652
**CysE**	257	275	DMDQHFNGINHTFEYGDGI	2209.924358	7.735526	1.667	6.616583	0.023005
**CysE**	257	275	DMDQHFNGINHTFEYGDGI	2209.924358	7.654906	16.667002	6.547562	0
**CysE**	257	275	DMDQHFNGINHTFEYGDGI	2209.924358	7.740164	60.000004	6.693971	0
**CysE**	257	275	DMDQHFNGINHTFEYGDGI	2209.924358	7.737600	720.000061	6.819056	0
**maxD control**	257	275	DMDQHFNGINHTFEYGDGI	2209.924358	7.662336	0	0	0
**maxD control**	257	275	DMDQHFNGINHTFEYGDGI	2209.924358	7.867078	720.000061	6.658603	0.185073
**CS complex**	258	275	MDQHFNGINHTFEYGDGI	2094.897415	7.407113	0	0	0
**CS complex**	258	275	MDQHFNGINHTFEYGDGI	2094.897415	7.451242	0.033	4.18607	0.027696
**CS complex**	258	275	MDQHFNGINHTFEYGDGI	2094.897415	7.486201	1.667	5.476443	0.096074
**CS complex**	258	275	MDQHFNGINHTFEYGDGI	2094.897415	7.394649	16.667002	5.902637	0
**CS complex**	258	275	MDQHFNGINHTFEYGDGI	2094.897415	7.492243	60.000004	6.084553	0
**CS complex**	258	275	MDQHFNGINHTFEYGDGI	2094.897415	7.492518	720.000061	6.041889	0
**CysE**	258	275	MDQHFNGINHTFEYGDGI	2094.897415	7.411110	0	0	0
**CysE**	258	275	MDQHFNGINHTFEYGDGI	2094.897415	7.440552	0.033	5.415646	0.07268
**CysE**	258	275	MDQHFNGINHTFEYGDGI	2094.897415	7.486004	1.667	6.11022	0.040618
**CysE**	258	275	MDQHFNGINHTFEYGDGI	2094.897415	7.404636	16.667002	5.900985	0
**CysE**	258	275	MDQHFNGINHTFEYGDGI	2094.897415	7.489610	60.000004	6.110891	0

**CysE**	258	275	MDQHFNGINHTFEYGDGI	2094.897415	7.491089	720.000061	6.307726	0
**maxD control**	258	275	MDQHFNGINHTFEYGDGI	2094.897415	7.407113	0	0	0
**maxD control**	258	275	MDQHFNGINHTFEYGDGI	2094.897415	7.305675	720.000061	11.917268	2.100341
**CS complex**	259	270	DQHFNGINHTFE	1458.6322	6.153506	0	0	0
**CS complex**	259	270	DQHFNGINHTFE	1458.6322	6.189175	0.033	3.106001	0.08221
**CS complex**	259	270	DQHFNGINHTFE	1458.6322	6.217117	1.667	3.626852	0.071075
**CS complex**	259	270	DQHFNGINHTFE	1458.6322	6.136582	16.667002	3.683053	0.031236
**CS complex**	259	270	DQHFNGINHTFE	1458.6322	6.223611	60.000004	3.798475	0.030544
**CS complex**	259	270	DQHFNGINHTFE	1458.6322	6.222618	720.000061	3.607945	0.041498
**CysE**	259	270	DQHFNGINHTFE	1458.6322	6.159323	0	0	0
**CysE**	259	270	DQHFNGINHTFE	1458.6322	6.185602	0.033	3.776969	0.061491
**CysE**	259	270	DQHFNGINHTFE	1458.6322	6.220366	1.667	3.832016	0.046529
**CysE**	259	270	DQHFNGINHTFE	1458.6322	6.151951	16.667002	3.836432	0.024207
**CysE**	259	270	DQHFNGINHTFE	1458.6322	6.225745	60.000004	3.819057	0.05856
**CysE**	259	270	DQHFNGINHTFE	1458.6322	6.221346	720.000061	3.959361	0.024371
**maxD control**	259	270	DQHFNGINHTFE	1458.6322	6.153506	0	0	0
**maxD control**	259	270	DQHFNGINHTFE	1458.6322	6.364466	720.000061	3.512394	0.094754
